# Investigation of Gene Sequence Divergence, Expression Dynamics, and Endocrine Regulation of the Vitellogenin Gene Family in the Whiteleg Shrimp *Litopenaeus vannamei*

**DOI:** 10.3389/fendo.2020.577745

**Published:** 2020-11-19

**Authors:** Wei Wang, Bin Li, Tingting Zhou, Chenggui Wang, Amankwah Beatrice Kyei, Lili Shi, Siuming Chan

**Affiliations:** College of Fisheries, Guangdong Ocean University, Zhanjiang, China

**Keywords:** vitellogenin, shrimp, expression dynamics, gene divergence, ovary

## Abstract

In this report, we studied the vitellogenin gene family in the whiteleg shrimp *Litopenaeus vannamei* by transcriptomics, bioinformatics, and molecular biology methods. At least three moderately homologous vitellogenin (Vg) genes (i.e. *LvVg1*, *LvVg2*, and *LvVg3*) were identified in the genome. The deduced LvVg proteins consisted of a vitellogenin_N domain, a DUF1943 domain, and a VWD domain typical of most vitellogenins from oviparous animals. *LvVg1* was the most abundant *Vg* expressed in the hepatopancreas and ovary of maturing females. Furthermore, multiple isoforms of *LvVg1* were evolved presumably due to the need for rapid Vg production during the rapid phase of vitellogenesis. *LvVg* transcripts were detected in different larval stages, juveniles, and subadults. During the non-reproductive cycle, *LvVg* expression in the hepatopancreas peaked at the intermolt stages. During the female vitellogenesis cycle, a two-phase expression pattern of *LvVg1* gene was observed in the hepatopancreas and ovary. Moreover, the eyestalk optic nerve, brain, and thoracic ganglion consisted of factors that differentially regulated the expression of the three *Vg* genes. In addition to their reproduction-related roles, Vg may also be involved in growth and molt-related processes. Phylogenetic analysis revealed the early expansion and separation of these *Vg* genes, and it is most likely correlated with the expansion of *Vg*’s function. In conclusion, the evolution of multiple *LvVg1* isoforms and the acquisition of different *Vg* genes (i.e. *LvVg2* and *LvVg3*) may occur universally in most decapods. Full information on the total number of *Vg* genes and precise knowledge on the expression pattern and endocrine regulation of each *Vg* during all life cycle stages are crucial for us to understand the roles of this emerging gene family in the control of shrimp reproduction and other non-reproductive processes.

## Introduction

**Figure 1:**
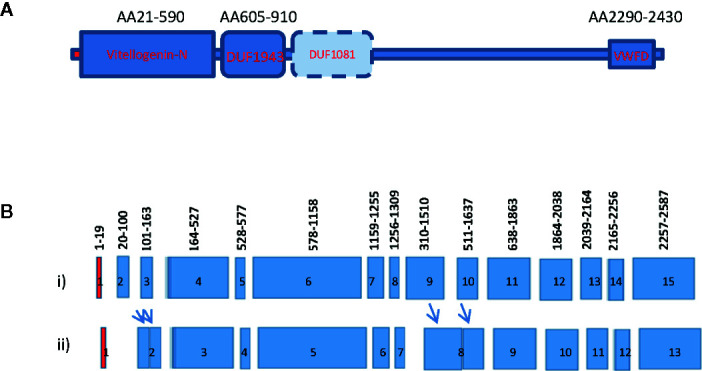
Gene structure and domain organization of vitellogenin of decapod crustaceans. **(A)** Vitellogenin gene structure of decapod crustaceans. The *Vg* of crustacean consists of the N-terminal vitellogenin-N domain, a domain of unknown function 1943 (DUF1943), occasionally a domain of unknown function 1081 (DUF1081), and a C-terminal vertebrate Von Willebrand D domain (VWFD) (1–2); **(B)** Intron and exon gene organization of shrimp vitellogenin. Exons are indicated by the colored boxes and introns are represented by gaps between neighboring exons. Most crustacean *Vg* genes consist of 15 exons interrupted by 14 introns (i). Only a few Vg genes consist of less than 15 exons (ii). The red boxes represent exon1 that encodes for the signal peptide of the *Vgs*. The numbers above each exon indicate the position of the amino acid encoded by the exons. The relative position of these intron/exon boundaries are also conserved in many reported *Vg* genes (3–4). Arrows indicate fusion of the exons to form a larger exon.

Growth and reproduction are two energy-requiring processes important for the species’ continuation. During female maturation of shrimp, the ovary undergoes rapid vitellogenesis from the synthesis of a large quantity of the major egg yolk protein or vitellogenin (Vg). As in other oviparous animals, shrimp vitellogenins (Vgs) are large lipophospoproteins with several conserved domains including the N-terminal LNP domain, domain of unknown function DUF1940, and the von Willebrand factor type D domain (VWD) (**Figure 1A**). Vg is defined as a storage molecule with the main function to supply nutrients, energy, and raw materials to developing oocytes for embryonic and larval growth (1, 2). It was initially known to be expressed only in females (5, 6). After synthesis from its site(s), Vg is cleaved into several subunits and then transported to the ovary for uptake. Research on Vg in decapod crustaceans began with the purification of Vg from maturing ovary, followed by amino acid sequence determination of the protein (3, 7, 8). After that, degenerated primers were designed to clone a partial fragment of the Vg gene by RT-PCR. Eventually, the RACE and RT-PCR cloning approaches were used to obtain the full-length sequence of Vg (3, 7). Previous studies using molecular cloning approaches resulted in the identification of one shrimp vitellogenin gene. In subsequent studies based on genomic PCR cloning, genome walking and genomic library screening, the vitellogenin genes of several decapods have been reported (**Figure 1B**) (3, 4, 8–10).

Historically, the site of Vg synthesis in decapods has been an issue of debate as there are many conflicting results. Several tissues/organs have been reported as the sites of Vg synthesis in crustaceans. These sites include the sub-epidermal adipose tissue, the hepatopancreas, and the ovary (11–16). While the idea that Vg is synthesized by the sub-epidermal adipose tissues has not gained support in recent years due to the lack of further evidence, there are still back-and-forth arguments for the ovary and/or hepatopancreas as the site of Vg synthesis. Recently, increasing evidence has indicated the existence of multiple Vg genes. For example, in the sand shrimp *Metapenaeus ensis*, two forms of *Vg* gene were reported (2, 3); in the fresh-water prawn *Macrobrachium rosenbergii*, four different *Vg* genes have been identified (10). Other decapods that have been reported to have more than one *Vg* gene include the whiteleg shrimp *Litopenaeus vannamei* and the red crab *Charybdis feriatus* (9, 16, 17). The controversy in the source of Vg synthesis might be due to stage-specific expression pattern of *Vg* genes. In short, without full knowledge of the total number of vitellogenin genes, it would be difficult to obtain precise expression information of *Vg*(s).

Concerning the endocrine regulation of vitellogenesis in crustacean, it is well established that the eyestalk and nervous tissues such as brain and thoracic ganglion produce neuro-endocrine factors that regulate vitellogenesis. For example, eyestalk ablation can cause precocious ovary maturation in shrimp and the brain of shrimp may consist of factors that can stimulate oogenesis.

Recently, the role of vitellogenin as a reproduction-related protein has been expanded. Some studies reported that, as in vertebrates, Vg can also be detected in male individuals under environmental stress. Furthermore, some studies also reported the expression of *Vg* in non-reproductive tissues and these *Vgs* may have an antimicrobial function or immune-related functions (18–21). For example, in the crab, a *Vg2* cDNA was detected in the hemocyte and the function of the *Vg2* appeared to be immunity-related (17, 18). Knowledge of the structure, organization, and promoter sequence information of vitellogenin is important for us to understand the evolution and regulation of *Vg* genes (10).

The whiteleg shrimp *L. vannamei* is the dominant shrimp species cultured worldwide. The production of shrimp fry from the hatchery is hampered by the lack of information on the endocrine regulation of female vitellogenesis and reproduction (16). Therefore, manipulation of female maturation has not been successful and shrimp hatcheries have to rely on the undesirable and harmful technique of eyestalk ablation to stimulate female maturation (2). Unilateral eyestalk ablation is used to remove the source of a gonad inhibition hormone leading to the stimulation of gonad maturation or rapid vitellogenesis. The drawback of eyestalk ablation is the removal of other endocrine factors associated with the eyestalk X-organ sinus gland complex. As a result, the quality of the egg produced is inferior (17). Although a *LvVg* cDNA has already been reported in the whiteleg shrimp *L. vannamei* (16), the finding that this *Vg* represents only a member of the *Vg* gene family leads us to reconsider its contribution in vitellogenesis and the evolution of different members involved in gonad maturation. In this study, we used transcriptomic, bioinformatic, and molecular biology approaches to characterize several vitellogenin genes from the whiteleg shrimp *L. vanname*i. The genomic structures and phylogenetic relationships of various *Vg* genes were analyzed, and expression patterns of these *Vg* genes during ovary maturation, at different ontogenic stages or during the molting cycle were investigated. Potential regulation of these *Vg* genes by the eyestalk, brain, and thoracic ganglion was also examined, with discussion on the roles of this emerging gene family in the control of shrimp reproduction and other non-reproductive processes.

## Materials and Methods

### Animals

Larval, juvenile, and adult whiteleg shrimp were obtained from the Donghai Island shrimp breeding and research center of Guangdong Ocean University (GDOU). Subadult (22–28 g) and adult (45–49 g) whiteleg shrimp *L. vannamei* were either obtained from our own culture facility or from local sea-food markets. All animals were cultured in indoor culture tank with natural lighting, in flow-through seawater at a temperature of 27–29°C and a salinity of 32–34‰. They were fed with either pellet diet (Yuehai Feed Group) or maturation diet (fresh squid and polychaetes) three times daily at a rate of 10% body weight.

### RNA Extraction, Transcriptome Sequencing, and *Vg* Homologs Identification

We have performed a transcriptome sequence analysis of the eyestalk, brain, hepatopancreas, and ovary of the whiteleg shrimp *L. vannamei*. Total RNAs were prepared by an RNA extraction kit (Trizol, Invitrogen Life Technology, CA, USA). Each shrimp was considered a separate sample and three samples were taken as biological replicates for each group, and an equal amount of RNA from three individuals was pooled to make a sample for library construction. RNA library construction and paired-end sequencing was carried out at the Gene Denovo Biotechnology Company (Guangzhou, China) using Illumina HiSeq™ 4000. After data filtering, the clean sequencing reads were subjected to transcriptome assembly using the Trinity software and associated packages. Sequences in the assembly homologous to vitellogenin genes were identified by Blast tools and used in following studies.

### Genomic PCR

Genomic DNA from individual shrimp was prepared either from muscles or hepatopancreas using a spin column based Genomic DNA preparation kit (Tiangen, China). After elution from the column, the concentration of the genomic DNA was determined by OD measurement with a Nano plus spectrophotometer (Thermo, Grand Island, NY, USA). For PCR, gene specific primers were designed. To avoid the designed primer being located in the junction of the intron/exon, we choose primers from the 5’ region of the exon-intron boundary.

### RT-PCR and *LvVg* cDNA Cloning

To validate the assembled sequences of the cDNA of *LvVg1*, *LvVg2*, and *LvVg3*, RT-PCR and cDNA cloning was performed. Based on *LvVg* gene sequences from the transcriptome data, specific primers were designed for the amplification of the *LvVg* cDNA. PCR was performed using 2× Taq PCR Master Mix (Tiangen, Beijing, China) for the validation of *LvVg1*, *LvVg2*, and *LvVg3* fragments. PCR reactions condition was as follows: Denaturation at 94°C for 3 min, followed by 35 cycles of 94°C for 30 s, 60°C for 30 s, and 72°C for 1 min. PCR products were further extended at 72°C for 5 min. For the purification and recovery of PCR products, a PCR product purification kit was used (Thermos, USA). The PCR product was then ligated to pMD20 vector (Invitrogen, USA). Afterwards, the transformation of Trans5a competent cells was conducted according to the manufacturer’s instructions (Tiangen, China). The positive clones were verified by PCR and sequenced, and overlapping fragments of the *Lv1Vg*, *Lv2Vg*, and *Lv3Vg* genes were obtained.

### Expression Patterns of *LvVgs*

To investigate the tissue specificity of *LvVg* expression, total RNAs were prepared from different tissues (including the epidermis, hepatopancreas, eyestalk, ovary, brain, nerve cord, thoracic ganglion, and muscle) from female adults. To examine gene expression changes during ontogeny, RNA was also extracted from the nauplius, zoea, mysis, as well as tissues of the juvenile and adult shrimp. Total RNAs were reversely transcribed to cDNA. Both β-actin and the elongation factor (EF-1a) were used as the internal references for the reverse transcription-polymerase chain reaction (RT-PCR) analysis. PCR reaction conditions were as follows: denaturation at 94°C for 3 min, followed by 34 cycles of 94°C for 30 s, 60°C for 30 s, and 72°C for 1 min. Finally, extension at 72°C for 5 min was conducted to complete the reactions. The PCR products were analyzed by 1% agarose gel electrophoresis.

In addition, total RNA was extracted from the hepatopancreas and ovaries of females at different maturation stages (gonadosomatic index [GSI] from 1–12%) and at various molt cycle stages (including A, B, C1–C3, D, and E). Quantitative real-time polymerase chain reaction (qRT-PCR) was conducted using the Bio-Rad and SYBR Premix Ex Taq II (TaKaRa, Tokyo, Japan) to investigate the expression patterns of *LvVgs* at different developmental stages, using β-actin and LvEF-1a as the internal control. The primers for the qRT-PCR are shown in **Supplement Table 1**. The PCR amplification system contained 1 µg cDNA, 10 µl 2× SYBR mix, 1 µl forward primer (10 µM), 1 µl reverse primer (10 µM), and ddH_2_O was added to make a total volume of 20 µl. The conditions for the qRT-PCR reactions were as follows: pre-denaturation at 95°C for 2 min, followed by 40 cycles of 95°C for 10 s, 60°C for 10 s, and 72°C for 10 s. Next, 1 cycle of 95°C for 1 min, 65°C for 30 s, and 95°C for 1 s was conducted to obtain the melting curve, which was used to verify the specificity of the qPCR primers. The reactions were repeated for three times for each sample, using sterilized ddH_2_O as the blank control. The Cq values were obtained after the reactions were completed. 2^−ΔΔ^Ct method was used to calculate the relative expression of *LvVgs* at different developmental stages and different molt cycle stages.

### Bioinformatic Analysis of the Vitellogenin Gene Family

*Vg* and *Vg* related transcripts were identified from the transcriptome assembly mentioned above, and their sequences were further validated by molecular cloning techniques. For sequence comparison of these transcript, BLAST search analysis was performed initially to determine if they were homologous to *Vg* gene of a specific crustacean species. Multiple sequence alignment was performed by CLUSTALW (https://www.genome.jp/tools-bin/clustalw); the amino acid translation was performed using http://molbiol.ru/eng/scripts/01_13.html; and Expasy MW/pI tool (http://web.expasy.org/compute_pi/) was used to obtain theoretical molecular weight and isoelectric point. National Center for Biotechnology Information open reading frame (ORF) finder (https://www.ncbi.nlm.nih.gov/orffinder/) was used to predict the ORF. SignaIP 5 Server (http://www.cbs.dtu.dk/services/SignalP/) was used to identify the signal peptides, NetPhos 3.1 Server (http://www.cbs.dtu.dk/services/NetPhos/) was used to predict the potential phosphorylation sites, and NetNGlyc 1.0 (http://www.cbs.dtu.dk/services/NetNGlyc/) and NetOGlyc 4.0 Server (http://www.cbs.dtu.dk/services/NetOGlyc/) were used to predict the N- and O-linked glycosylation sites. BLASTP search (https://blast.ncbi.nlm.nih.gov/) was used for the homology comparisons of *LvVgs* sequences with other crustaceans. The Fast Tree software was adopted to construct the phylogenetic tree, using the Neighbor-Joining method. The bootstrap test was used for statistical analysis of each branch, with a repetition time of 1,000.

### Neuroendocrine Factors Regulating the Expression of *Vg* Genes

Eyestalk optic nerve and other neuronal tissues have been implicated to consist of factors that can stimulate or inhibit vitellogenesis. To investigate the endocrine factors from various neuronal tissues that may differentially regulate the three *Vg* genes, an *in vitro* explants culture assay was conducted. Hepatopancreas and ovary fragments were incubated with either optic nerve of the eyestalk, brain, or thoracic ganglion. To optimize the study, we used shrimp that were at the early to middle stages (GSI 3–4%; N = 5) of gonad maturation for the test. Ovary and hepatopancreas were dissected and cut into fragments of ≤8 mm^3^. They were placed together in a well of the culture plate containing 2 ml of nutrient Medium 199 (Sigma, St. Louis, MO, USA). The dissected brain, eyestalk optic nerve, and thoracic ganglion were placed separately into different wells that consisted of the hepatopancreas and ovary fragments as described above. The culture plates were incubated in a gentle rocking/shaking device for 4 h. At the end of the experiment, all the tissues in the well were harvested and extracted for total RNA. The total RNAs were reverse transcribed to cDNA and used for RT-PCR detection of *LvVg1*, *LvVg2*, and *LvVg3* expression.

### Statistical Analysis

Relative gene expression was recorded in expression study and statistical analysis of the gene expression with mean normalized ratios ( ± SD) between the copy number of target genes and the mean copy number of the reference genes. All values were analyzed using one-way analysis of variance (ANOVA), and then the Turkey (B) multiple-range test was used for comparisons in SPSS statistical package version 19.0 (SPSS Inc., Chicago, IL, USA).

## Results

### Transcriptomic and Molecular Identification of Vg Gene Family

**Figure 2:**
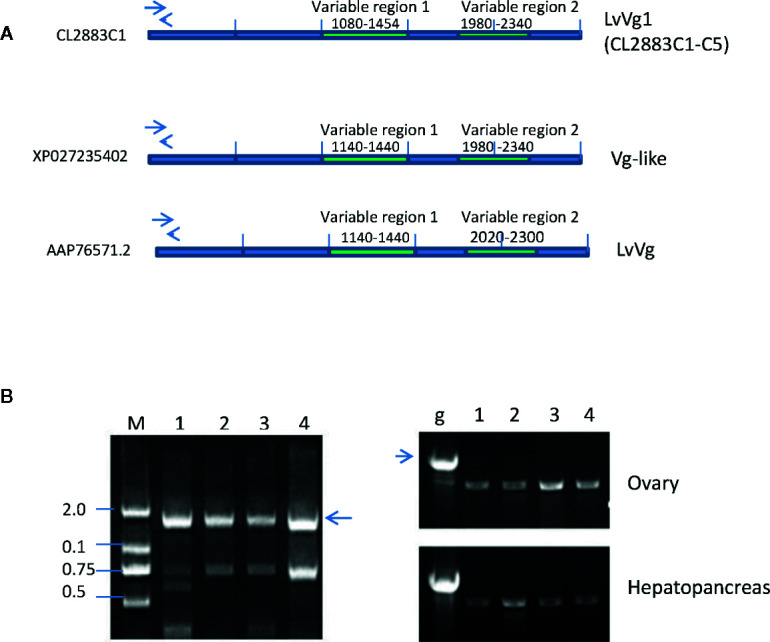
Comparison and confirmation of *LvVg1* genes isolated from the transcriptomic study with previously reported *Vg* (Genbank# XP027235402) and *Vg*-like (GenBank# AAP76571) genes in the whiteleg shrimp *L. vannamei*. **(A)** The LvVg1 transcripts (i.e.CL2283C1-C4) shared high degree of amino acid sequence identity (>97%) in the Vitellogenin-N domain, DUF1943, and VWB domain but only low degree of identity (50–56%) in the VR1 and VR2 regions. Arrows indicate location of the primers used to distinguish different *LvVg1* isoforms. **(B)** Left panel: Genomic PCR detection of multiple *LvVg1* genes from hepatopancreas derived gDNA of a single female. Lane M: 2 kb size marker; lanes 1–4 are gDNA amplified by primers specific to *LvVg1a*, *LvVg1b*, *LvVg1c*, and *LvVg1d*, respectively. The arrow indicates the expected genomic DNA amplified by the specific primers. Right panel: RT-PCR amplification of ovary and hepatopancreas cDNA. Lane g shows the PCR result of the genomic DNA (arrow) using one of the primer-pairs mentioned above. Lanes 1–4 are RT-PCR result using the corresponding four pairs of gene specific primers. All transcripts (C1 to C4) can be detected in the ovary and hepatopancreas, confirming that at least four copies of *LvVg1* genes are present in the shrimp genome.

A total of 15 transcripts encoding for full-length or partial *Vg* or *Vg*-*like* cDNAs were identified from the hepatopancreas and ovary transcriptomes of the whiteleg shrimp *L. vannamei*. Most of the larger transcripts were full-length cDNAs encoding for Vg proteins and the smaller transcripts encoded for partial and truncated cDNA of *Vgs*. We focused on analysis of the larger transcripts. These transcripts can be divided into three groups based on the amino acid sequence homology, the sizes of the deduced proteins, alignment results, and their homologies with other vitellogenins from the GenBank BLASTX search results. The first group of vitellogenin consisted of five transcripts (i.e. CL2883C1-C5) that we named as *LvVg1a* to *LvVg1e* (**Figure 2A**, **Supplement Figure 1**). BLASTX search analysis of these *LvVgs* revealed that they shared high sequence identity (i.e. 99.1–99.4%) with the previously reported *Vg* gene (Genbank# XP027235402.1), followed by the *Vg-like* gene (Genbank# AAP76571.2, 93.8%) of whiteleg shrimp *L. vannamei*. All the five *Vg* sequences shared high sequence identity in the LPN domain, DFU1934 domain, and von Willebrand domain (i.e. >99.7% amino acid sequence identity). However, relatively low sequence identity was observed in two regions that we called variable regions 1 (62% aa identity) and variable region 2 (65.4% aa identity). Since the transcriptomes were constructed using cDNAs from more than one shrimp and the presence of seven transcripts (together with the previously published *LvVgs*) of *LvVg1* suggested that multiple *LvVg1* isoforms may exist in the whiteleg shrimp *L. vannamei*. Therefore, to demonstrate the existence and expression of multiple *LvVg1* isoforms, genomic DNA PCR and RT-PCR approaches were used to confirm the existence of multiple *LvVg1* genes in a single whiteleg shrimp *L. vannamei* (**Figure 2B**). When genomic PCR was performed using specific primers for *LvVg1a*-*LvVg1e* genes spanning the first and second exon of *LvVg1*, positive amplifications were obtained **(** **Supplementary Table 1** **)**. Also, the RT-PCR results confirmed the presence of multiple isoforms of *LvVg1* (**Figure 2B** **).** Therefore, these highly similar *LvVg1* cDNAs were derived from different genes of a single shrimp. When *LvVg1a* was compared with *Vgs* of other penaeidaes, it shared high sequence identity with *Vg* of the black tiger shrimp *P. monodon* (82%), the banana shrimp *F. merguiensis* (83%), and the fleshy shrimp *Fenneropenaeus chinensis* (84%). When other *LvVg* isoforms were used for the BLASTX search comparison, similar sequence identity results were observed (data not shown).

**Figure 3:**
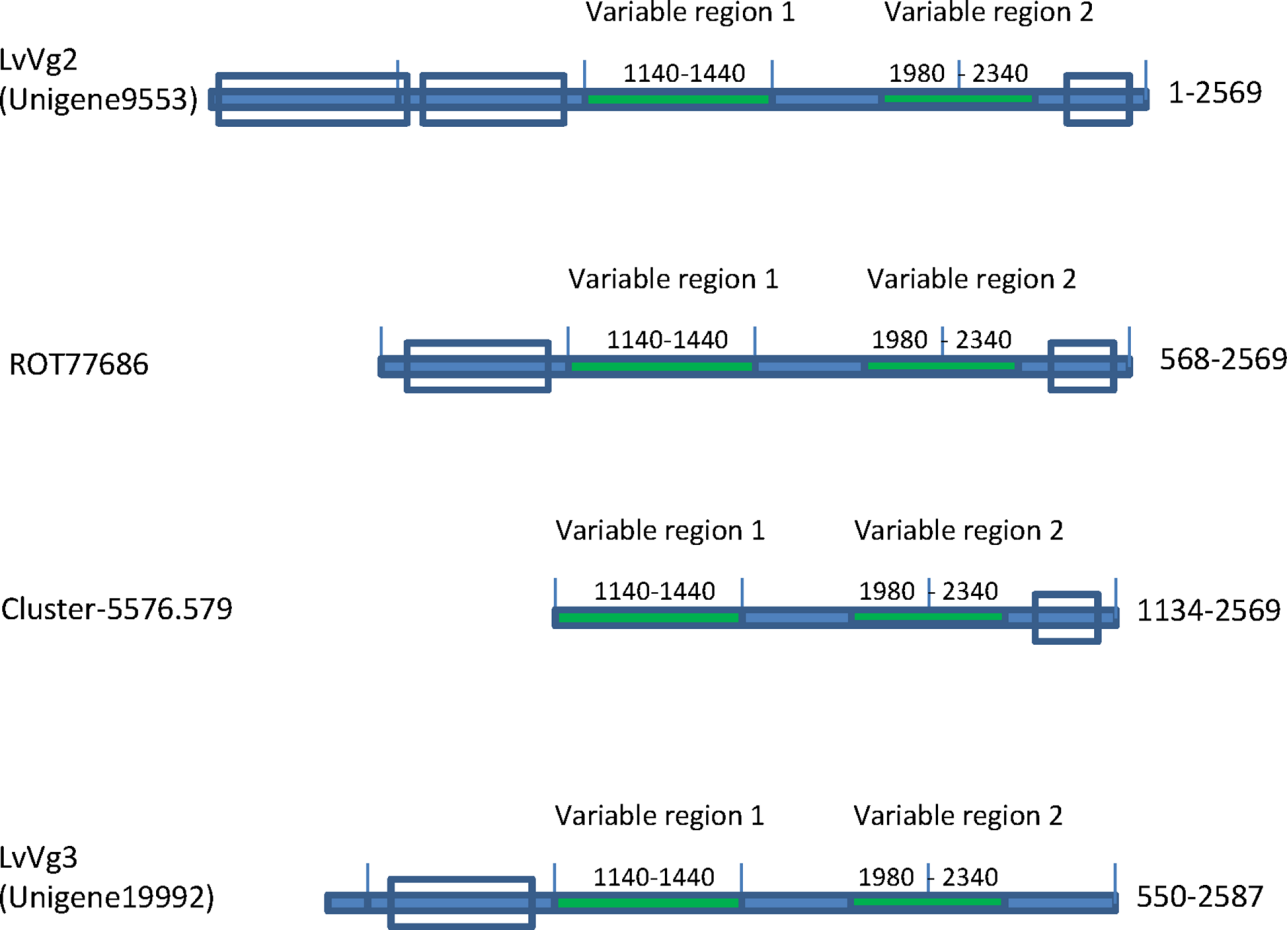
Presence of two other *Vg* transcripts (i.e. *LvVg2* and *LvVg3*) in the whiteleg shrimp *L. vannamei.* Domain structures of *LvVg2* and *LvVg3* isolated from the transcriptome are shown. Unigene 9553 was most similar (>99% aa identity) to the deduced protein from the *L. vannamei* genome project (ROT77686), followed by *MeVg2* of the sand shrimp *M. ensis* (XP027235402) (i.e. 56% aa identity) and another reported *Vtg* (Genbank# AAP76571). Cluster-5576.579 was a transcript identified from the hepatopancreas of *Vibrio hervyei* challenged *L. vannamei.* Unigene19992 was a partial transcript for *LvVg3* as it encoded a truncated *Vg* with coding sequence from AA550-2587 as compared to *LvVg1*.

The second group of vitellogenins was represented by the transcript Unigene9553 (**Figure 3**). The deduced protein of this transcript (i.e *LvVg2*) consisted of only 2,560 amino acid residues. However, BLASTX search analysis did not return any homologous mRNA sequence from *L. vannamei.* Instead, two genomic sequences were identified to share high sequence identity with Unigne9553 (see text described below). The top decapod sequence that showed high sequence identity with *LvVg2* was the *MeVg2* gene of the sand shrimp *Metapenaeus ensis*, with 63% aa sequence identity. Other penaeidae *Vgs*, however, shared only ~50% identity. For example, *LvVg2* shared only 52% aa sequence identity with the *Vg* of the kuruma shrimp *Penaeus japonicus* (BAD98732), the fleshy shrimp *Penaeus chinensis* (ABC86571), and the banana shrimp *F. merguiensis* (#Q6RG02). However, a transcript (i.e. ROT77685) identified from the whiteleg shrimp *L. vannamei* genome project shared the highest sequence identity (i.e. 97%)

The third group of *LvVgs* consisted of the transcript Unigene19992 (**Figure 3**). Unlike the other two transcript groups, the deduced protein of Unigene19992 (*LvVg3*) encoded for a smaller *Vg* like protein with only 2,087 amino acids. Furthermore, *LvVg3* appeared to be a truncated Vg that lacked the signal peptide and the N-terminal LPD domains as present in the *LvVg1* and *LvVg2*. BLASTX search analysis of *LvVg3* revealed that it was most similar to the Vg of *P. japonicus* (Genbank # BAB01568) with only 37% aa sequence identity in the overlapping region.

### Bioinformatic Analysis of *LvVg* Gene Family

**Figure 4:**
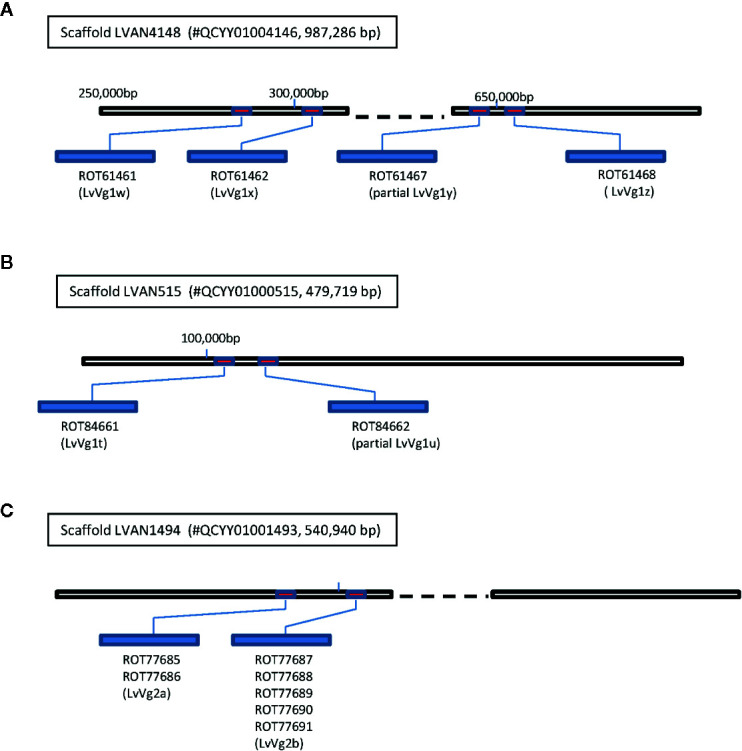
Clustering of vitellogenin/vitellogenin-like genes in the whiteleg shrimp *L. vannamei* genome. Three *Vg* gene clusters were identified from the *L. vannamei* genome sequencing project reported by Xiang’s group (22). The draft genome sequence was retrieved from GenBank database with an accession number PRJNA438564 (https://www.ncbi.nlm.nih.gov/bioproject/PRJNA438564). **(A)** Four *LvVg1* related genes (red boxes) were identified from Scaffold LVAN4148 (#QCYY010004146); **(B)** The Scaffold LVAN515 (#QCYY01000515) consisted of another two *LvVg1* related genes; **(C)** The Scaffold LVAN1494 (#QCYY01001493) consisted of a cluster of two *LvVg2* genes (red boxes). The deduced *Vgs* from these genes are shown in the blue elongated bars with GenBank accession numbers shown below the bars.

The recent release of genome information of the whiteleg shrimp *L. vannamei* allows us to search for the vitellogenin gene from the GenBank *L. vannamei* genome database. Using *LvVg* cDNAs as a query to BLAST search the *L. vannamei* genome, several *LvVg* related genes were identified (**Figure 4**). The *LvVg1* sequence shared high sequence identity to a portion of the *Penaeus vannamei* Kehai breed No.1 LVANscaffold_4148, whole genome shotgun sequence (GenBank#: QCYY01004146.1) (**Figure 4**). Within this scaffold, four *LvVg1* like genes were identified (since these genes may not be identical to *LvVg1a–LvVg1d*, we tentatively named them as *LvVg1w*, *LvVg1x*, *LvVg1y*, and *LvVg1z*). These four *Vg* genes showed a high degree of amino acid identity (>99% aa identity) with the transcripts (CL2883C1-C5) obtained from this study. However, only *LvVg1w* and *LvVg1z* isoforms were full-length sequences. These two genes consisted of 15 exons interrupted by 14 introns and they all showed the same orientation. The intergenic region between the *LvVg1a* and *LvVg1b* genes was approximately 3 kb. Similarly, the intergenic region between *LvVg1c* and *LvVg1d* was also 3 kb. The intergenic region between the *LvVg1b* and *LvVg1c* was 30kb. These four genes were located at approximately 270,000–295,000, 290,000–300,000, 580,000–595,000, and 625,000–645,000 region of the scaffold (**Figure 4A**). In addition, another scaffold (i.e. Scaffold LVAN515, #QCYY01000515) also consisted of two gene sequences that shared high sequence identity with the *LvVg1* gene (**Figure 4B**). These two genes, named as *LvVg1n* and *LvVg1p*, only contained partial sequences and encoded for only a portion of the expected LvVg1 protein.

For the *LvVg2* gene, two corresponding genes were identified from an unplaced genomic scaffold of the whiteleg shrimp *L. vannamei* (i.e., ASM378908v1 LVANscaffold_1494, NCBI Reference Sequence: NW_020868836.1). The two *LvVg2* genes were located at the 260,000–340,000 and 475,000–525,000 regions of the scaffold. They were separated by an intergenic region of 13 kb and the genes were arranged in the same orientation **(** **Figure 4C**). BLASTX sequence search confirmed that the *LvVg2* gene consisted of 14 introns. Further analysis revealed that the second *LvVg2* gene was truncated and consisted of only 8 exons. As for the Unigene19992, BLASTX search did not return any gene sequence that shared significant homology from the whiteleg shrimp *L. vannamei* genome.

As the first step to study the regulation of *LvVg* genes, we analyzed the promoter regions of these genes. We have retrieved the ~2 kb upstream region of the *LvVg1w*, *LvVg1x*, *LvVg2a*, and *LvVg2b* genes from the GenBank. The alignment was performed and the result indicated that promoter regions of *LvVg1w*/*LvVg1x* or *LvVg2a*/*LvVg2b* shared >80% sequence identity. Between *LvVg1* and *LvVg2* gene promoters, they shared a much lower sequence similarity except for the proximal region (i.e. <250 bp). In the more distal region of the promoter, most of the *Vg* members consisted of stretches of poly(AT)n rich region. This poly(AT)_n_ repeat was highly homologous between the *MeVg2* and *LvVg2* genes as they shared >80% nt identity in this region (**Supplement Figure 2**, Promoter comparison).

**Figure 5:**
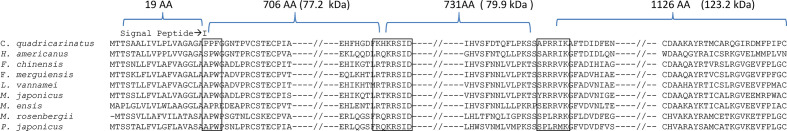
Alignment of selected decapod vitellogenin sequences. The open boxes marked regions consisting of conserved amino acid sequence for peptide cleavage sites. The symbol “—//—” indicates omission of other regions for simplification of the alignment. With reference to *L. vannamei*, the *LvVg* precursor was predicted to produce subunits of 72.2, 79.9, and 123.2 kDa.

Shrimp vitellogenins are synthesized as large precursor proteins and later processed into different subunits (23). SignalP 5.0 result showed that the first 18 amino acids constituted the signal peptide and all shrimp shared a conserved sequence of APW after the hydrophobic cleavage site. We have aligned some selected decapod Vg sequences and the result revealed that some conserved regions of Vg consisted of potential cleavage sites (i.e. RX(R/K)R) for the Ca^+^ dependent subtilisin-like proprotein convertases (PCs). Also, several potential PC cleavage sites (i.e. Arg-Arg, Lys-Arg) can be identified in scattered locations of the Vg precursors, and two conserved cleavage sites can be detected in the conserved region of the Vg precursors (**Figure 5**).

Amino acid sequence comparison among *LvVg1*, *LvVg2*, and *LvVg3* revealed that *LvVg1a* shared 51.8 and 37.2% similarities with *LvVg2* and *LvVg3* respectively. Furthermore, the similarity between *LvVg2* and *LvVg3* was 35.8% only (**Table 1**). In addition to the lowering of amino acid similarities in the three conserved domains, the degree of amino acid similarity also decreased in the VR1 or VR2 regions.

**Table 1:** Comparison of LvVg1, LvVg2, LvVg3, and other Vg homologs.

		LvVg1(CL2388)	LvVg2(Unigene9553)	LvVg3(Unigene15559)
Comparison of LvVg1, LvVg2 and LvVg3	LvVg1(CL2388)		**71.2**	**58.2**
	LvVg2(Unigene9553)	51.8		**56.9**
	LvVg3(Unigene15559)	37.2	35.8	
		Shrimp	Lobster	Crab
Comparison of LvVgs and Vgs from other species	LvVg1(CL2388)	84.2-87.3	40.1-43.2	35.1-36.6
	LvVg2(Unigene9553)	52.0-62.2	39.1-39.6	35.2-36.4
	LvVg3(Unigene15559)	36.1-37.3	31.9-33.1	31.9-33.2

LvVg1 shared a higher level of similarity or identify with LvVg2 than LvVg3. LvVg1 showed the highest degree of sequence conservation in different groups of crustaceans. The three Vg transcripts of L. vannamei were generally more similar to Vg homologs from shrimp compared with those from lobsters and crabs. Bold numbers in the table indicate sequence similarity (%) while other numbers stand for sequence identity (%).

**Figure 6:**
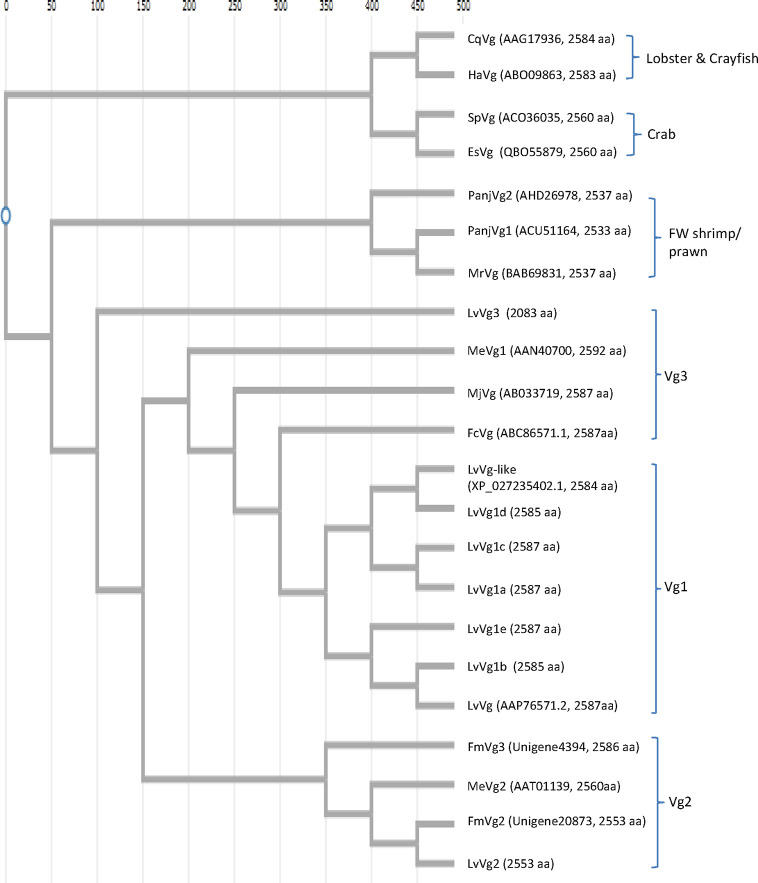
Phylogenetic analysis of selected *Vg* genes from different decapods. Nucleotide sequences were aligned first with ClustalW software (https://www.genome.jp/tools-bin/clustalw). Phylogenetic tree reconstructions were performed using the function “build” of ETE3 v3.1.1 as implemented on the GenomeNet (https://www.genome.jp/tools/ete/). The tree was constructed using fast tree program with slow NNI and MLACC = 3 (24). The GenBank accession numbers and full-length amino acid sizes of the *Vg* are shown. These include: the red claw crayfish *Cherax quadricarinatus* *CqVg* (#AAG17936.1), the American lobster *Homarus americanus* *HaVg* (ABO09863.1), the mud crab *Scylla paramomosian* *SpVg* (ACO36035), and Chinese mitten crab *Eriocheir sinensis* *EsVg* (QBO55879.1); the freshwater shrimp *Pandalopsis japonica* *PanjVg1* (AHD26978) and *PanjVg2* (ACU511645), the giant river prawn *Macrobrachium rosenbergii MrVg* (BAB69831); the whiteleg shrimp *Litopenaeus vannamei* *LvVg*-like (XP_027235402) and *LvVg* (AAP76571), the sand shrimp *Metapenaeus ensis* *MeVg1* (AAN40700) and *MeVg2* (AAT01139), the kuruma shrimp *Marsupenaeus japonicus MjVg* (ABC33719), the Chinese shrimp *Fenneropenaeus chinensis* *FcVg* (ABC86571), and the banana shrimp *Fenneropenaeus merguiensis* *FmVg3* and *FmVg2* (Unigene4394 and Unigene20873 in our unpublished data). Other *L. vannamei* *Vg* genes identified from the current study included *LvVg1a-e* (CL2883.Contig1-5), *LvVg2* (Unigene9553), and *LvVg3* (Unigene19993).

Because of the economic interest in shrimp aquaculture, more research studies have focused on *Vg* sequences of shrimp. There is a lot less *Vg* sequence information on other decapods. Of all the decapod *Vgs*, the penaeid *Vg* sequences are most reported. The phylogenetic tree revealed that decapod *Vgs* can be divided into three major clusters according to their taxonomic divisions (**Figure 6**). The first group consisted of freshwater prawns such as *M. rosenbergii*, while the second group was represented by marine penaeid shrimp and the third group was formed by crabs and lobsters. *Vg* from the freshwater prawns shared a common ancestral gene with *Vgs* of the marine shrimp and lobster/crab groups. The *LvVg1* of the whiteleg shrimp *L. vannamei* clustered together with all the *Vgs* from other marine penaeid Vgs reported. Within marine shrimp, three to four phylogenetically different subgroups can be identified. The first group consisted of *Vgs* from the kuruma shrimp *P. japonicus* and the black tiger shrimp *P. monodon*, *MeVg1* from the sand shrimp *M. ensis*, and other *LvVg1* related sequences described in this study. The second group consisted of *LvVg2*, *MeVg2* of *M. ensis* and a *Vg* from *P. monodon* (identified from the transcriptome of *P. monodon* in our different project). The third group consisted of *LvVg3*. The degree of similarity between members of the same group was very consistent. For example, all members of the Vg1 or Vg1-like group shared 80% similarity. However, members of the second group (i.e. *LvVg2* and *MeVg2*) shared only 56% aa sequence identity.

### Expression Study of *LvVg1*, *LvVg2*, and *LvVg3*

**Figure 7:**
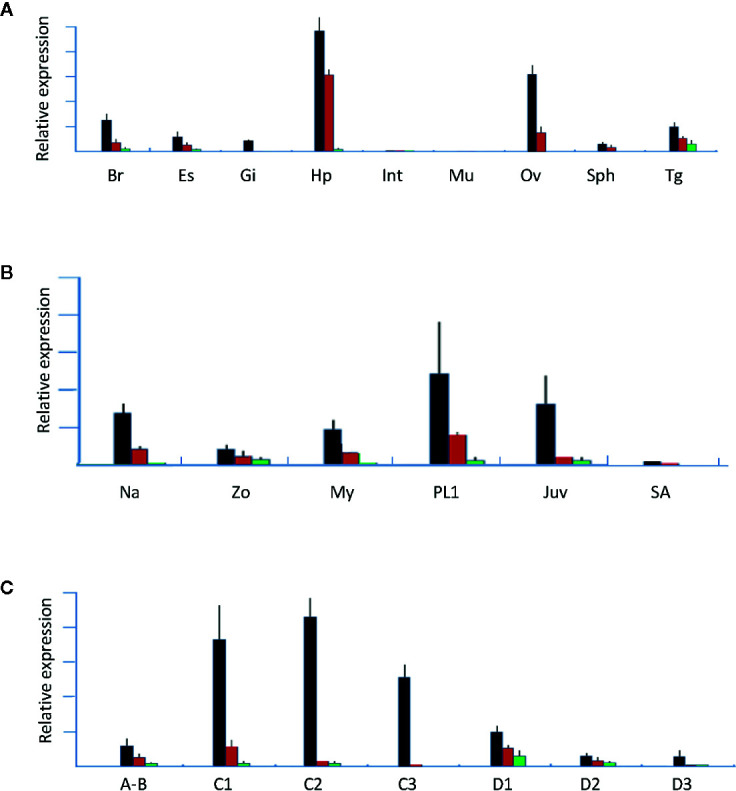
Gene expression analysis of *LvVg1*, *LvVg2*, and *LvVg3*. The black bars are for *LvVg1*, the red bars are for *LvVg2*, and the green bars are for *LvVg3* (N = 3). **(A)** Tissue specific expression of *Vgs*. Brain (Br), eyestalk (Es), gill (Gi), hepatopancreas (Hp), intestine (Int), muscle (Mu), ovary (Ov), spermatophore (Sph), and thoracic ganglion (Tg); **(B)** Expression of different *LvVg* transcripts in the nauplius (Na), zoea (Zo), mysis (My), post-larvae (PL1), juvenile (Juv), and subadult (SA); **(C)** Molt cycle expression pattern in the hepatopancreas. Postmolt (A, B); early-mid intermolt (C1–2), late intermolt (C3), early premolt (D1), middle premolt (D2), and late premolt (D3).

In this study, q-PCR primers were designed from regions that can amplify the isoforms, and tissues from females at the early vitellogenin stage were used. Tissue-specific analysis results showed that all three *LvVgs* were expressed in the hepatopancreas and ovaries with different intensities (**Figure 7A**), while *LvVg1* was the major Vg expressed in the hepatopancreas and ovary. In addition to the hepatopancreas and ovary, *Vg* transcripts can also be detected in the brain, eyestalk, and thoracic ganglion. The *LvVg1* transcript abundance in the eyestalk was low during early reproductive stages but increased rapidly during the active phase of vitellogenesis (data not shown).

At different developmental stages, *LvVgs* transcripts can be detected in the whole nauplius, zoea, and mysis (**Figure 7B**). In the hepatopancreas, these three transcripts can be detected at the juvenile stage, then starting to decrease and consolidating at a relatively low level at the sub-adult stage. At different molt cycle stages, *LvVg1*, *LvVg2*, and *LvVg3* can be detected with the maximum level at the intermolt stage (**Figure 7C**).

**Figure 8:**
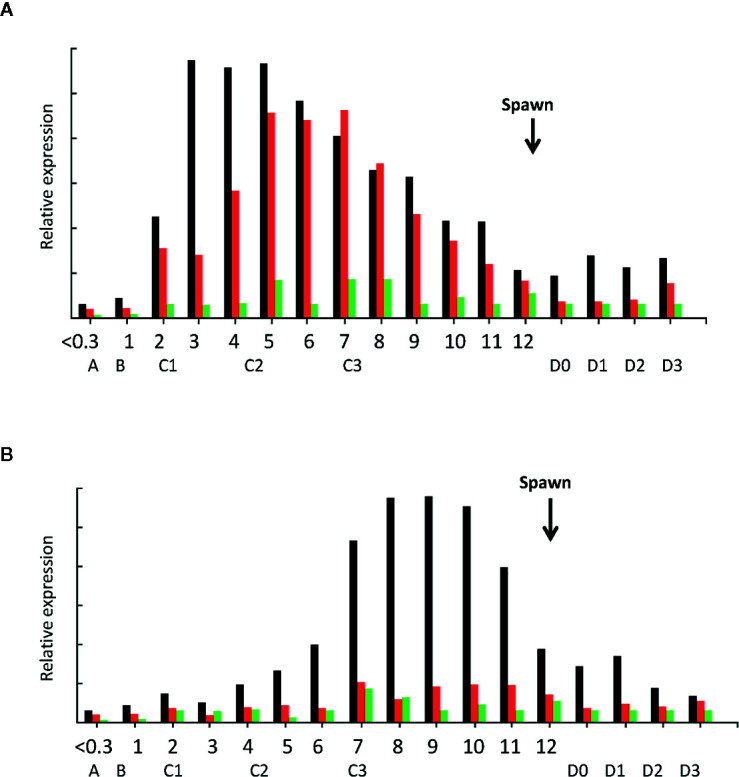
Expression analysis of *LvVg1*, *LvVg2*, and *LvVg3* in the hepatopancreas **(A)** and ovary **(B)** during female maturation cycle. The black bars are for *LvVg1*, the red bars are for *LvVg2*, and the green bars are for *LvVg3*. The gonadosomatic index (GSI) was from 0 to 12%; the molt cycle stage was from A to D3. The arrow indicates the time of immediate post-spawn. Postmolt (A, B), early-Mid intermolt (C1–2), late intermolt (C3), early premolt (D0–1), middle premolt (D2), and late premolt (D3).

At different stages of female ovary maturation (GSI = 0 to >10), a unique pattern of *LvVg* expression in the hepatopancreas was observed (**Figure 8**). In the hepatopancreas, vitellogenesis begins as the shrimp enters from early postmolt to the early intermolt stage. The expression level of *LvVg1* increased rapidly at the early intermolt stage. The expression rate maintained a steady increase towards the mid-intermolt stage. Thereafter, the expression rate decreased towards the early premolt stage. The expression of *LvVg2* lagged behind *LvVg1* and increased to the maximum towards the mid intermolt stage. Expression levels of both *LvVg1* and *LvVg2* increased steadily towards the end of intermolt. For *LvVg3*, its expression was initiated at the end of the maturation cycle (i.e. from GSI = 8–10, **Figure 8A**). For the ovary, a progressive increase in expression of *LvVg1* occurred from GSI = 0.3 to the maximum level towards GSI = 10%. The expression level decreased rapidly after spawning. At the end of the intermolt, the GSI of the females reached 9–10% and the shrimp is ready to spawn (**Figure 8B**). Spawning usually occurs during the late intermolt when the GSI is >9%. During the post-spawn phase, the GSI of the female dropped to 2–3% and a low level of *LvVg1* transcript can still be detected in the hepatopancreas.

**Figure 9:**
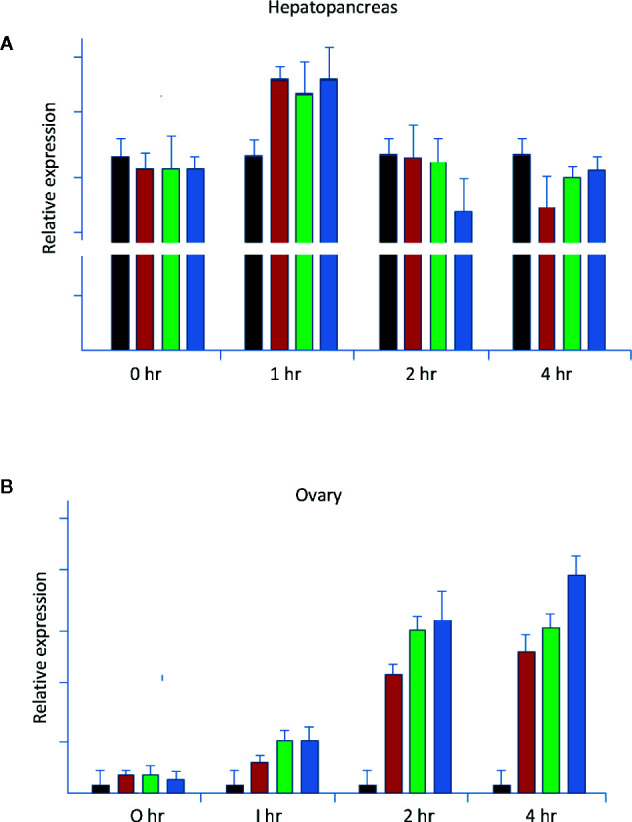
Neuroendocrine regulation of *LvVg1* expression in *in vitro* cultured hepatopancreas and ovary (N = 5). The tissues were incubated with explants of the eyestalk optic nerve (Es; red bar), brain (Br; green bar), or thoracic ganglion (Tg; blue bar). Hepatopancreas **(A)** and ovary fragments **(B)** were incubated in a medium containing one of the above tissues. The control tissues (black bar) were incubated without addition of the nervous tissues. Samples were taken at the 0, 1, 2, and 4 h time points following incubation. Q-PCR was performed to determine expression levels of the *LvVg1* gene.

It was evident that the ovary and hepatopancreas appeared to respond very differently to the treatment of the nervous tissues. For example, the hepatopancreas fragments all responded to the stimulating factors in the optic nerve, brain, and thoracic ganglion to different degrees. Compared to the ovary, the hepatopancreas fragment was highly active with the *LvVg1* transcript levels at least five times higher than that of the ovary fragments (**Figure 9**). The response of the ovary, however, appeared to lag behind the hepatopancreas as most of the stimulation occurred at 3 to 4 h after incubation. Moreover, the effect of the thoracic ganglion or brain on *LvVg1* induction in the ovary fragments was much larger than that of the eyestalk optic nerve. Compared with *LvVg1*, *LvVg2*, and *LvVg3* exhibited quite variable expression profiles (data not shown) following incubation with the nervous tissues, and no specific patterns can be discerned. In summary, the results indicated that the eyestalk, brain, and thoracic ganglion consisted of factors that can stimulate the expression of *LvVg1* gene (**Figure 9**) and the responses of hepatopancreas and ovary to the neuronal factors were different.

## Discussion

Next generation sequencing (NGS) methods including the RNA-Seq technique have provided us important tools for gene discovery, gene structure analysis, gene evolution research, and population genetic studies (25, 26). RNA-seq and transcriptome analysis is also important for gene expression regulation studies (25). In recent years, the number of vitellogenin genes identified from decapod species increases with the growth in data from genome sequencing, transcriptomic and proteomic projects (26). In this study, we have identified several *Vg* transcripts from a transcriptome assembly of *L. vannamei*. Sequences of these assembled *Vg* transcripts were validated with molecular cloning techniques. Expression patterns of these genes in different tissues, at different ovarian developmental stages, or during the molting cycle were examined. Potential regulation of the *Vg* genes by major nervous organs was also investigated. Moreover, bioinformatic approaches were also utilized to dissect the phylogenetic relationships of *Vg* homologues, and data-mine information about *Vg* genomic sequences of *L. vannamei* from the public database.

In this study, we identified many Vg or Vg-like transcripts from the transcriptome of the whiteleg shrimp *L. vannamei.* These Vg-like transcripts can be divided into three major groups with the *LvVg1* being the most abundant type. The first report for the existence of multiple *Vg* genes in marine shrimp was in the sand shrimp *M. ensis*. In the study, genomic southern blot analysis using a probe spanning a large *MeVg1* cDNA had identified many DNA fragments hybridized to the probe (2). It was proposed that at least three to four Vg genes were present in the genome. Subsequently, a vitellogenin like cDNA (i.e. *MeVg2*) was cloned and characterized. The *MeVg2* gene shared only a 50% amino acid sequence identity with the *MeVg1* gene. The presence of multiple vitellogenin genes was also reported in the freshwater prawn *M. rosenbergii* (10). In our analysis of transcriptomes from the hepatopancreas and ovary of the banana shrimp *F. merguiensis*, the black tiger shrimp *P. monodon*, and the lobster *Panilus homarus*, multiple vitellogenin transcripts have also been identified (data not shown, but available upon request). Therefore, we have concluded that multiple vitellogenin genes also exist in most decapod crustaceans. Concerning the different *LvVg* transcripts identified in the whiteleg shrimp *L. vannamei*, the *LvVg1* transcript constituted the major RNA species and further study demonstrated that four to five *LvVg1* isoforms (ortholog) existed. The presence of multiple isoforms of *LvVg1* suggested the need for production of a large amount of protein for rapid ovary maturation. The production of multiple vitellogenin gene isoforms has been reported in many fish species (25–27). Since shrimp produces a nutrient poor egg (i.e. oligolecithal), we speculate that the evolution of multiple *Vg1* genes also occurred in other decapods for the production of a much larger content of yolks.

*LvVg2* was the second most abundant vitellogenin gene member identified from the whiteleg shrimp *L. vannamei*. Genbank BLASTP search analysis has identified two *LvVg2* gene sequences (>99.8% amino acid identity) from the published *L. vannamei* genome (**Figure 4C**). It was most similar to the *MeVg2* gene of *M. ensis*, sharing 52% amino acid sequence identity. The full-length coding/amino acid sequence of *LvVg2* was relatively short (i.e. 2,560 aa) which is similar to the *Vg2* identified in *M. ensis* (i.e. 2,553 amino acid). In the hepatopancreas and ovary transcriptomic dataset of the shrimp *F. merguiensis*, a second form of *Vg2* (i.e. *FmVg2*) also existed and it shared the highest sequence homology with *LvVg2* (data not shown but can be provided upon request). *FmVg2* was a shorter *Vg* sequence similar to that of the whiteleg shrimp *L. vannamei* and the sand shrimp *M. ensis*. Therefore, we believe that homologous genes for *Vg2* also exist in other shrimp and the size of these *Vg* genes is also shorter. Despite the claim that a second *Vg* gene was present in the mud crab *Scylla* *paramamosain*, closer inspection of the two sequences indicated that these two genes can be considered to be *Vg* isoforms derived from two different genes (19). However, in our crab hepatopancreas and eyestalk transcriptomic database (unpublished), we have identified a second *Vg* like gene in the mitten crab *Eriocheir sinensis*. Sequence alignment of the transcriptomic database derived *EsVg2* with *EsVg1* from the published data revealed that they only shared 54% similarity to each other. Moreover, full length sequence of *EsVg2* was much shorter than that of *EsVg1* (2,560 *vs.* 2,525). Although *Vg* sequence information in crabs is far from complete as compared to shrimp, it is logical to speculate that crabs also consist of multiple vitellogenin genes. Based on the above information, we speculate that other forms of the *Vg* gene, such as *Vg2* (shorter in length) and *Vg3*, may also occur in other decapods including lobsters and crayfish. In other words, the presence of multiple vitellogenin genes may universally occur during the early evolution of decapods.

Understanding the structure and organization of *Vg* genes may provide information for the evolution of this gene family and regulation of gene expression. We have previously investigated the *Vg* gene structure of shrimp (2), crab (9), and lobster (28) and the results indicated that the organization of *Vg* in decapods was highly conserved. Most of the decapod *Vg* genes consist of 15 exons interrupted by 14 introns (4). In the sand shrimp *M. ensis*, the *MeVg2* consists of 13 exons because of the fusion of exons 6–7 and 7–8 (3). Here, from bioinformatic analysis, it was found that all the full-length *LvVg* genes identified consisted of 15 exons interrupted by14 introns and the exon-intron junction were also conserved in all the genes. In the red crab *Charybdis feriatus*, the *CfVg1* gene also consists of 14 introns (9). Although the mud crab ***S.*** *paramamosain* has a second *Vg* that consists of 11 introns, closer inspection of this *SpVg2* gene revealed that it was most likely a partial *SpVg1* gene. The situation is similar to the truncated *LvVg* gene discussed in the *L. vannamei* genome study above. Therefore, more sequence information is needed to confirm the conservation or divergence of *Vg* gene organization in decapods. Since the proximal promoter region of the *LvVg1* and *LvVg2* genes shared a significantly high degree of identity, the basic regulation mechanism of *Vg* may be conserved in shrimp.

Many genes are known to be arranged along the chromosomes in groups of related gene. These groups are called gene clusters. Related genes may be arranged in more than one physical cluster and a whole set of related genes is called a gene family. This is the first report for the vitellogenin gene family in shrimp and multiple *Vgs* exist as a cluster in the genome. It is common for the evolution of multiple copies of highly expressed gene. For example, the CHH/MIH/GIH gene family is also known to be arranged in clusters. In *M. ensis*, the CHH/MIH/GIH gene family represents an important group of neuropeptide hormones for growth and reproduction control. Members of the same family may have different gene functions. Two clusters of CHH-family neuropeptides have been identified. In many fish, several vitellogenin genes have been identified and the vitellogenin gene family members are also arranged in clusters. Gene clusters and gene families vary in importance in different taxonomic groups. In *L. vannamei*, three different Vg gene clusters were identified similar to the CHH family clusters, but it is still unknown whether these *Vg* genes in *L. vannamei* are located on the same chromosome.

There are many examples for *Vg* gene duplication and gene clustering in other vertebrates (27, 29). Although it is not known whether the three clusters of *Vg* genes identified in the whiteleg shrimp *L. vannamei* are linked, we have proposed an evolution model for the formation of the *LvVg* gene family based on the transcriptomic sequences and available genomic data from public database. In this model, *LvVg1* and *LvVg2* genes were derived from a common ancestor gene. After the separation from *LvVg1*, only a few mutations occurred in *LvVg2* and a more recent gene duplication event occurred to produce the two highly similar *LvVg2a* and *LvVg2b* genes. *LvVg3* was derived from the same lineage as *LvVg1* at a later time but remained relative stable throughout evolution. However, ancestor of *LvVg1* may undergo two or more rounds of gene duplications and give rise to the current four to five *LvVg1* isoforms.

The lack of complete sequence information for all *Vgs* is the major reason for the discrepancy in identifying the major sites of vitellogenin synthesis reported in different crustaceans (30). This discrepancy may be a result of using primers that are not common to amplify other vitellogenin gene members in PCR. Therefore, to obtain a precise spatial and temporal expression profile of different *Vg* genes, gene specific primers must be employed in RT-PCR or qPCR assay. Because of the lack of additional *Vg* gene sequences, results reported in many previous studies in decapod may not be accurate (16).

In *L. vannamei*, it was confirmed that both the hepatopancreas and ovary are the major synthetic sites of vitellogenins and vitellogenin expression follows a bi-phasic expression pattern for the completion of vitellogenesis in shrimp. The hepatopancreas is the major synthesis site in the initial phase and ovary will become the major site at the later stage of vitellogenesis. The differential expression patterns of the three vitellogenin genes during ovary development further indicate their functional diversification, which merits further in-depth studies. It is also obvious that the amino acid profiles of these *Vgs* are different as they may fulfill different functions at different developmental stages.

The expression of *LvVg1*, *LvVg2*, and *LvVg3* at different life cycle stages were investigated. The results indicated that all these transcripts can be detected in the nauplius, zoea, mysis, postlarvae, juveniles, and subadult. In the expression study, the detection of *Vg* transcript in early larva such as the nauplius suggested that some of the transcripts could be maternal. During early stage of embryonic development, when the transcriptional process is not fully functional, the reservation of these maternal vitellogenin transcripts would be important for successful embryo development. As a nutrient molecule, vitellogenin can be processed into small peptide and amino acid. As shrimp produce a nutrient poor egg, nutrient from the maternal part can supply all energy requirement for the nauplius (28). Feeding only begins when the nauplius metamorphoses into the zoea. The presence of *Vg* transcripts in free swimming nauplius, mysis, and post-larvae suggests that *Vg* also has its function in larval development. As the larvae begins to assume active feeding from predation, the expression of *Vg* is reduced at the later juvenile and subadult stages.

As a nutrient molecule, *Vg* is first produced as a large precursor molecule and later processed into subunits in the hemolymph (23). Many biochemical studies reported the different sizes of *Vg* subunits in the hemolymph (23). This is probably due to the presence of several cleavage sites (RR, KR or RK). Cleavage at these sites may further process the *Vg* into smaller peptides of different sizes. During the transcriptomic screening of *Vg*, many smaller *Vg*-related transcripts were identified. Some of these transcripts may represent degrading gene products. However, some transcripts should represent alternative splicing products of the full-length gene or transcript from a partial vitellogenin gene. For example, the deduced transcripts for the partial *LvVg1y* and *LvVg1u* gene can produce transcripts of ROT614614 (GenBank#) and ROT84662 (GenBank#). Similarly, in the sand shrimp *M. ensis*, Northern Blot analysis revealed the probe hybridized to many mRNA of different sizes (3). Therefore, some of those transcripts may produce truncated *Vg* proteins. These small *Vg*-like transcripts can be processed to produce smaller *Vg*-like protein and supply sufficient amino acid or raw materials for growth.

In many transcriptomic datasets that we have on-hand, there are many *Vg* or *Vg*-like transcripts containing sequences with deleted N-terminal LPD domain or deleted C-terminal coding regions. These transcripts could be derived from alternative splicing of the *Vg* genes. For example, a search of the Genbank for the whiteleg shrimp *L. vannamei* genome project revealed a *Vg*-like gene encoding for a partially deleted vitellogenin (i.e. Genbank#: ROT61467). Transcription of this mRNA will result in a N-terminally truncated Vg protein without a signal peptide sequence. Therefore, these truncated *Vg*-like molecules may not be a secreted product but will only function in an intracellular manner. In the mud crab *S.* *paramamosain*, a second vitellogenin gene has been cloned, and the expected cDNA of this *Vg* gene also lacks the signal peptide and is therefore truncated (19).

Vitellogenesis in decapods is known to be controlled by many hormones (31). In shrimp aquaculture, unilateral eyestalk ablation is widely used to induce female gonad maturation, as eyestalk ablation removes the source of the gonad inhibiting hormone (GIH) which is a member of the CHH/MIH/GIH family neuropeptides (32). Meanwhile, there are many reports for the presence of a gonad stimulating hormone (GSH) in the brain, thoracic ganglion, or other neuronal tissues in crustacean, but information on the identity of this GSH is scared. In the crab *Potamon koolooenseis*, extract of thoracic ganglion induced oocyte growth and precocious vitellogenesis with an increase in the ovarian weight or gonad index and oocyte diameter (31). However, the brain extract injection did not produce marked changes in the immature ovary. The results suggest that the ovarian activity, particularly growth and vitellogenesis, may depend on neurosecretion of the thoracic ganglion (32–34).

In this study, when hepatopancreas and ovary fragments were co-incubated with eyestalk optic nerve and other neuronal tissues, different responses were observed for the ovary and hepatopancreas. Stimulatory effects of the three neuronal tissues on *LvVg1* expression were all detected. The hepatopancreas fragment was highly active with the *LvVg1* transcript induced to higher levels only at the 1 h time point, while response of the ovary lagged behind the hepatopancreas as most of the stimulation occurred at 3 to 4 h after incubation. Moreover, the effect of the thoracic ganglion or brain on *LvVg1* induction in the ovary fragments was much larger than that of the eyestalk optic nerve, a phenomenon not observed in the hepatopancreas fragment. More in-depth study is needed to explain the different responses and to identify the causal factors in these neuronal tissues (33).

In conclusion, this is the first comprehensive study of multiple vitellogenin genes in a decapod crustacean. The clustering of multiple vitellogenin genes in the whiteleg shrimp *L. vannamei* suggests that evolution of the *Vg* genes is the result of several gene duplication events. The results presented in this study demonstrates that two phases of vitellogenin gene expression are needed for the completion of vitellogenesis: the extra-ovarian hepatopancreas phase followed by the intra-ovarian phase undertaken by the ovary. Because the responses of hepatopancreas and ovary to eyestalk, brain or thoracic ganglion stimulation are different, vitellogenin expression in these two tissues may be under control of different endocrine factors. In addition to its nutrition-providing role in reproduction, shrimp vitellogenin genes are also likely to be involved in growth and molt cycle regulation.

## Data Availability Statement

All datasets generated for this study are included in the article/**Supplementary Material** and other “data not shown” results mentioned in the text are available upon request.

## Ethics Statement

The animal studies described in this report were conducted under the guideline of animal research ethics approved by the Committee for animal research of the Guangdong Ocean University.

## Author Contributions

Paper writing: SC, WW, BL. Data analysis: SC, WW, BL, LS, TZ. Performing experiments: TZ, CW, LS, AK, SC. Securing funding support: SC, WW. All authors contributed to the article and approved the submitted version.

## Funding

This research was funded by the National Natural Science Foundation of China (#31572606), the Natural Science Foundation of Guangdong Province (#2018A030310049), Guangdong Provisional Research Grant (#2014B020202014), GDOU’s Enhancement Innovation Project (#2013050501, #2013050210, #2013050109), and Nanhai Scholar Project of GDOU.

## Conflict of Interest

The authors declare that the research was conducted in the absence of any commercial or financial relationships that could be construed as a potential conflict of interest.
