# Supplementary material for: Investigation of Gene Sequence Divergence, Expression Dynamics, and Endocrine Regulation of the Vitellogenin Gene Family in the Whiteleg Shrimp Litopenaeus vannamei
Source: Front Endocrinol (Lausanne). 2020 Nov 19;11:577745. doi: 10.3389/fendo.2020.577745 (PMC7711153; doi:10.3389/fendo.2020.577745)
Supplement: Supplementary file 1 [file DataSheet_1.docx]

>CL2883.Contig1_All

ACCTGCCACAAAGGCGAGAACGAGTAGTCGTGGTGAGCTCGTCCAGCCAACATGACGACCTCAACTCTCCTCTTCGTTCTCGCCTTTGTGGCAGGTTGTCTGGCAGCCCCCTGGGGAGCGGACGTGCCAAGATGCTCCACCGAATGCCCCGTCACCGGATCCCCCAAACTGGCCTACCAACCCGACAAGACCTACGCCTACCAATACTCCGGCAAGTCCAAGGTCCAGCTCAAGGGCGTGGACAACGGCGACTCGGAGACCGAGTGGACGGCACAAGTTGATCTCACCTGGATCAGCCCTTGCGACATGGCCATCTCCTTCAAGAATACCAAGGTGGATGGCACCCCCGGTCCCATCGTTGCCAGGACGCTGGAGAGACATCCACTGGTGGTGGCCGTCGTCGACGGAAGGGTGCAGCACGTGTGCGCTCACCCAGAGGACGAACCATGGGCCATCAACCTGAAAAAGGGCGTGGCTTCGGCTTTCCAGAACTCCATTCCTTCTCTGTCTGCTGTCAGCTCAGGCATCACAGTAACGGAGACTGATGTTGTGGGAAAATGCCCAACAAAGTATGAAATTGAGACCGAAGGAGAGAAAGTCATTGTCGTCAAGGAGAAGAACCACCGCCACTGTCAACAACGTTACCCAACACCCGCTCATATACCTGCACTATGGCTGAAGGCTCCCCTGCCAATCCAGGAATCCACGTCACAGTGCAAGCAGGAAATCGCCAATGGCATTTACACCGCCATCACGTGTCAGGACAAGAACATCGTTCGACCTGCCATTGGAATCTACAAGTACGTGGAGGCCAGTCAGGATTCAACACTTCGCTTCATCTCGGAGTCCTCCGACACTTCAGCTATCAGTGCCATCCCTTCAGGAGAAATGCAAGTTGAAAGCCTCCTGTACAACCACCAAACAATGAAGGACCCACAACTGGCACCTGAGCTGGATGAGCTCATGAAGGGGATCTGTGACAAGACCAAGGACACAGTTGAGGCTGAAGCTGCTGCTTTGGTTGCCAAGGCTCTCCATCTGTTACGTCGTGTTCCAGAGACAGTTGTGGTGGAGACTGCACAGAAAGTGAGACAAGGACATTACTGCAGTGACTCTGCCAGGCTGGAGAGTATCTTCTTGGACGCAGTTGCTTTCCTGCATGAGTCTGGTGCAGTAAAGGTCATGGTCCAAGAAATCGAGAATGGACGAGCAACAGGGGGACGTCTCGCTCTGTACACGGCAGCGCTCTACCTCATCCCACGACCCAGCATTGAGGCAGTCAAGGCTCTCACGCCACTCTTTGAAAGCCCTCGCCCAGTGCCCTCGGTGCTGCTGGCAGCTGCTTCCATGATAAACCACTACTGCCTTCATACTCCAGCTTGCCACCAGAAAGCTCCAGTGGCGAGAATTGCAGAGATTCTGGCCACCAGAGTCCAGAGTCACTGCTCTCCTTCTGCTGGTGCTGAGGGCGAGGAAGTACCCCTTGCATTCTTCAAGGCAATAGGGAATATGGGTGTAGCTACACCTGCCGTGACAAGGGCAGCCGTCCAATGCATTGAAGAAGAAGGACTGGAAACCAGCATTCGGGTAGCTGCAGCACAAGCCTTCAGACAAGCCAATTGCTTCCGTCCAGCAGTTGAAAAGCTAGTAGACATTGCCGTCCGACCAGCCTTTGACACCGAAGTCCGCATCGCTTCCTATCTGGCAGCTGTCCGATGTGCTGAACAGGAACACCTGGAGAAAATTATTGAGAAGATTTCAAAGGAAGAGAATACCCAAGTGCGTGGATTTGTTTTGGGTCACCTGATCAACATCCAAGAGGGTAGCTGCCCCAACAAAGAAAACCTCAGGTACCTCCTTGCCAACGTTGTCATCCCTACCGACTTCGAGAAGGACTTCAGGAAATTCTCTCGACATATAGATATGGCTTACTATGCCCCTGCCTTTGGCATGGGTGCCGGCCTCGAGTCGAACATCATCTATGCTCCCGGATCTTTCATTCCTCGTGCTGTTAACCTGAACATGAGAGCAACTGTGGATGAGACGCCCATGGACATAGCAGAGATTGGTGCGCGCTTTGAAGGAGTCGACTCCATCATTGAAGAGCTCTTGGGCCCACAGGGATACCTACGCAAAGCAACATTTGGAAAGATTATGGAGGACATTACGGGTTTTGCAGGAGAGAAAGGCCTCAAGATCATGGAGCACTTCAAGCACACAATGAGGACCAGGCGATCCATCGATGCTTCTGTCATCTCCGACTTCTTCGGCAAGCTGTATGGTGAGAGCAGTTCGCACACCCACGCCGATATATTCGCCCGGTTCATGGGGCACGAGATTACTTTCGCAGATGTTGCCCAAAGCCTCAAGGGTGTCACAGCTGACACACTCATTGAGACTTTCTTCTCTTTCTTCGAGAATTCCTTGGAACATATGAAGGATCTTAACCTGAACACAGCAAGAACTGCTCAGCTTTCCATGGATTACTCACTGCCCACCATTCAGGGCACACCACTCAAGCTGAACTTAGCTGCAACTGCTGTTGCTGGCCTCAAGATGGAGGGCAACGTCAACATTGGCCAGATCCTCTCTGACCTGGGCAATTCCCACACCGGCATCAAGGTGTTCCCAGGCCTTTCTGTACAAGCCACTGGTTTTGTTGGCTTTGAGTGCCGCTTTACCAAGGTGGGAATCGAGATGCAGAACACCATCTCTAGTGCCACTGGAGCCGCCATCAACATCAGAACAACTGAAAACAAGAAGATCGAGCTGGAATTGGAGATCCCTGACAAGATGGAACTCCTCAACATCAAGGCCGAGACTTACCTTGTCAAAGCTAGGGGAAAGAAGATGACTAAGATTTCTCCTTCCTCCATGAGAGATGTCAGGATTGAGCGCAGGTCCTGCATTGCTGCTTTGGAACCAGTATTTGGCCTCAAGGTGTGCTATGACATGAACTTCCCTGATGTGTTCCGTGCTAATGCCCTGCCACTTGGTGAACCAGCCATCGCCAAGCTGTACGTTGAGAAGGCAGATCCTTCCATGAGAGGTTACTTAGTGACTGCTGCCATTAAGAACAAGAGAGGCAACAAGCTCATTAAGATGAATGTAGAAGCAGCTGGTGCCTCAACACCAAGAAGAGCAGAAATGACCCTGTCCTACACCAAGGAAGAAGGAAGCCACATTGTTTCTGCCAAGCTTGATTCCTCCAGCATTGCTGCAGGAGTGTGGACTACTCTCACCAACGAGCAAGGACACAAGGCAGTAGAGACTTATGTCAACTTCAAATATGGTCAGACTGCTATTTCTCGAGGCATCAAGCTGGAAGCGATTGCAAGGGAAGGAAGCGTGGGAGAGGAATTCCAAGTGAACGTTTTCAGCAGCGGCACCAGGAGCTTCCCCTCTAAATCTCAAATTGTAGAGGCTAAATTCATCAAGAAAACTAGTGGACCTGAATTCAATGTGGATGTGATCTGCATGACCAAAAATGCTTTAGCTGATTATTTCGACTTAAACATTGAAGTTGGAGCTGATTTCATGAGATTTTCTCCTAAAGCTCTGTATTCAACAAGATACATTCCCAAGACCCGCATTTTCTTACCTGTAAACCTGCGAAAGCTAGAAATCAATGCTGCCACTGCAGCCTGGAAAGTGACGTCGTACATTCGTGAAGGAAGTCAATCTGGCGAAAGCCGTGAGTTCAGTTCTGCTTTCAAGCTTGCCAAGGGAAGGACGGATGTCATCTTTGTACAGGCTACTCATACGATTGAAGGCAGATTCCCACAAAACGTCATCATCAAAAATGTAGCAACAGCCAAAGTTGGCAGATCATCATACAGAGCAATGTATGATGTCTTCTATCACCCTGAAAAAGTGGGAGCTTCTATTGAGGTTTTGCAGGCAGCAGGTAATGAGAAGGTTGCCCAGATAGAAGCAATTTACGAAATTTCCGGAGAGAAGCACTGCGCTAAATTCTTGGCGGCCATTCCTGGCTACATTCAACCAGTTAAAGTTGAAGCCGAGATTGAACAAGAAGCAGAAGGTCGCTACGCACTGGAGGCCGCCATCAAATATGGACCACGTACAGTACTTGGAGTGAGTGGACCAGTCCTAGCTCGTTTCACCTCCAAAGCCAACAAGCTGCAAGCCAACATCAAGCTCAGGGCAATGGCAAGTGAGCCCTACATCATTGGTGCCAATGTTGTGTTTGGCAACAAGAAACAGGTGATAGCCATGGAAATCAAGGAGCGAGAAGAGCCACTCTTTGGTGTAGAATGGAAGATGGTTCAAGAGAGTTCTGAGAAGACCACTGTTGGCATAGCATTCGTTCTCCCTGCCCTTATTGAGAACAAGGTCGATGCCATTATTACTGAGGAACTTATCCATGTTAATTTTAACAACTTGGTTCTGCCTAATACTTCATCCCGCCGTCGGGTCAAGGGATTCACTGATGTCAACATTGCAGAGAAAAGGGCAAATGTGGAATTTTCTTGGGATGCCGATAATGCTCCCGAAAAGAAGTTGGTGTTGGATGCAAGTCTGATCAGCAGTCCTGCCAACCCTGGACATGCTGAGATCCACGGGAATGTCGTCATTGCCGGAGAGCCTTACCACGCCAAATTGATTCTGACTGCCACAGATCTCATAGAGCACATGGAAGGGGAAAATGGATTCAAGTTGATCCTGACAACTCCTAGCCAGAAGACGGTTGTCGTGGGAGCCTCCTGTGATGTCCAGCTGGCAGGAGCCACCACTAAAGTCATTTCCACCGTTGAATACAAGAACGTGAGGGATAGGAAATACAAATATACAAGTGTGATTGCCTTGGAGAGGCTTGGTGGTCCACTTAATTATGCCGTAGAAGCCAAGGTAACTTACAAACAACCTGGAACAGCAGAAATAAAGGTAGAAACAACAGCAAAACATCATTGGACACCAGAAGAACATGTTGTAGCATTCAAGGTGGCTGCTGAAGCTCCAGTACTGAAGACGCCTGCCATGATTGCATTCTCCATTCACAATGCACCAAACGCTTTTGTTGGAGTCTGCAAGATCGAAAGAACTGCTCCTTTCACTGCCTTTGAATGGAATGTACAGGTTACTCCTGAAGGAGGAATTGAAGCTGTTGAAGCTGGTGTGGACATGAAAGCCATCATTGAAGTTCTGAAGATTGTTCGTGCCATTGCTACTCTGGAGGAAGAGAGTTATGAAACTTATGGCCCACACACAGCTCAGTACCAGTACCGCTTCACAAGGCCATCACCCACTTCTTACACCATGCAGATGAGGACTCCAACCCGCACCATGGAAGGAAGAGCTAAACTATCACCAAGGGAATCTGGAATCAAGTTCTACCCCAATAAGGGCAAAACTGAATCCAAATACGAAATTGGATACAAGGTCAACCACGAGGGAAGGTGGGCACAACGTGCGTCCAAGTTGGAAGTCAGAATGAACCATCCAGTGCTTCCTAAACCCATCATGGCCGCTGCTCAGTACACAGTAGCTGAAGGAACAATGAGGGGAACAATTGAACTGGACATTTTCCCAGAAGAAGCCGACAAAATTACTGGAACTGTGGAAACTCAGAGAATTTCAGAAAATGCTATCAGGGCAGAAGCCTTCTTGACTGGTAGGATGTTGAAAGTGAACCCTAAAGCTATCATCACTGCTGCCTATGCACCAGAAACAGTTGCTTTGGATGTAGTGTTCCACAAGACTCCGTCTGCAGCACCAATCTTCGCCATTGCTGCCAAGTATGACAAGACTGCAGCTCACAGTGCAGCTGCCACATTGACAGTAAAGATGGAAGAGCGACCTGTCTTTGAGATGAGTGCAGTGACCGAACCCGAGGAACCAGCCACCTGCAATGGCATCAGAATGAATGCTGTTGCTTATGCAGCAGCTTTTGGAAAGTACAACGTGTTCTCCAAGATGTGCAGGCCCGCCTTCATTGAGGTGACCGCAATGCGACCTGGTGGAGCAAAGGAGTACACTGCCAAGCTTGGCCTCCGATACCCTGACGCTGCTGAAGCAGGCGTATATGTGGCGAGTGGCAGAGCTGGAGAGAGTCGCGGTGTTGCTGTTGCTGCTGTGAAGCTGGCTTCACCCACAATGCTACAGTTCGAGGTGGCTCATGAACCAGAAGAAGCACACATTGTAATGAGTGAAGTGACAAGTACCCTCAGAAAAGTCGCCATGTCTCTCGAAACAGTTGCAATGGAGGCCGTCCAGTTCCTCAAGGAAGAAGCTGCTGCAAAGGGTGTCGAGTTCCCGTCATCTCACTTTGTCAGTCTAGTGGATGAGGCGAATGAGGAAATCAAAGCCATTTACCGAGATATCGTCTCAGAGATGAGAATCCTTGACACTGAGTTGATTGCTGATATCTTGGAAAGCCCTACGGTGTCCTTCGTGTCGCGTGTCTACCTTGGAGTATGGTCACAGATTGCTCGCCTTCAACATCACTTTTCAACCAGGGCGGTTGAAATGATCCAGCAATGGCAGGAACAACTAACAGACGTTTCTGAAATCTTTATCGAAGCTGTTATGGAGATAGTGCAACTCTTGGAAGCCGGAGAAGTACCTGAAACAGTTCGTGTAATTCTGGAAAAAATTGAGAACACTGAGGTGTTCAGGATTGTAAAGAGAGAAGTGAACGCAGTGTTGGCAGAGTATCCTGAGGAGTATGAGGCCGTCAAGCACATCCTCACCAGGGTGACGGCCACTCTCAAGCACGATGCTGATATTGTGTACAAGAGGATCATGGAGACACCAGCTGTTCAAAGGATCTTTGCCTATGTTATGCAGTACATCAACTCGGAGCGCGTGTTTGCTGAGGAAGCAGGAAGTGTTGCCAGCCTCATTCTCAAAGAATTTCTTTTCGTTTCAATTGAAAGCGAAGGCAACGGCATTGCAGTCCGAATTCCCCTCCACCGACCCTTGTATTCACTGACGCAAGTGGCACAAGAAGCAGTGCCCAACCCTGTCACAATGCTCGAGAACCTGATATTTGCCTACCTTGAATACATTCCCATCCCTGTGAGCGACGCAATCTGGGCCTACTACAACTTCCTTCCACGCTACATCACGGACGCGCTGCCGCCCTACCCACGAACAGCCATGGTGGTTGGCGGCACTGAGATCCTCAGCTTCAGCGGCCTTGTTGTGCGAGCACCTCGCTCGCCCTGCAAGCTTCTCCTGGCTGCTCACGGCTCCCACCGCCTCATCATGTCCCACCCGCAAGCCTCAGCCCCGGCACAGCTTGAGCTCAAGACACCAGCAGCCACCGTGATCATCAAGCCTGACTTTGAAGTCCTGGTTAATGGCCAAGCCCTCGGGGGATCCCAGCAAACCATCGGAAACGTTAGGATTGTGAACACAGCCAAGCACATTGAGGTGGGATGTCCCCTGATGAGGGTGATCGTTGCCAAGGCAGGCGAGGCCGTAGCTGTTGAGGCTTCAGGCTGGATCTTTGGACGCGTAGCAGGGCTACTGGGCCCCAACACTGGAGAAATTGCCAATGACCGTCTCATGCCCAGCGGTGCAGCAGCCTCCAACCCCCGCGATTTGGTAGCTGCTTGGCAGGAGGACCCGCAGTGCTCCACCCCTGAGGTTCCTCATGCTGAGACCACAGTAGGTCGCCTGGTTCAGTGTGAAGCGTTGTTGGGGATTCGCTCCAGGTGTAACCCAGTGGTTCACCCACAGCCATTCATCAGCATGTGTCACACTGCCCACAAGGCTTGCGATGCCGCCCAAGCTTACAGAACCATTTGCTCTCTGAGAGGAGTGGAAGAAGTTTTCCCTATGGCGTGCTAACAACACCTGTTGACATGTTAACATAGACATTAAATGTTGACGACTCATTCAGATTTTGGATATGGCTATAGATAACTGTATTTTTGTTACGTGAATGCATCATAAAATGTACAAAAAAAAATCCTCAATAAAATTGCAACCAACTACATTTCTGTTTTGTATTCCATTTGCATATCTGTTTCTATATCTTTTTAAGTATCTAATTCCGGAAACCATAACGAGAAAATATTGGATTACGATGTTGCAGAAATAAGAACATAGATTTATTTGCAGTTGGCAGTATGATATCAATTAGAATACAGAAGGCAACTGATTAAACAAAACAAGTATGACATAAAGATATAAACTATACAGTTCTTGTAAAATTTCCCCTTTGGCGTGCTAACAACACCTGTTG

>CL2883.Contig2_All

ACCTGCCACAAAGGCGAGAACGAGTAGTCGTGGTGAGCTCGTCCAGCCAACATGACGACCTCAACTCTCCTCTTCGTTCTCGCCTTTGTGGCAGGTTGTCTGGCAGCCCCCTGGGGAGCGGACGTGCCAAGATGCTCCACCGAATGCCCCGTCACCGGATCCCCCAAACTGGCCTACCAACCCGACAAGACCTACGCCTACCAATACTCCGGCAAGTCCAAGGTCCAGCTCAAGGGCGTGGACAACGGCGACTCGGAGACCGAGTGGACGGCACAAGTTGATCTCACCTGGATCAGCCCTTGCGACATGGCCATCTCCTTCAAGAATACCAAGGTGGATGGCACCCCCGGTCCCATCGTTGCCAGGACGCTGGAGAGACATCCACTGGTGGTGGCCGTCGTCGACGGAAGGGTGCAGCACGTGTGCGCTCACCCAGAGGACGAACCATGGGCCATCAACCTGAAAAAGGGCGTGGCTTCGGCTTTCCAGAACTCCATTCCTTCTCTGTCTGCTGTCAGCTCAGGCATCACAGTAACGGAGACTGATGTTGTGGGAAAATGCCCAACAAAGTATGAAATTGAGACCGAAGGAGAGAAAGTCATTGTCGTCAAGGAGAAGAACCACCGCCACTGTCAACAACGTTACCCAACACCCGCTCATATACCTGCACTATGGCTGAAGGCTCCCCTGCCAATCCAGGAATCCACGTCACAGTGCAAGCAGGAAATCGCCAATGGCATTTACACCGCCATCACGTGTCAGGACAAGAACATCGTTCGACCTGCCATTGGAATCTACAAGTACGTGGAGGCCAGTCAGGATTCAACACTTCGCTTCATCTCGGAGTCCTCCGACACTTCAGCTATCAGTGCCATCCCTTCAGGAGAAATGCAAGTTGAAAGCCTCCTGTACAACCACCAAACAATGAAGGACCCACAACTGGCACCTGAGCTGGATGAGCTCATGAAGGGGATCTGTGACAAGACCAAGGACACAGTTGAGGCTGAAGCTGCTGCTTTGGTTGCCAAGGCTCTCCATCTGTTACGTCGTGTTCCAGAGACAGTTGTGGTGGAGACTGCACAGAAAGTGAGACAAGGACATTACTGCAGTGACTCTGCCAGGCTGGAGAGTATCTTCTTGGACGCAGTTGCTTTCCTGCATGAGTCTGGTGCAGTACAGGTCATGGTCCAAGAAATCGAGAATGGACGAGCAACAGGGGGACGTCTCGCTCTGTACACGGCAGCGCTCTACCTCACCCCACGACCCAGCATTGAGGCAGTCAAGGCTCTCACGCCACTCTTTGAAAGCCCTCGCCCAGTGCCCTCGGTGTTGCTGGCAGCTGCTTCCATGATAAACCACTACTGCCTTCATACTCCAGCTTGCCACCAGAAAGCTCCAGTGGCGAGAATTGCAGAGATTCTGGCCACCAGAGTCCAGAGTCACTGCTCTCCTTCTGCTGGTGCTGAGGGCGAGGAAGTACCCCTTGCATTCTTCAAGGCAATAGGGAATATGGGTGTAGCTACACCTGCCGTGACAAGGGCAGCCGTCCAATGCATTGAAGAAGAAGGACTGGAAACCAGCATTCGGGTAGCTGCAGCACAAGCCTTCAGACAAGCCAATTGCTTCCGTCCAGCAGTTGAAAAGCTAGTAGACATTGCCGTCCGACCAGCCTTTGACACCGAAGTCCGCATCGCTTCCTATCTGGCAGCTGTCCGATGTGCTGAACAGGAACACCTGGAGAAAATTATTGAGAAGATTTCAAAGGAAGAGAATACTCAAGTGCGTGGATTTGTTTTGGGTCACCTGATCAACATCCAAGAGGGTAGCTGCCCCAACAAAGAAAACCTCAGGTACCTCCTTGCCAACGTTGTCATCCCTACCGACTTCGAGAAGGACTTCAGGAAATTCTCTCGACATATAGATATGGCTTACTATGCCCCTGCCTTTGGCATGGGTGCCGGCCTCGAGTCGAACATCATCTATGCTCCCGGATCTTTCATTCCTCGTGCTGTTAACCTGAACATGAGAGCAACTGTGGATGAGACGCCCATGGACATAGCAGAGATTGGTGCGCGCTTTGAAGGAGTCGACTCCATCATTGAAGAGCTCTTGGGCCCACAGGGATACCTACGCAAAGCAACATTTGGAAAGATTATGGAGGACATTACGGGTTTTGCAGGAGAGAAAGGCCTCAAGATCATGGAGCACATCAAGCACACAATGAGGACCAGGCGATCCATCGATGCTTCTGTCATCTCCGACTTCTTCGGCAAGCTGTATGGTGAGAGCAGTTCGCACACCCACGCCGATATATTCGCCCGGTTCATGGGACACGAGATTACTTTCGCAGATGTTGCCCAAAGCCTCAAGGGCGTCACAGCTGACACACTCATTGAGACCTTCTTCTCTTTCTTCGAGAATTCCTTGGAACATATGAAGGATCTTAACCTAAACACAGCAAGAACTGCTCAGCTTTCCATGGATTACTCACTACCCACCATTCAGGGCACACCACTCAAGCTGAACTTAGCTGCAACTGCTGTTGCTGGCCTCAAGATGGAGGGCAACGTCAACATTGGCCAGATCCTCTCTGACCTGGGCAATTCCCACACCGGCATCAAGGTGTTCCCAGGCCTTTCTGTACAAGCCACTGGTTTTGTTGGCTTTGAGTGCCGCTTTACCAAGGTGGGAATCGAGATGCAGAACACCATCTCTAGTGCCACTGGAGCCGCCATCAACATCAGAACAACTGAAAACAAGAAGATCGAGCTGGAATTGGAGATCCCTGACAAGATGGAACTCCTCAACATCAAGGCCGAGACTTACCTTGTCAAAGCTAGGGGAAAGAAGATGACTAAGATTTCTCCTTCCTCCATGAGAGATGTCAGGATTGAGCGCAAGTCCTGCATTGCTGCTTTGGAACCAGTATTTGGCCTCAAGGTGTGCTATGACATGAACTTCCCTGATGTGTTCCGTGCTAATGCCCTGCCACTTGGTGAACCAGCCATCGCCAAGCTGTACGTTGAGAAGGCAGATCCTTCCATGAGAGGTTACTTAGTGACTGCTGCCATCAAGAACAAGAGAGGTAACAAGCTCATTAAGATGAATGTAGAAGCAGCTGGTGCCTCAACACCAAGAAGAGCAGAAATGACCCTGTCCTACACCAAGGAAGAAGGAAGCCACATTGTTTCTGCCAAGCTTGATTCCTCCAGCATTGCTGCAGGAGTGTGGACTACTCTCACCAACGAGCAAGGACACAAGGCAGTAGAGACTTATGTCAACTTCAAATATGGTCAGACTGCTATTTCTCGAAGCATCAAGCTGGAAGCGATTGCAAGGGAAGGAAGTGTGGGAGAGGAATTCCAAGTGAACGTTTTCAGCAGCGGCACCAGGAGCTTCCCCTCTAAATCTCAAATTGTAGAGGCTAAATTCATCAAGAAAACTAGCGGACCCGTAGTTAATGTGGATGTGATTTGCAGGACCAGAAATGGTCTGGCTGAATACTTCAACCTCAATATGGAAGTGGGAGCTGACCTCATGGAGTTCTCCTCAGAGGACACATACAGGACCAGGTACATTCCCAAGTTCCGCATTCTTTTCCCTGTAACTTTGCGGAAGGTGGAAGTGCATGCCGAAACTGGAGCTTGGAGACTGGCATCATACATCCGCGAAGGCAGCCAGTCTGGACAAATCAGCGAGCACATTTCTGCACTCAGGCTAGCAAAGGGAAGCAAAGATATCATCTCTGTTGAAGCCACTCACACGATTGAGGGAGCTTTCCCGGAAAACATCATCATCAAAAATGAAGCAACAGTTGATATTGGCAGATCATCATACAAAGCCATCTATGATGTAATCTACCAGGCTGAAAAGGTCGGGGCTTCTATGGAAGTCGTCCGCACGGGAGACAATGAGAAAATTGTTAAACTGGACGCAATCTATCATCATTCAGGAGCAGAATACAGCACTAATTTCTTGCTGAAGGTTCCCACTTACATGAAACCTCTCAAGATTCAGGCCAAGATTGGAGAAGAAGCAGAGGGTCGCTACATGCTGGAAACAGCCATCAAATATGGTCAGCGTATGATACTTGAAGCTAATGGGCCAATCATGGCACGTCTTTCCTCCAAGATTGCAAAGATGCAGGCCAACATCAAACTCAGTGCATTGGCAAGTGAACCCTACATCATTGGTGCCAATGTTGTGTTTGGCAACAAGAAACAGGTGATAGCCATGGAAATCAAGGAGCGAGAAGAGCCACTCTTTGGTGTAGAATGGAAGATGGTTCAAGAGAGTTCTGAGAAGACCACTGTTGGCATAGCATTCGTTCTCCCTGCCCTTATTGAGAACAAGGTCGATGCCATTATTACTGAGGACCTTATCCATGTTAATTTTAACAACTTGGTTCTGCCTAATACTTCATCCCGCCGTCGGGTCAAGGGATTCACTGATGTCAACATTGCAGAGAAAAGGGCAAATGTGGAGTTTTCTTGGGATGCCGATAATGCTCCCGAAAAGAAGTTGGTGTTGGATGCAAGTCTGATCAGCAGTCCTGCCAACCCTGGACATGCTGAGATCCACGGGAATGTCGTCATTGCCGGAGAGCCTTACCACGCCAAATTGATTCTGACTGCCACAGATCTCATAGAGCACATGGAAGGGGAAAATGGATTCAAGTTGATCCTGACAACTCCTAGCCAGAAGACGGTTGTCGTGGGAGCCTCCTGTGATGTCCAGCTGGCAGGAGCCACCACTAAAGTCATTTCCACCGTTGAATACAAGAACGTGAGGGATAGGAAATACAAATATACAAGTGTGATTGCCTTGGAGAGGCTTGGTGGTCCACTTAATTATGCCGTAGAAGCCAAGGTAACTTACAAACAACCTGGAACAGCAGAAATAAAGGTAGAAACAACAGCTAAACATCATTGGACACCAGAAGAACATGTTGTAGCATTCAAGGTGGCTGCTGAAGCTCCAGTACTGAAGACGCCTGCCATGATTGCATTCTCCATTCACAATGCACCAAACGCTTTTGCTGGAGTCTGCAAGATCGAAAGAACTGCTCCTTTCACTGCCTTTGAATGGAATGTACAGGTTACTCCTGAAGGAGGAATTGAAGCTGTTGAAGCTGGTGTGGACATGAAAGCCATCATTGAAGTTCTGAAGATTGTTCGTGCCATTGCTACTCTGGAGGAAGAGAGTTATGAAACTTATGGCCCACACACAGCTCAGTACCAGTACCGCTTCACAAGGCCATCACCCACTTCTTACACCATGCAGATGAGGACTCCAACCCGCACCATGGAAGGAAGAGCTAAACTATCACCAAGGGAATCTGGAATCAAGTTCTACCCCAATAAGGGCAAAACTGAATCCAAATACGAAATTGGATACAAGGTCAACCACGAGGGAAGGTGGGGACAACGTGCGTCCAAGTTGGAAGTCAGAATGAACCATCCAGTGCTTCCTAAACCCATCATGGCCGCTGCTCAGTACACAGTAGCTGAAGGAACAATGAGGGGAACAATTGAACTGGACATTTTCCCAGAAGAAGCCGACAAAATTACTGGAACTGTGGAAACTCAGAGAATTTCAGAAAATGCTATCAGGGCAGAAGCCTTCTTGACTGGTAGGATGTTGAAAGTGAACCCTAAAGCTATCATCACTGCTGCCTATGCACCAGAAACAGTTGCTTTGGATGTAGTGTTCCACAAGACTCCGTCTGCAGCACCAATCTTCGCCATTGCTGCCAAGTATGACAAGACTGCAGCTCACAGTGCAGCTGCCACATTGACAGTAAAGATGGAAGAGCGACCTGTCTTTGAGATGAGTGCAGTGACCGAACCCGAGGAACCAGCCACCTGCAATGGCATCAGAATGAATGCTGTTGCTTATGCAGCAGCTTTTGGAAAGTACAACGTGTTCTCCAAGATGTGCAGGCCCGCCTTCATTGAGGTGACCGCAATGCGACCTGGTGGAGCAAAGGAGTACACTGCCAAGCTTGGCCTCCGATTCCCTGACGCTGCTGAAGCAGGCGTATATGTGGCGAGTGCTGGAGAGAGTCGCGGTGTTGCTGTTGCTGCTGTGAAGCTGGCTTCACCCAAAGTACTGAAAGTCGAGATGGCTTATGAGCCGCAAGAAGCAGAAGCAATAAGCATTGAAATGACTGAAGGATTTGAGAAGATTGCTGTATCATTCAAGTCTGTTGCAATGGAGTTCGTCCAATTCCTCAAGGAAGAGGCTGCTGCAAAGGGTGTTCAGTTCTCTTCATCTCAGTTAGTCAATTTAATGGGAGTTGCTAAGGAGGAAATTGTAGAGATCTATCGAGATATTCTCTCCGAGGCAAGAATTTTTCATACCGAAATCCTTGCTAATATTCTGGAAAGTCCTGTGGTATCCGTCATATCACGAGTCTACTTCGGTGTGCGGTCGGAAATTGTTCGCCTTCAACACCAGCTTTCCGTAACCCTCATCCAGGCGATAGAAGGAGGCCAGGAGGAATTAGCAATCGTTTATGAAATCGTAATGGAAGTTGTGATGACGGCAGCACGCATGGCAGAAACTGGAGAAGTCCCTGTGGCAGTGCTTGAGGCACTTGAGGAAATCAAAGCCAGCAAAGCTTTCAGGGTTGTGAAGAGAGAAGTGGATGTCATTTTGAGAGAATATCCGGAGGAGTATGAAGCTGTCAAGCACATCTTTGGCAACGTGGTGGCAATTCTCAAGCGAGATGTTGGCATTGTTCGTGAGTGGCTCATGGAGATTCCAGCTGTTCAGAGAGTCATCGACTACACCATGTATCACTTCCATTCGGAACGAGCATTTGCTGCAGAAGCAGAAAAGGTCGTTCGCCTCATTCTCGACGAACTTCTCTTCGTTTCAATGGAAAGCGAAGGCAACGGCGTTGCAGTCCGAATTCCCCTCCACCGACCCTTTTATTCACTAACGCAAGTGGCACAAGAAGCAGTGCCCAGCCCTGTCACAATGCTCGAGAACCTGATATTTGACTACCTTGAATACATTCCCATCCCTGTGAGCGACGCAATCTGGGCCTACTACAACTTCCTTCCACGCTACATCACGGACGCGCTGCCGCCCTACCCACGAACAGCCATGGTGGTTGGCGGCACTGAGATCCTCAGCTTCAGCGGCCTTGTTGTGCGAGCACCTCGCTCGCCCTGCAAGCTTCTCCTGGCTGCTCACGGCTCCCACCGCCTCATCATGTCCCACCCGCAAGCCTCAGCCCCGGCACAGCTTGAGCTCAAGACACCAGCAGCCACCGTGATCATCAAGCCTGACTTTGAAGTCCTGGTTAATGGCCAAGCCCTCGGGGGATCCCAGCAAACCATCGGAAACGTTAGGATTGTGAACACAGCCAAGCACATTGAGGTGGGATGTCCCCTGATGAGGGTGATCGTTGCCAAGGCAGGCGAGGCCGTAGCTGTTGAGGCTTCAGGCTGGATCTTTGGACGCGTAGCAGGGCTACTGGGCCCCAACACTGGAGAAATTGCCAATGACCGTCTCATGCCCAGCGGTGCAGCAGCCTCCAACCCCCGCGATTTGGTAGCTGCTTGGCAGGAGGACCCGCAGTGCTCCACCCCTGAGGTTCCTCATGCTGAGACCACAGTAGGTCGCCTGGTTCAGTGTGAAGCATTATTGGGGATTCGCTCAAGTTGTAACCCAGTGGTTCACCCACAGCCATTCATCAGCATGTGTCACACTGCCCACAAGGCTTGCGATGCCGCCCAAGCTTACAGAACCATTTGCTCTCTGAGAGGAGTGGAAGAAGTTTTCCCTATGGCGTGCTAACAACACCTGTTGACATGTTAACATAGACCTAGAATGTTGACGACTCATTCAGATTTTGGATATGGCTATAGATAACTGTATTTTTGTTACGTGAATGCATCATAAAAAAAAAAAAAAAAAAAATCCAAGTACTATTATTTACGAGAAGACAGAATATCTTGGAAAGCCTCGCAAGTCCATTAATATCTCTCAGAAACCGTATGAATCCAGGAAGAAAAAACTCAAATTCTGCATTATGCAATTTTGATCTTTTTGGATGATAAATTCTTCACATCAAAAAAACTGTCTTGGATCTCATATGTCATACTGGCGGCAGTGAGATCGTCACATCAGTGAAGGGAGTATTCATTCCGTAATGATTCATTTCCAAAGAATGATAAGTCATTTGTAATGTATGATTTAAAACAAGATGTTAGAATAGTTAATTATGCTAAATTCACATTATATTAGGCTGCATCATGTTTTATATATAAATTAATGCATCGTATTCATCTTCATTAAAACATACTCATTTGAGTACAGTTTATATAGACCTCAACTGCCATTGTCATTGTCATCATCATTGCTATTCTCGGTTACTTTGTATTAATTAGCTTAATAAAGTATGGATGATTTTTTCTTCCGTTCAACATTTACTTTTATTCATCACTTTAAGTAATGAATGGAAGCCTATTTATATTTGTATATATTATAGAGATTAAATATTTTTAATAAAAGTGTACCTCACTTATCTCTCTCTCCTTTGAATTAGATTCATCATACCCTCTGAAACGTCCCCATGACTTCACCAGAAAAGTAACTTTTTTATGAGAATCTTGAATGTTACAATTACTGTCCTTTTCCTCTGATGATTTTTGGTCGTTCTGTTTTGGTGTTGGCTTCGTTCACCCTTTCAGTATTTAATTATTTATTTTTTCTTGCATTTTCATTTATCAATTTTTTCATTATTTCCTTTTATGTGTTGTTTTTTTCCCTTTCAATAAAAACATCTCCAAAAGTGTGGATATGAAGGTATATTACTGATAATTTACAAGTGCATTTGTG

>CL2833.Contig3

CCAAGATGCTCCACCGAATGCCCCGTCACCGGATCCCCCAAACTGGCCTACCAACCCGACAAGACCTACGCCTACCAATACTCCGGCAAGTCCAAGGTCCAGCTCAAGGGCGTGGACAACGGCGACTCGGAGACCGAGTGGACGGCACAAGTTGATCTCACCTGGATCAGCCCTTGCGACATGGCCATCTCCTTCAAGAATACCAAGGTGGATGGCACCCCCGGTCCCATCGTTGCCAGGACGCTGGAGAGACATCCACTGGTGGTGGCCGTCGTCGACGGAAGGGTGCAGCACGTGTGCGCTCACCCAGAGGACGAACCATGGGCCATCAACCTGAAAAAGGGCGTGGCTTCGGCTTTCCAGAACTCCATTCCTTCTCTGTCTGCTGTCAGCTCAGGCATCACAGTAACGGAGACTGATGTTGTGGGAAAATGCCCAACAAAGTATGAAATTGAGACCGAAGGAGAGAAAGTCATTGTCGTCAAGGAGAAGAACCACCGCCACTGTCAACAACGTTACCCAACACCCGCTCATATACCTGCACTATGGCTGAAGGCTCCCCTGCCAATCCAGGAATCCACGTCACAGTGCAAGCAGGAAATCGCCAATGGCATTTACACCGCCATCACGTGTCAGGACAAGAACATCGTTCGACCTGCCATTGGAATCTACAAGTACGTGGAGGCCAGTCAGGATTCAACACTTCGCTTCATCTCGGAGTCCTCCGACACTTCAGCTATCAGTGCCATCCCTTCAGGAGAAATGCAAGTTGAAAGCCTCCTGTACAACCACCAAACAATGAAGGACCCACAACTGGCACCTGAGCTGGATGAGCTCATGAAGGGGATCTGTGACAAGACCAAGGACACAGTTGAGGCTGAAGCTGCTGCTTTGGTTGCCAAGGCTCTCCATCTGTTACGTCGTGTTCCAGAGACAGTTGTGGTGGAGACTGCACAGAAAGTGAGACAAGGACATTACTGCAGTGACTCTGCCAGGCTGGAGAGTATCTTCTTGGACGCAGTTGCTTTCCTGCATGAGTCTGGTGCAGTAAAGGTCATGGTCCAAGAAATCGAGAATGGACGAGCAACAGGGGGACGTCTCGCTCTGTACACGGCAGCGCTCTACCTCATCCCACGACCCAGCATTGAGGCAGTCAAGGCTCTCACGCCACTCTTTGAAAGCCCTCGCCCAGTGCCCTCGGTGCTGCTGGCAGCTGCTTCCATGATAAACCACTACTGCCTTCATACTCCAGCTTGCCACCAGAAAGCTCCAGTGGCGAGAATTGCAGAGATTCTGGCCACCAGAGTCCAGAGTCACTGCTCTCCTTCTGCTGGTGCTGAGGGCGAGGAAGTACCCCTTGCATTCTTCAAGGCAATAGGGAATATGGGTGTAGCTACACCTGCCGTGACAAGGGCAGCCGTCCAATGCATTGAAGAAGAAGGACTGGAAACCAGCATTCGGGTAGCTGCAGCACAAGCCTTCAGACAAGCCAATTGCTTCCGTCCAGCAGTTGAAAAGCTAGTAGACATTGCCGTCCGACCAGCCTTTGACACCGAAGTCCGCATCGCTTCCTATCTGGCAGCTGTCCGATGTGCTGAACAGGAACACCTGGAGAAAATTATTGAGAAGATTTCAAAGGAAGAGAATACCCAAGTGCGTGGATTTGTTTTGGGTCACCTGATCAACATCCAAGAGGGTAGCTGCCCCAACAAAGAAAACCTCAGGTACCTCCTTGCCAACGTTGTCATCCCTACCGACTTCGAGAAGGACTTCAGGAAATTCTCTCGACATATAGATATGGCTTACTATGCCCCTGCCTTTGGCATGGGTGCCGGCCTCGAGTCGAACATCATCTATGCTCCCGGATCTTTCATTCCTCGTGCTGTTAACCTGAACATGAGAGCAACTGTGGATGAGACGCCCATGGACATAGCAGAGATTGGTGCGCGCTTTGAAGGAGTAGATTCCATCATTGAAGAGCTCTTGGGCCCACAGGGATACCTACGCAAAGCAACATTTGGAAAGATTATGGAGGACATTACGGGTTTTGCAGGAGAGAAAGGCCTCAAGATCATGGAGCACTTCAAGCACACAATGAGGACCAGGCGATCCATCGATGCTTCTGTCATCTCCGACTTCTTCGGCAAGCTGTATGGTGAGAGCAGTTCGCACACCCACGCCGATATATTCGCCCGGTTCATGGGGCACGAGATTACTTTCGCAGATGTTGCCCAAAGCCTCAAGGGTGTCACAGCTGACACACTCATTGAGACTTTCTTCTCTTTCTTCGAGAATTCCTTGGAACATATGAAGGATCTTAACCTGAACACAGCAAGAACTGCTCAGCTTTCCATGGATTACTCACTGCCCACCATTCAGGGCACACCACTCAAGCTGAACTTAGCTGCAACTGCTGTTGCTGGCCTCAAGATGGAGGGCAACGTCAACATTGGCCAGATCCTCTCTGACCTGGGCAATTCCCACACCGGCATCAAGGTGTTCCCAGGCCTTTCTGTACAAGCCACTGGTTTTGTTGGCTTTGAGTGCCGCTTTACCAAGGTGGGAATCGAGATGCAGAACACCATCTCTAGTGCCACTGGAGCCGCCATCAACATCAGAACAACTGAAAACAAGAAGATCGAGCTGGAATTGGAGATCCCTGACAAGATGGAACTCCTCAACATCAAGGCCGAGACTTACCTTGTCAAAGCTAGGGGAAAGAAGATGACTAAGATTTCTCCTTCCTCCATGAGAGATGTCAGGATTGAGCGCAGGTCCTGCATTGCTGCTTTGGAACCAGTATTTGGCCTCAAGGTGTGCTATGACATGAACTTCCCTGATGTGTTCCGTGCTAATGCCCTGCCACTTGGTGAACCAGCCATCGCCAAGCTGTACGTTGAGAAGGCAGATCCTTCCATGAGAGGTTACTTAGTGACTGCTGCCATCAAGAACAAGAGAGGTAACAAGCTCATTAAGATGAATGTAGAAGCAGCTGGTGCCTCAACACCAAGAAGAGCAGAAATGACCCTGTCCTACACCAAGGAAGAAGGAAGCCACATTGTTTCTGCCAAGCTTGATTCCTCCAGCATTGCTGCAGGAGTGTGGACTACTCTCACCAACGAGCAAGGACACAAGGCAGTAGAGACTTATGTCAACTTCAAATATGGTCAGACTGCTATTTCTCGAGGCATCAAGCTGGAAGCGATTGCAAGGGAAGGAAGCGTGGGAGAGGAATTCCAAGTGAACGTTTTCAGCAGCGGCACCAGGAGCTTCCCCTCTAAATCTCAAATTGTAGAGGCTAAATTCATCAAGAAAACTAGCGGACCCGTAGTTAATGTGGATGTGATTTGCAGGACCAGAAATGGTCTGGCTGAATACTTCAACCTCAATATGGAAGTGGGAGCTGACCTCATGGAGTTCTCCTCAGAGGACACATACAGGACCAGGTACATTCCCAAGTTCCGCATTCTTTTCCCTGTAACTTTGCGGAAGGTGGAAGTGCATGCCGAAACTGGAGCTTGGAGACTGGCATCATACATCCGCGAAGGCAGCCAGTCTGGACAAATCAGCGAGCACATTTCTGCACTCAGGCTAGCAAAGGGAAGCAAAGATATCATCTCTGTTGAAGCCACTCACACGATTGAGGGAGCTTTCCCGGAAAACATCATCATCAAAAATGAAGCAACAGTTGATATTGGCAGATCATCATACAAAGCCATCTATGATGTAATCTACCAGGCTGAAAAGGTCGGGGCTTCTATGGAAGTCGTCCGCACGGGAGACAATGAGAAAATTGTTAAACTGGACGCAATCTATCATCATTCAGGAGCAGAATACAGCACTAATTTCTTGCTGAAGGTTCCCACTTACATGAAACCTCTCAAGATTCAGGCCAAGATTGGAGAAGAAGCAGAGGGTCGCTACATGCTGGAAACAGCCATCAAATATGGTCAGCGTATGATACTTGAAGCTAATGGGCCAATCATGGCACGTCTTTCCTCCAAGATTGCAAAGATGCAGGCCAACATCAAACTCAGTGCATTGGCAAGTGAACCCTACATCATTGGTGCCAATGTTGTGTTTGGCAACAAGAAACAGGTGATAGCCATGGAAATCAAGGAGCGAGAAGAGCCACTCTTTGGTGTAGAATGGAAGATGGTTCAAGAGAGTTCTGAGAAGACCACTGTTGGCATAGCATTCGTTCTCCCTGCCCTTATTGAGAACAAGGTCGATGCCATTATTACTGAGGAACTTATCCATGTTAATTTTAACAACTTGGTTCTGCCTAATACTTCATCCCGCCGTCGGGTCAAGGGATTCACTGATGTCAACATTGCAGAGAAAAGGGCAAATGTGGAATTTTCTTGGGATGCCGATAATGCTCCCGAAAAGAAGTTGGTGTTGGATGCAAGTCTGATCAGCAGTCCTGCCAACCCTGGACATGCTGAGATCCACGGGAATGTCGTCATTGCCGGAGAGCCTTACCACGCCAAATTGATTCTGACTGCCACAGATCTCATAGAGCACATGGAAGGGGAAAATGGATTCAAGTTGATCCTGACAACTCCTAGCCAGAAGACGGTTGTCGTGGGAGCCTCCTGTGATGTCCAGCTGGCAGGAACCACCACTAAAGTCCTTTCCACCGTTGAATACAAGAACGTGAGGGATAGGAAATACAAATATACAAGTGTGATTGCCTTGGAGAGGCTTGGTGGTCCACTTAATTATGCCGTAGAAGCCAAGGTAACTTACAAACAACCTGGAACAGCAGAAATAAAGGTAGAAACAACAGCAAAACATCATTGGACACCAGAAGAACATGTTGTAGCATTCAAGGTGGCTGCTGAAGCTCCAGTACTGAAGACGCCTGCCATGATTGCATTCTCCATTCACAATGCACCAAACGCTTTTGTTGGAGTCTGCAAGATCGAAAGAACTGCTCCTTTCACTGCCTTTGAATGGAATGTACAGGTTACTCCTGAAGGAGGAATTGAAGCTGTTGAAGCTGGTGTGGACATGAAAGCCATCATTGAAGTTCTGAAGATTGTTCGTGCCATTGCTACTCTGGAGGAAGAGAGTTATGAAACTTATGGCCCACACACAGCTCAGTACCAGTACCGCTTCACAAGGCCATCACCCACTTCTTACACCATGCAGATGAGGACTCCAACCCGCACCATGGAAGGAAGAGCTAAACTATCACCAAGGGAATCTGGAATCAAGTTCTACCCCAATAAGGGCAAAACTGAATCCAAATACGAAATTGGATACAAGGTCAACCACGAGGGAAGGTGGGGACAACGTGCGTCCAAGTTGGAAGTCAGAATGAACCATCCAGTGCTTCCTAAACCCATCATGGCCGCTGCTCAGTACACAGTAGCTGAAGGAACAATGAGGGGAACAATTGAACTGGACATTTTCCCAGAAGAAGCCGACAAAATTACTGGAACTGTGGAAACTCAGAGAATTTCAGAAAATGCTATCAGGGCAGAAGCCTTCTTGACTGGTAGGATGTTGAAAGTGAACCCTAAAGCTATCATCACTGCTGCCTATGCACCAGAAACAGTTGCTTTGGATGTAGTGTTCCACAAGACTCCATCTGCAGCACCAATCTTCGCCATTGCTGCCAAGTATGACAAGACTGCAGCTCACAGTGCAGCTGCCACATTGACAGTAAAGATGGAAGAGCGACCTGTCTTTGAGATGAGTGCAGTGACCGAACCCGAGGAACCAGCCACCTGCAATGGCATCAGAATGAATGCTGTTGCTTATGCAGCAGCTTTTGGAAAGTACAACGTGTTCTCCAAGATGTGCAGGCCCGCCTTCATTGAGGTGACCGCAATGCGACCTGGTGGAGCAAAGGAGTACACTGCCAAGCTTGGCCTCCGATACCCTGACGCTGCTGAAGCAGGCGTATATGTGGCGAGTGGCAGAGCTGGAGAGAGTCGCGGTGTTGCTGTTGCTGCTGTGAAGCTGGCTTCACCCACAATGCTACAGTTCGAGGTGGCTCATGAACCAGAAGAAGCACACATTGTAATGAGTGAAGTGACAAGTACCCTCAGAAAAGTCGCCATGTCTCTCGAAACAGTTGCAATGGAGGCCGTCCAGTTCCTCAAGGAAGAAGCTGCTGCAAAGGGTGTCGAGTTCCCGTCATCTCACTTTGTCAGTCTAGTGGATGAGGCGAATGAGGAAATCAAAGCCATTTACCGAGATATCGTCTCAGAGATGAGAATCCTTGACACTGAGTTGATTGCTGATATCTTGGAAAGCCCTACGGTGTCCTTCGTGTCGCGTGTCTACCTTGGAGTATGGTCACAGATTGCTCGCCTTCAACATCACTTTTCAACCAGGGCGGTTGAAATGATCCAGCAATGGCAGGAACAACTAACAGACGTTTCTGAAATCTTTATCGAAGCTGTTATGGAGATAGTGCAACTCTTGGAAGCCGGAGAAGTACCTGAAACAGTTCGTGTAATTCTGGAAAAAATTGAGAACACTGAGGTGTTCAGGATTGTAAAGAGAGAAGTGAACGCAGTGTTGGCAGAGTATCCTGAGGAGTATGAGGCCGTCAAGCACATCCTCACCAGGGTGACGGCCACTCTCAAGCACGATGCTGATATTGTGTACAAGAGGATCATGGAGACACCAGCTGTTCAAAGGATCTTTGCCTATGTTATGCAGTACATCAACTCGGAGCGCGTGTTTGCTGAGGAAGCAGGAAGTGTTGCCAGCCTCATTCTCAAAGAATTTCTTTTCGTTTCAATTGAAAGCGAAGGCAACGGCATTGCAGTCCGAATTCCCCTCCACCGACCCTTGTATTCACTGACGCAAGTGGCACAAGAAGCAGTGCCCAACCCTGTCACAATGCTCGAGAACCTGATATTTGCCTACCTTGAATACATTCCCATCCCTGTGAGCGACGCAATCTGGGCCTACTACAACTTCCTTCCACGCTACATCACGGACGCGCTGCCGCCCTACCCACGAACAGCCATGGTGGTTGGCGGCACTGAGATCCTCAGCTTCAGCGGCCTTGTTGTGCGAGCACCTCGCTCGCCCTGCAAGCTTCTCCTGGCTGCTCACGGCTCCCACCGCCTCATCATGTCCCACCCGCAAGCCTCAGCCCCGGCACAGCTTGAGCTCAAGACACCAGCAGCCACCGTGATCATCAAGCCTGACTTTGAAGTCCTGGTTAATGGCCAAGCCCTCGGGGGATCCCAGCAAACCATCGGAAACGTTAGGATTGTGAACACAGCCAAGCACATTGAGGTGGGATGTCCCCTGATGAGGGTGATCGTTGCCAAGGCAGGCGAGGCCGTAGCTGTTGAGGCTTCAGGCTGGATCTTTGGACGCGTAGCAGGGCTACTGGGCCCCAACACTGGAGAAATTGCCAATGACCGTCTCATGCCCAGCGGTGCAGCAGCCTCCAACCCCCGCGATTTGGTAGCTGCTTGGCAGGAGGAGCCGCAGTGCTCCACCCCTGAGGTTCCTCATGCTGAGACCACAGTAGGTCGCCTGGTTCAGTGTGAAGCGTTGTTGGGGATTCGCTCCAGGTGTAACCCAGTGGTTCACCCACAGCCATTCATCAGCATGTGTCACACTGCCCACAAGGCTTGCGATGCCGCCCATGCTTACAGAACCATTTGCTCTCTGAGAGGAGTGGAAGAAGTTTTCCCTATGGCGTGCTAACAACACCTGTTGACATGTTAACATAGACATTAAATGTTGACGACTCATTCAGATTTTGGATATGGCTATAGGTATTAACTGTATTTTTGTTACGTAAATGCATCATAAAATGTACAAAAAAAAAAATCCTCAATAAAATTGCAACCAAC

>CL2883.Contig4_All

CCGGGGAGTAGTCTTGGTGATCGCTCAGAACCACCATGACGACCTCACAGCTCTTCTTGGTTCTCGCCCTCGTGGCAGGCAGCCTGGCAGCCCCCTGGGGAGCGGACGTGCCAAGATGCTCCACCGAATGCCCCGTCACCGGATCCCCCAAACTGGCCTACCAACCCGACAAGACCTACGCCTACCAATACTCCGGCAAGTCCAAGGTCCAGCTCAAGGGCGTGGACAACGGCGACTCGGAGACCGAGTGGACGGCACAAGTTGATCTCACCTGGATCAGCCCTTGCGACATGGCCATCTCCTTCAAGAATACCAAGGTGGATGGCACCCCCGGTCCCATCGTTGCCAGGACGCTGGAGAGACATCCACTGGTGGTGGCCGTCGTCGACGGAAGGGTGCAGCACGTGTGCGCTCACCCAGAGGACGAACCATGGGCCATCAACCTGAAAAAGGGCGTGGCTTCGGCTTTCCAGAACTCCATTCCTTCTCTGTCTGCTGTCAGCTCAGGCATCACAGTAACGGAGACTGATGTTGTGGGAAAATGCCCAACAAAGTATGAAATTGAGACCGAAGGAGAGAAAGTCATTGTCGTCAAGGAGAAGAACCACCGCCACTGTCAACAACGTTACCCAACACCCGCTCATATACCTGCACTATGGCTGAAGGCTCCCCTGCCAATCCAGGAATCCACGTCACAGTGCAAGCAGGAAATCGCCAATGGCATTTACACCGCCATCACGTGTCAGGACAAGAACATCGTTCGACCTGCCATTGGAATCTACAAGTACGTGGAGGCCAGTCAGGATTCAACACTTCGCTTCATCTCGGAGTCCTCCGACACTTCAGCTATCAGTGCCATCCCTTCAGGAGAAATGCAAGTTGAAAGCCTCCTGTACAACCACCAAACAATGAAGGACCCACAACTGGCACCTGAGCTGGATGAGCTCATGAAGGGGATCTGTGACAAGACCAAGGACACAGTTGAGGCTGAAGCTGCTGCTTTGGTTGCCAAGGCTCTCCATCTGTTACGTCGTGTTCCAGAGACAGTTGTGGTGGAGACTGCACAGAAAGTGAGACAAGGACATTACTGCAGTGACTCTGCCAGGCTGGAGAGTATCTTCTTGGACGCAGTTGCTTTCCTGCATGAGTCTGGTGCAGTAAAGGTCATGGTCCAAGAAATCGAGAATGGACGAGCAACAGGGGGACGTCTCGCTCTGTACACGGCAGCGCTCTACCTCATCCCACGACCCAGCATTGAGGCAGTCAAGGCTCTCACGCCACTCTTTGAAAGCCCTCGCCCAGTGCCCTCGGTGCTGCTGGCAGCTGCTTCCATGATAAACCACTACTGCCTTCATACTCCAGCTTGCCACCAGAAAGCTCCAGTGGCGAGAATTGCAGAGATTCTGGCCACCAGAGTCCAGAGTCACTGCTCTCCTTCTGCTGGTGCTGAGGGCGAGGAAGTACCCCTTGCATTCTTCAAGGCAATAGGGAATATGGGTGTAGCTACACCTGCCGTGACAAGGGCAGCCGTCCAATGCATTGAAGAAGAAGGACTGGAAACCAGCATTCGGGTAGCTGCAGCACAAGCCTTCAGACAAGCCAATTGCTTCCGTCCAGCAGTTGAAAAGCTAGTAGACATTGCCGTCCGACCAGCCTTTGACACCGAAGTCCGCATCGCTTCCTATCTGGCAGCTGTCCGATGTGCTGAACAGGAACACCTGGAGAAAATTATTGAGAAGATTTCAAAGGAAGAGAATACCCAAGTGCGTGGATTTGTTTTGGGTCACCTGATCAACATCCAAGAGGGTAGCTGCCCCAACAAAGAAAACCTCAGGTACCTCCTTGCCAACGTTGTCATCCCTACCGACTTCGAGAAGGACTTCAGGAAATTCTCTCGACATATAGATATGGCTTACTATGCCCCTGCCTTTGGCATGGGTGCCGGCCTCGAGTCGAACATCATCTATGCTCCCGGATCTTTCATTCCTCGTGCTGTTAACCTGAACATGAGAGCAACTGTGGATGAGACGCCCATGGACATAGCAGAGATTGGTGCGCGCTTTGAAGGAGTCGACTCCATCATTGAAGAGCTCTTGGGCCCACAGGGATACCTACGCAAAGCAACATTTGGAAAGATTATGGAGGACATTACGGGTTTTGCAGGAGAGAAAGGCCTCAAGATCATGGAGCACTTCAAGCACACAATGAGGACCAGGCGATCCATCGATGCTTCTGTCATCTCCGACTTCTTCGGCAAGCTGTATGGTGAGAGCAGTTCGCACACCCACGCCGATATATTCGCCCGGTTCATGGGGCACGAGATTACTTTCGCAGATGTTGCCCAAAGCCTCAAGGGTGTCACAGCTGACACACTCATTGAGACTTTCTTCTCTTTCTTCGAGAATTCCTTGGAACATATGAAGGATCTTAACCTGAACACAGCAAGAACTGCTCAGCTTTCCATGGATTACTCACTGCCCACCATTCAGGGCACACCACTCAAGCTGAACTTAGCTGCAACTGCTGTTGCTGGCCTCAAGATGGAGGGCAACGTCAACATTGGCCAGATCCTCTCTGACCTGGGCAATTCCCACACCGGCATCAAGGTGTTCCCAGGCCTTTCTGTACAAGCCACTGGTTTTGTTGGCTTTGAGTGCCGCTTTACCAAGGTGGGAATCGAGATGCAGAACACCATCTCTAGTGCCACTGGAGCCGCCATCAACATCAGAACAACTGAAAACAAGAAGATCGAGCTGGAATTGGAGATCCCTGACAAGATGGAACTCCTCAACATCAAGGCCGAGACTTACCTTGTCAAAGCTAGGGGAAAGAAGATGACTAAGATTTCTCCTTCCTCCATGAGAGATGTCAGGATTGAGCGCAGGTCCTGCATTGCTGCTTTGGAACCAGTATTTGGCCTCAAGGTGTGCTATGACATGAACTTCCCTGATGTGTTCCGTGCTAATGCCCTGCCACTTGGTGAACCAGCCATCGCCAAGCTGTACGTTGAGAAGGCAGATCCTTCCATGAGAGGTTACTTAGTGACTGCTGCCATCAAGAACAAGAGAGGTAACAAGCTCATTAAGATGAATGTAGAAGCAGCTGGTGCCTCAACACCAAGAAGAGCAGAAATGACCCTGTCCTACACCAAGGAAGAAGGAAGCCACATTGTTTCTGCCAAGCTTGATTCCTCCAGCATTGCTGCAGGAGTGTGGACTACTCTCACCAACGAGCAAGGACACAAGGCAGTAGAGACTTATGTCAACTTCAAATATGGTCAGACTGCTATTTCTCGAGGCATCAAGCTGGAAGCGATTGCAAGGGAAGGAAGCGTGGGAGAGGAATTCCAAGTGAACGTTTTCAGCAGCGGCACCAGGAGCTTCCCCCTCGACTCTCACATTGTGGAGGCTAAATTCATCAAGAAAACTAGTGGACCTGAATTCAATGTGGATGTGATCTGCATGACCAAAAATGCTTTAGCTGATTATTTCGACTTAAACATTGAAGTTGGAGCTGATTTCATGAGATTTTCTCCTAAAGCTCTGTATTCAACAAGATACATTCCCAAGACCCGCATTTTCTTACCTGTAAACCTGCGAAAGCTAGAAATCAATGCTGCCACTGCAGCCTGGAAAGTGACGTCGTACATTCGTGAAGGAAGTCAATCTGGCGAAAGCCGTGAGTTCAGTTCTGCTTTCAAGCTTGCCAAGGGAAGGACGGATGTCATCTTTGTACAGGCTACTCATACGATTGAAGGCAGATTCCCACAAAACGTCATCATCAAAAATGTAGCAACAGCCAAAGTTGGCAGATCATCATACAGAGCAATGTATGATGTCTTCTATCACCCTGAAAAAGTGGGAGCTTCTATTGAGGTTTTGCAGGCAGCAGGTAATGAGAAGGTTGCCCAGATAGAAGCAATTTACGAAATTTCCGGAGAGAAGCACTGCGCTAAATTCTTGGCGGCCATTCCTGGCTACATTCAACCAGTTAAAGTTGAAGCCGAGATTGAACAAGAAGCAGAAGGTCGCTACGCACTGGAGGCCGCCATCAAATATGGACCACGTACAGTACTTGGAGTGAGTGGACCAGTCCTAGCTCGTTTCACCTCCAAAGCCAACAAGCTGCAAGCCAACATCAAGCTCAGGGCAATGGCAAGTGAGCCCTACATCATTGGTGCCAATGTTGTGTTTGGCAACAAGAAACAGATGATCGCCATGGAAATCAAGGAGCGATCAGAACCTCTCATTGGTCTTGAATGGAGAATGGTCCGAGAAAGTTCCGAGAAGACCACTATTGGTGTAGTGTTTGTCCTCCCTGCCCTTATTGAGCAGAAAGTCGATGCTGAAATTACTGATGAACTTGTCCATGTTAGTTTCAACAACCTGGTTCTACCCAAGACTTCATCCCGCCGTCGAGTCAAGGGATTCGCTGATGTCAACATTGCAGAGAAAAGGGCAAATGTGGAATTTTCTTGGGATGCCGATAATGCTCCCGAAAAGAAGTTGGTGTTGGATGCAAGTCTGATCAGCAGTCCTGCCAACCCTGGACATGCTGAGATCCACGGGAATGTCGTCATTGCCGGAGAGCCTTACCACGCCAAACTGGTTCTGACTGCCACAAATCTCGTAGAGCACATGGAAGGGGAAAATGGATTCAAGTTGATCCTGACAACTCCTAGCCAGAAGACGGTTGTCGTGGGAGCCTCCTGTGATGTCCAGCTGGCAGGAACCACCACTAAAGTCCTTTCCACCGTTGAATACAAGAACGTGAGGGATAGGAAATACAAATATACAAGTGTGATTGCCTTGGAGAGGCTTGGTGGTCCACTTAATTATGCCGTAGAAGCCAAGGTAACTTACAAACAACCTGGAACAGCAGAAATAAAGGTAGAAACAACAGCAAAACATCATTGGACACCAGAAGAACATGTTGTAGCATTCAAGGTGGCTGCTGAAGCTCCAGTACTGAAGACGCCTGCCATGATTGCATTCTCCATTCACAATGCACCAAACGCTTTTGTTGGAGTCTGCAAGATCGAAAGAACTGCTCCTTTCACTGCCTTTGAATGGAATGTGCAGGTTACTCCTGAAGGAGGAATTGAAGCTGTTGAAGCTGGTGTGGACATGAAAGCCATCATTGAAGTTCTGAAGATTGTTCGTGCCATTGCTACTCTGGAGGAAGAGAGTTATGAAACTTATGGCCCACACACAGCTCAGTACCAGTACCGCTTCACAAGGCCATCACCCACTTCTTACACCATGCAGATGAGGACTCCAACCCGCACCATGGAAGGAAGAGCTAAACTATCACCAAGGGAATCTGGAATCAAGTTCTACCCCAATAAGGGCAAAACTGAATCCAAATACGAAATTGGATACAAGGTCAACCACGAGGGAAGGTGGGCACAACGTGCGTCCAAGTTGGAAGTCAGAATGAACCATCCAGTGCTTCCTAAACCCATCATGGCCGCTGCTCAGTACACAGTAGCTGAAGGAACAATGAGGGGAACAATTGAACTGGACATTTTCCCAGAAGAAGCCGACAAAATTACTGGAACTGTGGAAACTCAGAGAATTTCAGAAAATGCTATCAGGGCAGAAGCCTTCTTGACTGGTAGGATGTTGAAAGTGAACCCTAAAGCTATCATCACTGCTGCCTATGCACCAGAAACAGTTGCTTTGGATGTAGTGTTCCACAAGACTCCGTCTGCAGCACCAATCTTCGCCATTGCTGCCAAGTATGACAAGACTGCAGCTCACAGTGCAGCTGCCACATTGACAGTAAAGATGGAAGAGCGACCTGTCTTTGAGATGAGTGCAGTGACCGAACCCGAGGAACCAGCCACCTGCAATGGCATCAGAATGAATGCTGTTGCTTATGCAGCAGCTTTTGGAAAGTACAACGTGTTCTCCAAGATGTGCAGGCCCGCCTTCATTGAGGTGACCGCAATGCGACCTGGTGGAGCAAAGGAGTACACTGCCAAGCTTGGCCTCCGATACCCTGACGCTGCTGAAGCAGGCGTATATGTGGCGAGTGCTGGAGAGAGTCGCGGTGTTGCTGTTGCTGCTGTGAAGCTGGCTTCACCCACAATGCTACAGTTCGAGGTGGCTCATGAACCAGAAGAAGCACACATTGTAATGAGTGAAGTGACAAGTACCCTCAGAAAAGTCGCCATGTCTCTCGAAACAGTTGCAATGGAGGCCGTCCAGTTCCTCAAGGAAGAAGCTGCTGCAAAGGGTGTCGAGTTCCCGTCATCTCACTTTGTCAGTCTAGTGGATGAGGCGAATGAGGAAATCAAAGCCATTTACCGAGATATCGTCTCAGAGATGAGAATCCTTGACACTGAGTTGATTGCTGATATCTTGGAAAGCCCTACGGTGTCCTTCGTGTCGCGTGTCTACCTTGGAGTATGGTCACAGATTGCTCGCCTTCAACATCACTTTTCAACCAGGGCGGTTGAAATGATCCAGCAATGGCAGGAACAACTAACAGACGTTTCTGAAATCTTTATCGAAGCTGTTATGGAGATAGTGCAACTCTTGGAAGCCGGAGAAGTACCTGAAACAGTTCGTGTAATTCTGGAAAAAATTGAGAACACTGAGGTGTTCAGGATTGTAAAGAGAGAAGTGAACGCAGTGTTGGCAGAGTATCCTGAGGAGTATGAGGCCGTCAAGCACATCCTCACCAGGGTGACGGCCACTCTCAAGCACGATGCTGATATTGTGTACAAGAGGATCATGGAGACACCAGCTGTTCAAAGGATCTTTGCCTATGTTATGCAGTACATCAACTCGGAGCGCGTGTTTGCTGAGGAAGCAGGAAGTGTTGCCAGCCTCATTCTCAAAGAATTTCTTTTCGTTTCAATTGAAAGCGAAGGCAACGGCATTGCAGTCCGAATTCCCCTCCACCGACCCTTGTATTCACTGACGCAAGTGGCACAAGAAGCAGTGCCCAACCCTGTCACAATGCTCGAGAACCTGATATTTGCCTACCTTGAATACATTCCCATCCCTGTGAGCGACGCAATCTGGGCCTACTACAACTTCCTTCCACGCTACATCACGGACGCGCTGCCGCCCTACCCACGAACAGCCATGGTGGTTGGCGGCACTGAGATCCTCAGCTTCAGCGGCCTTGTTGTGCGAGCACCTCGCTCGCCCTGCAAGCTTCTCCTGGCTGCTCACGGCTCCCACCGCCTCATCATGTCCCACCCGCAAGCCTCAGCCCCGGCACAGCTTGAGCTCAAGACACCAGCAGCCACCGTGATCATCAAGCCTGACTTTGAAGTCCTGGTTAATGGCCAAGCCCTCGGGGGATCCCAGCAAACCATCGGAAACGTTAGGATTGTGAACACAGCCAAGCACATTGAGGTGGGATGTCCCCTGATGAGGGTGATCGTTGCCAAGGCAGGCGAGGCCGTAGCTGTTGAGGCTTCAGGCTGGATCTTTGGACGCGTAGCAGGGCTACTGGGCCCCAACACTGGAGAAATTGCCAATGACCGTCTCATGCCCAGCGGTGCAGCAGCCTCCAACCCCCGCGATTTGGTAGCTGCTTGGCAGGAGGACCCGCAGTGCTCCACCCCTGAGGTTCCTCATGCTGAGACCACAGTAGGTCGCCTGGTTCAGTGTGAAGCGTTGTTGGGGATTCGCTCCAGGTGTAACCCAGTGGTTCACCCACAGCCATTCATCAGCATGTGTCACACTGCCCACAAGGCTTGCGATGCCGCCCATGCTTACAGAACCATTTGCTCTCTGAGAGGAGTGGAAGAAGTTTTCCCTATGGCGTGCTAACAACACCTGTTGACATGTTAACATAGACATTAAATGTTGACGACTCATTCAGATTTTGGATATGGCTATAGGTATTAACTGTATTTTTGTTACGTAAATGCATCATAAAATGTACAAAAAAAAATCCTCAATAAAATTGCAACCAACTACATTTCTGTTTTGTATTCCATTTGCATATCTGTTTCTATATCTTTTTAAGTATCTAATTCCGGAAACCATACAGTTCTTGTAAAATTTCCCCTT

>CL2883.Contig5_All

GCCCTTGAGCTGGACCTTGGACTTTCCGGAGTAGTCTTGGTGATCGCTCAGAACCACCATGACGACCTCACAGCTCTTCTTGGTTCTCGCCCTCGTGGCAGGCAGCCTGGCAGCCCCCTGGGGAGCGGACGTGCCAAGATGCTCCACCGAATGCCCCGTCACCGGATCCCCCAAACTGGCCTACCAACCCGACAAGACCTACGCCTACCAATACTCCGGCAAGTCCAAGGTCCAGCTCAAGGGCGTGGACAACGGCGACTCGGAGACCGAGTGGACGGCACAAGTTGATCTCACCTGGATCAGCCCTTGCGACATGGCCATCTCCTTCAAGAATACCAAGGTGGATGGCACCCCCGGTCCCATCGTTGCCAGGACGCTGGAGAGACATCCACTGGTGGTGGCCGTCGTCGACGGAAGGGTGCAGCACGTGTGCGCTCACCCAGAGGACGAACCATGGGCCATCAACCTGAAAAAGGGCGTGGCTTCGGCTTTCCAGAACTCCATTCCTTCTCTGTCTGCTGTCAGCTCAGGCATCACAGTAACGGAGACTGATGTTGTGGGAAAATGCCCAACAAAGTATGAAATTGAGACCGAAGGAGAGAAAGTCATTGTCGTCAAGGAGAAGAACCACCGCCACTGTCAACAACGTTACCCAACACCCGCTCATATACCTGCACTATGGCTGAAGGCTCCCCTGCCAATCCAGGAATCCACGTCACAGTGCAAGCAGGAAATCGCCAATGGCATTTACACCGCCATCACGTGTCAGGACAAGAACATCGTTCGACCTGCCATTGGAATCTACAAGTACGTGGAGGCCAGTCAGGATTCAACACTTCGCTTCATCTCGGAGTCCTCCGACACTTCAGCTATCAGTGCCATCCCTTCAGGAGAAATGCAAGTTGAAAGCCTCCTGTACAACCACCAAACAATGAAGGACCCACAACTGGCACCTGAGCTGGATGAGCTCATGAAGGGGATCTGTGACAAGACCAAGGACACAGTTGAGGCTGAAGCTGCTGCTTTGGTTGCCAAGGCTCTCCATCTGTTACGTCGTGTTCCAGAGACAGTTGTGGTGGAGACTGCACAGAAAGTGAGACAAGGACATTACTGCAGTGACTCTGCCAGGCTGGAGAGTATCTTCTTGGACGCAGTTGCTTTCCTGCATGAGTCTGGTGCAGTAAAGGTCATGGTCCAAGAAATCGAGAATGGACGAGCAACAGGGGGACGTCTCGCTCTGTACACGGCAGCGCTCTACCTCATCCCACGACCCAGCATTGAGGCAGTCAAGGCTCTCACGCCACTCTTTGAAAGCCCTCGCCCAGTGCCCTCGGTGCTGCTGGCAGCTGCTTCCATGATAAACCACTACTGCCTTCATACTCCAGCTTGCCACCAGAAAGCTCCAGTGGCGAGAATTGCAGAGATTCTGGCCACCAGAGTCCAGAGTCACTGCTCTCCTTCTGCTGGTGCTGAGGGCGAGGAAGTACCCCTTGCATTCTTCAAGGCAATAGGGAATATGGGTGTAGCTACACCTGCCGTGACAAGGGCAGCCGTCCAATGCATTGAAGAAGAAGGACTGGAAACCAGCATTCGGGTAGCTGCAGCACAAGCCTTCAGACAAGCCAATTGCTTCCGTCCAGCAGTTGAAAAGCTAGTAGACATTGCCGTCCGACCAGCCTTTGACACCGAAGTCCGCATCGCTTCCTATCTGGCAGCTGTCCGATGTGCTGAACAGGAACACCTGGAGAAAATTATTGAGAAGATTTCAAAGGAAGAGAATACCCAAGTGCGTGGATTTGTTTTGGGTCACCTGATCAACATCCAAGAGGGTAGCTGCCCCAACAAAGAAAACCTCAGGTACCTCCTTGCCAACGTTGTCATCCCTACCGACTTCGAGAAGGACTTCAGGAAATTCTCTCGACATATAGATATGGCTTACTATGCCCCTGCCTTTGGCATGGGTGCCGGCCTCGAGTCGAACATCATCTATGCTCCCGGATCTTTCATTCCTCGTGCTGTTAACCTGAACATGAGAGCAACTGTGGATGAGACGCCCATGGACATAGCAGAGATTGGTGCGCGCTTTGAAGGAGTCGACTCCATCATTGAAGAGCTCTTGGGCCCACAGGGATACCTACGCAAAGCAACATTTGGAAAGATTATGGAGGACATTACGGGTTTTGCAGGAGAGAAAGGCCTCAAGATCATGGAGCACTTCAAGCACACAATGAGGACCAGGCGATCCATCGATGCTTCTGTCATCTCCGACTTCTTCGGCAAGCTGTATGGTGAGAGCAGTTCGCACACCCACGCCGATATATTCGCCCGGTTCATGGGGCACGAGATTACTTTCGCAGATGTTGCCCAAAGCCTCAAGGGTGTCACAGCTGACACACTCATTGAGACTTTCTTCTCTTTCTTCGAGAATTCCTTGGAACATATGAAGGATCTTAACCTGAACACAGCAAGAACTGCTCAGCTTTCCATGGATTACTCACTGCCCACCATTCAGGGCACACCACTCAAGCTGAACTTAGCTGCAACTGCTGTTGCTGGCCTCAAGATGGAGGGCAACGTCAACATTGGCCAGATCCTCTCTGACCTGGGCAATTCCCACACCGGCATCAAGGTGTTCCCAGGCCTTTCTGTACAAGCCACTGGTTTTGTTGGCTTTGAGTGCCGCTTTACCAAGGTGGGAATCGAGATGCAGAACACCATCTCTAGTGCCACTGGAGCCGCCATCAACATCAGAACAACTGAAAACAAGAAGATCGAGCTGGAATTGGAGATCCCTGACAAGATGGAACTCCTCAACATCAAGGCCGAGACTTACCTTGTCAAAGCTAGGGGAAAGAAGATGACTAAGATTTCTCCTTCCTCCATGAGAGATGTCAGGATTGAGCGCAGGTCCTGCATTGCTGCTTTGGAACCAGTATTTGGCCTCAAGGTGTGCTATGACATGAACTTCCCTGATGTGTTCCGTGCTAATGCCCTGCCACTTGGTGAACCAGCCATCGCCAAGCTGTACGTTGAGAAGGCAGATCCTTCCATGAGAGGTTACTTAGTGACTGCTGCCATCAAGAACAAGAGAGGTAACAAGCTCATTAAGATGAATGTAGAAGCAGCTGGTGCCTCAACACCAAGAAGAGCAGAAATGACCCTGTCCTACACCAAGGAAGAAGGAAGCCACATTGTTTCTGCCAAGCTTGATTCCTCCAGCATTGCTGCAGGAGTGTGGACTACTCTCACCAACGAGCAAGGACACAAGGCAGTAGAGACTTATGTCAACTTCAAATATGGTCAGACTGCTATTTCTCGAGGCATCAAGCTGGAAGCGATTGCAAGGGAAGGAAGCGTGGGAGAGGAATTCCAAGTGAACGTTTTCAGCAGCGGCACCAGGAGCTTCCCCCTCGACTCTCACATTGTGGAGGCTAAATTCATCAAGAAAACTAGTGGACCTGAATTCAATGTGGATGTGATCTGCATGACCAAAAATGCTTTAGCTGATTATTTCGACTTAAACATTGAAGTTGGAGCTGATTTCATGAGATTTTCTCCTAAAGCTCTGTATTCAACAAGATACATTCCCAAGACCCGCATTTTCTTACCTGTAAACCTGCGAAAGCTAGAAATCAATGCTGCCACTGCAGCCTGGAAAGTGACGTCGTACATTCGTGAAGGAAGTCAATCTGGCGAAAGCCGTGAGTTCAGTTCTGCTTTCAAGCTTGCCAAGGGAAGGACGGATGTCATCTTTGTACAGGCTACTCATACGATTGAAGGCAGATTCCCACAAAACGTCATCATCAAAAATGTAGCAACAGCCAAAGTTGGCAGATCATCATACAGAGCAATGTATGATGTCTTCTATCACCCTGAAAAAGTGGGAGCTTCTATTGAGGTTTTGCAGGCAGCAGGTAATGAGAAGGTTGCCCAGATAGAAGCAATTTACGAAATTTCCGGAGAGAAGCACTGCGCTAAATTCTTGGCGGCCATTCCTGGCTACATTCAACCAGTTAAAGTTGAAGCCGAGATTGAACAAGAAGCAGAAGGTCGCTACGCACTGGAGGCCGCCATCAAATATGGACCACGTACAGTACTTGGAGTGAGTGGACCAGTCCTAGCTCGTTTCACCTCCAAAGCCAACAAGCTGCAAGCCAACATCAAGCTCAGGGCAATGGCAAGTGAGCCCTACATCATTGGTGCCAATGTTGTGTTTGGCAACAAGAAACAGATGATCGCCATGGAAATCAAGGAGCGATCAGAACCTCTCATTGGTCTTGAATGGAGAATGGTCCGAGAAAGTTCCGAGAAGACCACTATTGGTGTAGTGTTTGTCCTCCCTGCCCTTATTGAGCAGAAAGTCGATGCTGAAATTACTGATGAACTTGTCCATGTTAGTTTCAACAACCTGGTTCTACCCAAGACTTCATCCCGCCGTCGAGTCAAGGGATTCGCTGATGTCAACATTGCAGAGAAAAGGGCAAATGTGGAATTTTCTTGGGATGCCGATAATGCTCCCGAAAAGAAGTTGGTGTTGGATGCAAGTCTGATCAGCAGTCCTGCCAACCCTGGACATGCTGAGATCCACGGGAATGTCGTCATTGCCGGAGAGCCTTACCACGCCAAACTGGTTCTGACTGCCACAAATCTCGTAGAGCACATGGAAGGGGAAAATGGATTCAAGTTGATCCTGACAACTCCTAGCCAGAAGACGGTTGTCGTGGGAGCCTCCTGTGATGTCCAGCTGGCAGGAGCCACCACTAAAGTCATTTCCACCGTTGAATACAAGAACGTGAGGGATAGGAAATACAAATATACAAGTGTGATTGCCTTGGAGAGGCTTGGTGGTCCACTTAATTATGCCGTAGAAGCCAAGGTAACTTACAAACAACCTGGAACAGCAGAAATAAAGGTAGAAACAACAGCAAAACATCATTGGACACCAGAAGAACATGTTGTAGCATTCAAGGTGGCTGCTGAAGCTCCAGTACTGAAGACGCCTGCCATGATTGCATTCTCCATTCACAATGCACCAAACGCTTTTGTTGGAGTCTGCAAGATCGAAAGAACTGCTCCTTTCACTGCCTTTGAATGGAATGTGCAGGTTACTCCTGAAGGAGGAATTGAAGCTGTTGAAGCTGGTGTGGACATGAAAGCCATCATTGAAGTTCTGAAGATTGTTCGTGCCATTGCTACTCTGGAGGAAGAGAGTTATGAAACTTATGGCCCACACACAGCTCAGTACCAGTACCGCTTCACAAGGCCATCACCCACTTCTTACACCATGCAGATGAGGACTCCAACCCGCACCATGGAAGGAAGAGCTAAACTATCACCAAGGGAATCTGGAATCAAGTTCTACCCCAATAAGGGCAAAACTGAATCCAAATACGAAATTGGATACAAGGTCAACCACGAGGGAAGGTGGGCACAACGTGCGTCCAAGTTGGAAGTCAGAATGAACCATCCAGTGCTTCCTAAACCCATCATGGCCGCTGCTCAGTACACAGTAGCTGAAGGAACAATGAGGGGAACAATTGAACTGGACATTTTCCCAGAAGAAGCCGACAAAATTACTGGAACTGTGGAAACTCAGAGAATTTCAGAAAATGCTATCAGGGCAGAAGTCTTCTTGACTGGCAGGATGTTGAAAGTGAACCCTAAGGCTATCATCACTGCTGCCTATGCACCAGAAACAGTTGCTTTGGATGTAGTGTTCCACAAGACTCCGTCTGCAGCACCAATCTTCGCCATTGCTGCCAAGTATGACAAGACTGCAGCTCACAGTGCAGCTGCCACATTGACAGTAAAGATGGAAGAGCGACCTGTCTTTGAGATGAGTGCAGTGACCGAACCCGAGGAACCAGCCACCTGCAATGGCATCAGAATGAATGCTGTTGCTTATGCAGCAGCTTTTGGAAAGTACAACGTGTTCTCCAAGATGTGCAGGCCCGCCTTCATTGAGGTGACCGCAATGCGACCTGGTGGAGCAAAGGAGTACACTGCCAAGCTTGGCCTCCGATACCCTGACGCTGCTGAAGCAGGCGTATATGTGGCGAGTGGCAGAGCTGGAGAGAGTCGCGGTGTTGCTGTTGCTGCTGTGAAGCTGGCTTCACCCAAAGTACTGAAAGTCGAGATGGCTTATGAGCCGCAAGAAGCAGAAGCAATAAGCATTGAAATGACTGAAGGATTTGAGAAGATTGCTGTATCATTCAAGTCTGTTGCAATGGAGTTCGTCCAATTCCTCAAGGAAGAGGCTGCTGCAAAGGGTGTTCAGTTCTCTTCATCTCAGTTAGTCAATTTAATGGGAGTTGCTAAGGAGGAAATTGTAGAGATCTATCGAGATATTCTCTCCGAGGCAAGAATTTTTCATACCGAAATCCTTGCTAATATTCTGGAAAGTCCTGTGGTATCCGTCATATCACGAGTCTACTTCGGTGTGCGGTCGGAAATTGTTCGCCTTCAACACCAGCTTTCCGTAACCCTCATCCAGGCGATAGAAGGAGGCCAGGAGGAATTAGCAATCGTTTATGAAATCGTAATGGAAGTTGTGATGACGGCAGCACGCATGGCAGAAACTGGAGAAGTCCCTGTGGCAGTGCTTGAGGCACTTGAGGAAATCAAAGCCAGCAAAGCTTTCAGGGTTGTGAAGAGAGAAGTGGATGTCATTTTGAGAGAATATCCGGAGGAGTATGAAGCTGTCAAGCACATCTTTGGCAACGTGGTGGCAATTCTCAAGCGAGATGTTGGCATTGTTCGTGAGTGGCTCATGGAGATTCCAGCTGTTCAGAGAGTCATCGACTACACCATGTATCACTTCCATTCGGAACGAGCATTTGCTGCAGAAGCAGAAAAGGTCGTTCGCCTCATTCTCGACGAACTTCTCTTCGTTTCAATGGAAAGCGAAGGCAACGGCGTTGCAGTCCGAATTCCCCTCCACCGACCCTTGTATTCACTGACGCAAGTGGCACAAGAAGCAGTGCCCAACCCTGTCACAATGCTCGAGAACCTGATATTTGCCTACCTTGAATACATTCCCATCCCTGTGAGCGACGCAATCTGGGCCTACTACAACTTCCTTCCACGCTACATCACGGACGCGCTGCCGCCCTACCCACGAACAGCCATGGTGGTTGGCGGCACTGAGATCCTCAGCTTCAGCGGCCTTGTTGTGCGAGCACCTCGCTCGCCCTGCAAGCTTCTCCTGGCTGCTCACGGCTCCCACCGCCTCATCATGTCCCACCCGCAAGCCTCAGCCCCGGCACAGCTTGAGCTCAAGACACCAGCAGCCACCGTGATCATCAAGCCTGACTTTGAAGTCCTGGTTAATGGCCAAGCCCTCGGGGGATCCCAGCAAACCATCGGAAACGTTAGGATTGTGAACACAGCCAAGCACATTGAGGTGGGATGTCCCCTGATGAGGGTGATCGTTGCCAAGGCAGGCGAGGCCGTAGCTGTTGAGGCTTCAGGCTGGATCTTTGGACGCGTAGCAGGGCTACTGGGCCCCAACACTGGAGAAATTGCCAATGACCGTCTCATGCCCAGCGGTGCAGCAGCCTCCAACCCCCGCGATTTGGTAGCTGCTTGGCAGGAGGACCCGCAGTGCTCCACCCCTGAGGTTCCTCATGCTGAGACCACAGTAGGTCGCCTGGTTCAGTGTGAAGCATTATTGGGGATTCGCTCAAGTTGTAACCCAGTGGTTCACCCACAGCCATTCATCAGCATGTGTCACACTGCCCACAAGGCTTGCGATGCCGCCCAAGCTTACAGAACCATTTGCTCTCTGAGAGGAGTGGAAGAAGTTTTCCCTATGGCGTGCTAACAACACCTGTTGACATGTTAACATAGACATTAAATGTTGACGACTCATTCGGATTTTGGATATGGCTATAGGTATTAACTGTATTTTTGTTACGTAAATGCATCATAAAATGTACAAAAAAAAATCCTCAATAAAATTGCAACCAACTACAAAA

>XM_027379601

tgacgacctcacagctcttcttggttnncgccctcgtggcaggcagcctggcagccccctggggagcggacgtgccaagatgctccaccgaatgccccgtcaccggatcccccaaactggcctaccaacccgacaagacctacgcctaccaatactccggcaagtccaaggtccagctcaagggcgtggacaacggcgactcggagaccgagtggacggcacaagttgatctcacctggatcagcccttgcgacatggccatctccttcaagaataccaaggtggatggcacccccggtcccatcgttgccaggacgctggagagacatccactggtggtggccgtcgtcgacggaagggtgcagcacgtgtgcgctcacccagaggacgaaccatgggccatcaacctgaaaaagggcgtggcttcggctttccagaactccattccttctctgtctgctgtcagctcaggcatcacagtaacggagactgatgttgtgggaaaatgcccaacaaagtatgaaattgagaccgaaggagagaaagtcattgtcgtcaaggagaagaaccaccgccactgtcaacaacgttacccancacccgctcatatacctgcactatggctgaaggctcccctgccaatccaggaatccacgtcacagtgcaagcaggaaatcgccaatggcatttacaccgccatcacgtgtcaggacaagaacatcgttcgacctgccattggaatctacaagtacgtggagnccagtcaggattcaacacttcgcttcatctcggagtcctccgacacttcagctatcagtgccatcccttcaggagaaatgcaagttgaaagcctcctgtacaaccaccaaacaatgaaggacccacaactggcacctgagctggatgagctcatgaaggggatctgtgacaagaccaaggacacagttgaggctgaagctgctgctttggttgccaaggctctccatctgttacgtcgtgttccagagacagttgtggtggagactgcacagaaagtgagacaaggacattactgcagtgactctgccaggctggagagtatcttcttggacgcagttgctttcctgcatgagtctggtgcagtaaaggtcatggtccaagaaatcgagaatggacgagcaacagggggacgtctcgctctgtacacggcagcgctctacctcatcccacgacccagcattgaggcagtcaaggctctcacgccactctttgaaagccctcgcccagtgccctcggtgctgctggcagctgcttccatgataaaccactactgccttcatactccagcttgccaccagaaagctccagtggcgagaattgcagagattctggccaccagagtccagagtcactgctctccttctgctggtgctgagggcgaggaagtaccccttgcattcttcaaggcaatagggaatatgggtgtagctacacctgccgtgacaagggcagccgtccaatgcattgaagaagaaggactggaaaccagcattcgggtagctgcagcacaagccttcagacaagccaattgcttccgtccagcagttgaaaagctagtagacattgccgtccgaccagcctttgacaccgaagtccgcatcgcttcctatctggcagctgtccgatgtgctgaacaggaacacctggagaaaattattgagaagatttcaaaggaagagaatacccaagtgcgtggatttgttttgggtcacctgatcaacatccaagagggtagctgccccaacaaagaaaacctcaggtacctccttgccaacgttgtcatccctaccgacttcgagaaggacttcaggaaattctctcgacatatagatatggcttactatgcccctgcctttggcatgggtgccggcctcgagtcgaacatcatctatgctcccggatctttcattcctcgtgctgttaacctgaacatgagagcaactgtggatgagacgcccatggacatagcagagattggtgcgcgctttgaaggagtcgattccatcattgaagagctcttgggcccacagggatacctacgcaaagcaacatttggaaagattatggaggacattacgggttttgcaggagagaaaggcctcaagatcatggagcacttcaagcacacaatgaggaccaggcgatccatcgatgcttctgtcatctccgacttcttcggcaagctgtatggtgagagcagttcgcacacccacgccgatatattcgcccggttcatgggacacgagattactttcgcagatgttgcccaaagcctcaagggcgtcacagctgacacactcattgagactttcttctctttcttcgagaattccttggaacatatgaaggatcttaacctgaacacagcaagaactgctcagctttccatggattactcactgcccaccattcagggcacaccactcaagctgaacttagctgcaactgctgttgctggcctcaagatggagggcaacgtcaacattggccagatcctctctgacctgggcaattcccacaccggcatcaaggtgttcccaggcctttctgtacaagccactggttttgttggctttgagtgccgctttaccaaggtgggaatcgagatgcagaacaccatctctagtgccactggagccgccatcaacatcagaacaactgaaaacaagaagatcgagctggaattggagatccctgacaagatggaactcctcaacatcaaggccgagacttaccttgtcaaagctaggggaaagaagatgactaagatttctccttcctccatgagagatgtcaggattgagcgcaagtcctgcattgctgctttggaaccagtatttggcctcaaggtgtgctatgacatgaacttccctgatgtgttccgtgctaatgccctgccacttggtgaaccagccatcgccaagctgtacgttgagaaggcagatccttccatgagaggttacttagtgactgctgccatcaagaacaagagaggtaacaagctcattaagatgaatgtagaagcagctggtgcctcaacaccaagaagagcagaaatgaccctgtcctacaccaaggaagaaggaagccacattgtttctgccaagcttgattcctccagcattgctgcaggagtgtggactactctcaccaacgagcaaggacacaaggcagtagagacttatgtcaacttcaaatatggtcagactgctatttctcgaggcatcaagctggaagcgattgcaagggaaggaagtgtgggagaggaattccaagtgaacgttttcagcagcggcaccaggagcttccccctcgactctcacattgtggaggctaaattcatcaagaaaactagtggacctgaattcaatgtggatgtgatctgcatgaccaaaaatgctttagctgattatttcgacttaaacattgaagttggagctgatttcatgagattttctcctaaggctctgtattcaacaagatacattcccaagacccgcattttcttacctgtaaacctgcgaaagctagaaatcaatgctgccactgcagcctggaaagtgacgtcgtacattcgtgaaggaagtcaatctggcgaaagccgtgagttcagttctgctttcaagcttgccaagggaaggacggatgtcatctttgtacaggctactcatacgattgaaggcagattcccacaaaacgtcatcatcaaaaatgtagcaacagccaaagttggcagatcatcatacagagcaatgtatgatgtcttctatcaccctgaaaaagtgggagcttctattgaggttttgcaggcagcaggtaatgagaaggttgcccagatagaagcaatttacgaaatttccggagagaagcactgcgctaaattcttggcggccattcctggctacattcaaccagttaaagttgaagccgagattgaacaagaagcagaaggtcgctacgcactggaggccgccatcaaatatggaccacgtacagtacttggagtgagtggaccagtcctagctcgtttcacctccaaagcaaagctgcaagccaacatcaagctcagggcaatggcaagtgagccctacatcattggtgccaatgttgtgtttggcaacaagaaacagatgatcgccatggaaatcaaggagcgatcagaacctctcattggtcttgaatggagaatggtccgagaaagttccgagaagaccactattggtgtagtgtttgtcctccctgcccttattgagcagaaagtcgatgctgaaattactgatgaacttgtccatgttagtttcaacaacctggttctacccaagacttcatcccgccgtcgagtcaagggattcgctgatgtcaacattgcagagaaaagggcaaatgtggagttttcttgggatgccgataatgctcccgaaaagaagttggtgttggatgcaagtctgatcagcagtcctgccaaccctggacatgctgagatccacgggaatgtcgtcattgccggagagccttaccacgccaaactggttctgactgccacaaatctcgtagagcacatggaaggggaaaatggattcaagttgatcctgacaactcctagccagaagacggttgtcgtgggagcctcctgtgatgtccagctggcaggagccaccactaaagtcatttccaccgttgaatacaagaacgtgagggataggaaatacaaatatacaagtgtgattgccttggagaggcttggtggtccacttaattatgccgtagaagccaaggtaacttacaaacaacctggaacagcagaaataaaggtagaaacaacagcaaaacatcattggacaccagaagaacatgttgtagcattcaaggtggctgctgaagctccagtactgaagacgcctgccatgattgcattctccattcacaatgcaccaaacgcttttgttggagtctgcaagatcgaaagaactgctcctttcactgcctttgaatggaatgtgcaggttactcctgaaggaggaattgaagctgttgaagctggtgtggacatgaaagccatcattgaagttctgaagattgttcgtgccattgctactctggaggaagagagttatgaaacttatggcccacacacagctcagtaccagtaccgcttcacaaggccatcacccacttcttacaccatgcagatgaggactccaacccgcaccatggaaggaagagctaaactatcaccaagggaatctggaatcaagttctaccccaataagggcaaaactgaatccaaatacgaaattggatacaaggtcaaccacgagggaaggtgggcacaacgtgcgtccaagttggaagtcagaatgaaccatccagtgcttcctaaacccatcatggccgctgctcagtacacagtagctgaaggaacaatgaggggaacaattgaactggacattttcccagaagaagccgacaaaattactggaactgtggaaactcagagaatttcagaaaatgctatcagggcagaagccttcttgactggcaggatgttgaaagtgaaccctaaagctatcatcactgctgcctatgcaccagaaacagttgctttggatgtagtgttccacaagactccatctgcagcaccaatcttcgccattgctgccaagtatgacaagactgcagctcacagtgcagctgccacattgacagtaaagatggaagagcgacctgtctttgagatgagtgcagtgaccgaacccgaggaagcagccacctgcaatggcatcagaatgaatgctgttgcttatgcagcagcttttggaaagtacaacgtgttctccaagatgtgcaggcccgccttcattgaggtgaccgcaatgcgacctggtggagcaaaggagtacactgccaagcttggcctccgataccctgacgctgctgaagcaggcgtatatgtggcgagtgctggagagagtcgcggtgttgctgttgctgctgtgaagctggcttcacccacaatgctacagttcgaggtggctcatgaaccagaagaagcacacattgtaatgagtgaagtgacaagtaccctcagaaaagtcgccatgtctctcgaaacagttgcaatggaggccgtccagttcctcaaggaagaagctgctgcaaagggtgtcgagttcccgtcatctcactttgtcagtctagtggatgaggcgaatgaggaaatcaaagccatttaccgagatatcgtctcagagatgagaatccttgacactgagttgattgctgatatcttggaaagccctacggtgtccttcgtgtcgcgtgtctaccttggagtatggtcacagattgctcgccttcaacatcacttttcaaccagggcggttgaaatgatccagcaatggcaggaacaactaacagacgtttctgaaatctttatcgaagctgttatggagatagtgcaactcttggaagccggagaagtacctgaaacagttcgtgtaattctggaaaaaattgagaacactgaggtgttcaggattgtaaagagagaagtgaacgcagtgttggcagagtatcctgaggagtatgaggccgtcaagcacatcctcaccagggtgacggccactctcaagcacgatgctgatattgtgtacaagaggatcatggagacaccagctgttcaaaggatctttgcctatgttatgcagtacatcaactcggagcgcgtgtttgctgaggaagcaggaagtgttgccagcctcattctcaaagaatttcttttcgtttcaattgaaagcgaaggcaacggcattgcagtccgaattcccctccaccgacccttgtattcactgacgcaagtggcacaagaagcagtgcccaaccctgtcacaatgctcgagaacctgatatttgcctaccttgaatacattcccatccctgtgagcgacgcaatctgggcctactacaacttccttccacgctacatcacggacgcgctgncgccctacccacgaacagccatggtggttggcggcactgagatcctcagcttcagcggccttgttgtgcgagcacctcgctcgccctgcaagcttctcctggctgctcacggctcccaccgcctcatcatgtcccacccgcaagcctcagccccggcacagcttgagctcaagacaccagcagccaccgtgatcatcaagcctgactttgaagtcctggttaatggccaagccctcnggggatcccagcaaaccatcggaaacgttaggattgtgaacacagccaagcacattgaggtgggatgtcccctgatgagggtgatcgttgccaaggcaggcgagnccgtagctgttgaggcttcaggctggatctttggacgcgtagcagggctactgggccccaacactggagaaattgccaatgaccgtctcatgcccagcggtgcagcagcctccaacccccgcgatttggtagctgcttggcaggaggacccgcagtgctccacccctgaggttcctcatgctgagaccacagtaggtcgcctggttcagtgtgaagcgttgttggggattcgctccaggtgtaacccagtggttcacccacagccattcatcagcatgtgtcacactgcccacaaggcttgcgatgccgcccatgcttacagaaccatttgctctctgagaggagtggaagaagttttccctatggcgtgctaacaacacctgttgacatgttaacatagacattaaatgttgacgactcattcagattttggatatggctataggtattaactgtatttttgttacgtgaatgcatca
t

>AY321153.2 Litopenaeus vannamei vitellogenin (Vg) mRNA, complete cds

AGTAGTCGTGGTGAGCTCGTCCAGCCAACATGACGACCTCAACTCTCCTCTTCGTTCTCGCCTTTGTGGCAGGTTGTCTGGCAGCCCCCTGGGGAGCGGACGTGCCAAGATGCTCCACCGAATGCCCCATCACCGGATCCCCCAAACTGGCCTACCAACCCGACAAGACCTACGCCTACCAATACTCCGGCAAGTCCAAGGTCCAGCTCAAGGGCGTGGACAACGGCGACTCGGAGACCGAGTGGACGGCACAAGTTGATCTCACCTGGATCAGCCCTTGCGACATGGCCATCTCCTTCAAGAATACCAAGGTGGATGGCACCCCCGGTCCCATCGTTGCCAGGACGCTGGAGAGACATCCACTGGTGGTGGCCGTCGTCGACGGAAGGGTGCAGCACGTGTGCGCTCACCCAGAGGACGAACCATGGGCCATCAACCTGAAAAAGGGCGTGGCTTCGGCTTTCCAGAACTCCATTCCTTCTCTGTCTGCTGTCAGCTCAGGCATCACAGTAACGGAGACTGATGTTGTGGGAAAATGCCCAACAAAGTATGAAATTGAGACCGAAGGAGAGAAAGTCATTGTCGTCAAGGAGAAGAACCACCGCCACTGTCAACAACGTTACCTAACACCCGCTCATATACCTGCACTATGGCTGAAGGCTCCCCTGCCAATCCAGGAATCCACGTCACAGTGCAAGCAGGAAATCGCCAATGGCATTTACACCGCCATCACGTGTCAGGACAAGAACATCGTTCGACCTGCCATTGGAATCTACAAGTACGTGGAGGCCAGTCAGGATTCAACACTTCGCTTCATCTCGGAGTCCTCCGACACTTCAGCTATCAGTGCCATCCCTTCAGGAGAAATGCAGGTTGAAAGCCTCCTGTACAACCACCAAACAATGAAGGACCCACAACTGGCACCTGAGCTGGATGAGCTCATGAAGGGGATCTGTGACAAGACCAAGGACACAGTTGAGGCTGAAGCTGCTGCTTTGGTTGCCAAGGCTCTCCATCTGTTACGTCGTGTTCCAGAGACAGTTGTGGTGGAGACTGCACAGAAAGTGAGACAAGGACATTACTGCAGTGACTCTGCCAGGCTGGAGAGTATCTTCTTGGACGCAGTTGCTTTCCTGCATGAGTCTGGTGCAGTACAGGTCATGGTCCAAGAAATCCAGAATGGACGAGCAACAGGGGGACGTCTCGCTCTGTACACGGCAGCGCTCTACCTCATCCCACGACCCAGCATTGAGGCAGTCAAGGCTCTCACGCCACTCTTTGAAAGCCCTCGCCCAGTGCCCTCGGTGCTGCTGGCAGCTGCTTCCATGATAAACCACTACTGCCTTCATACTCCAGCTTGCCACCAGAAAGCTCCAGTGGCGAGAATTGCAGAGATTCTGGCCACCAGAGTCCAGAGTCACTGCTCTCCTTCTGCTGGTGCTGAGGGCGAGGAAGTACCCCTTGCATTCTTCAAGGCAATAGGGAATATGGGTGTAGCTACACCTGCCGTGACAAGGGCAGCCGTCCAATGCATTGAAGAAGAAGGACTGGAAACCAGCATTCGGGTAGCTGCAGCACAAGCCTTCAGACAAGCCAATTGCTTCCGTCCAGCAGTTGAAAAGCTAGTAGACATTGCCGTGCGACCAGCCTTTGACACCGAAGTCCGCATCGCTTCCTATCTGGCAGCTGTCCGATGTGCTGAACAGGAACACCTGGAGAAAATTATTGAGAAGATTTCAAAGGAAGAGAATACCCAAGTGCGTGGATTTGTTTTGGGTCACCTGATCAACATCCAAGAGGGTAGCTGCCCCAACAAAGAAAACCTCAGGTACCTCCTTGCCAACGTTGTCATCCCTACCGACTTCGAGAAGGACTTCAGGAAATTCTCCCGACATATAGATATGGCTTACTATGCCCCTGCCTTTGGCATGGGTGCCGGCCTCGAGTCGAACATCATCTATGCTCCCGGATCTTTCATTCCTCGTGCTGTTAACCTGAACATGAGAGCAACTGTGGATGAGACGCCCATGGACATAGCAGAGATTGGTGCGCGCTTTGAAGGAGTCGACTCCATCATTGAAGAGCTCTTGGGCCCACAGGGATACCTACGCAAAGCAACATTTGGAAAGATTATGGAGGACATTACGGGTTTTGCAGGAGAGAAAGGCCTCAAGATCATGGAGCACATCAAGCACACAATGAGGACCAGGCGATCCATCGATGCTTCTGTCATCTCCGACTTCTTCGGCAAGCTGTATGGTGAGAGCAGTTCGCACACCCACGCCGATATATTCGCCCGGTTCATGGGACACGAGATTACTTTCGCAGATGTTGCCCAAAGCCTCAAGGGCGTCACAGCTGACACACTCATTGAGACTTTCTTCTCTTTCTTCGAGAATTCCTTGGAACATATGAAGGATCTTAACCTGAACACAGCAAGAACTGCTCAGCTTTCCATGGATTACTCACTGCCCACCATTCAGGGCACACCACTCAAGCTGAACTTAGCTGCAACTGCTGTTGCTGGCCTCAAGATGGAGGGCAACGTCAACATTGGCCAGATCCTCTCTGACCTGGGCAATTCCCACACCGGCATCAAGGTGTTCCCAGGCCTTTCTGTACAAGCCACTGGTTTTGTTGGCTTTGAGTGCCGCTTTACCAAGGTGGGAATCGAGATGCAGAACACCATCTCTAGTGCCACTGGAGCCGCCATCAACATCAGAACAACTGAAAACAAGAAGATCGAGCTGGAATTGGAGATCCCTGACAAGATGGAACTCCTCAACATCAAGGCCGAGACTTACCTTGTCAAAGCTAGGGGAAAGAAGATGACTAAGATTTCTCCTTCCTCCATGAGAGATGTCAGGATTGAGCGCAAGTCCTGCATTGCTGCTTTGGAACCAGTATTTGGCCTCAAGGTGTGCTATGACATGAACTTCCCTGATGTGTTCCGTGCTAATGCCCTGCCACTTGGTGAACCAGCCATCGCCAAGCTGTACGTTGAGAAGGCAGATCCTTCCATGAGAGGTTACTTAGTGACTGCTGCCATCAAGAACAAGAGAGGTAACAAGCTCATTAAGATGAATGTAGAAGCAGCTGGTGCCTCAACACCAAGAAGAGCAGAAATGACCCTGTCCTACACCAAGGAAGAAGGAAGCCACATTGTTTCTGCCAAGCTTGATTCCTCCAGCATTGCTGCAGGAGTGTGGACCACTCTCATCAACGAGCAAGGACACAAGGCAGTAGAGACTTATGTCAACTTCAAATATGGTCAGACTGCTATTTCTCGAGGCATCAAGCTGGAAGCGATTGCAAGGGAAGGAAGTGTGGGAGAGGAATTCCAAGTGAACGTTTTCAGCAGCGGCACCAGGAGCTTCCCCCTCGACTCTCACATTGTGGAGGCTAAATTCATCAAGAAAACTAGTGGACCTGAATTCAATGTGGATGTGATCTGCATGACCAAAAATGCTTTAGCTGATTATTTCGACTTAAACATTGAAGTTGGAGCTGATTTCATGAGATTTTCTCCTAAAGCTCTGTATTCAACAAGATACATTCCCAAGACCCGCATTTTCTTACCTGTAAACCTGCGAAAGCTAGAAATCAATGCTGCCACTGCAGCCTGGAAAGTGACGTCGTACATTCGTGAAGGAAGTCAATCTGGCGAAAGCCGTGAGTTCAGTTCTGCTTTCAAGCTTGCCAAGGGAAGGACGGATGTCATCTTTGTACAGGCTACTCATACGATTGAAGGCAGATTCCCACAAAACGTCATCATCAAAAATGTAGCAACAGCCAAAGTTGGCAGATCATCATACAGAGCAATGTATGATGTCTTCTATCACCCTGAAAAAGTGGGAGCTTCTATTGAGGTTTTGCAGGCAGCAGGTAATGAGAAGGTTGCCCAGATAGAAGCAATTTACGAAATTTCCGGAGAGAAGCACTGCGCTAAATTCTTGGCGGCCATTCCTGGCTACATTCAACCAGTTAAAGTTGAAGCCGAGATTGAACAAGAAGCAGAAGGTCGCTACGCACTGGAGTCCGCCATCAAATATGGACCACGTACAGTACTTGGAGTGAGTGGACCAGTCCTAGCTCGTTTCACCTCCAAAGCCAACAAGCTGCAAGCCAACATCAAGCTCAGGGCAATGGCAAGTGAGCCCTACATCATTGGTGCCAATGTTGTGTTTGGCAACAAGAAACAGATGATCGCCATGGAAATCAAGGAGCGATCAGAACCTCTCATTGGTCTTGAATGGAGAATGGTCCGAGAAAGTTCCGAGAAGACCACTATTGGTGTAGTGTTTGTCCTCCCTGCCCTTATTGAGCAGAAAGTCGATGCTGAAATTACTGATGAACTTGTCCATGTTAGTTTCAACAACCTGGTTCTACCCAAGACTTCATCCCGCCGTCGAGTCAAGGGATTCGCTGATGTCAACATTGCAGAGAAAAGGGCAAATGTGGAGTTTTCTTGGGATGCCGATAATGCTCCCGAAAAGAAGTTGGTGTTGGATGCAAGTCTGATCAGCAGTCCTGCCAACCCTGGACATGCTGAGATCCACGGGAATGTCGTCATTGCCGGAGAGCCTTACCACGCCAAACTGGTTCTGACTGCCACAAATCTCGTAGAGCACATGGAAGGGGAAAATGGATTCAAGTTGATCCTGACAACTCCTAGCCAGAAGACGGTTGTCGTGGGAGCCTCCTGTGATGTCCAGCTGGCAGGAACCACCACTAAAGTCCTTTCCACCGTTGAATACAAGAACGTGAGGGATAGGAAATACAAATATACAAGTGTGATTGCCTTGGAGAGGCTTGGTGGTCCACTTAATTATGCCGTAGAAGCCAAGGTAACTTACAAACAACCTGGAACAGCAGAAATAAAGGTAGAAACAACAGCAAAACATCATTGGACACCAGAAGAACATGTTGTAGCATTCAAGGTGGCTGCTGAAGCTCCAGTACTGAAGACGCCTGCCATGATTGCATTCTCCATTCACAATGCACCAAACGCTTTTGTTGGAGTCTGCAAGATCGAAAGAACTGCTCCTTTCACTGCCTTTGAATGGAATGTACAGGTTACTCCTGAAGGAGGAATTGAAGCTGTTGAAGCTGGTGTGGACATGAAAGCCATCATTGAAGTTCTGAAGATTGTTCGTGCCATTGCTACTCTGGAGGAAGAGAGTTATGAAACTTATGGCCCACACACAGCTCAGTACCAGTACCGCTTCACAAGGCCATCACCCACTTCTTACACCATGCAGATGAGGACTCCAACCCGCACCATGGAAGGAAGAGCTAAACTATCACCAAGGGAATCTGGAATCAAGTTCTACCCCAATAAGGGCAAAACTGAATCCAAATACGAAATTGGATACAAGGTCAACCACGAGGGAAGGTGGGGACAACGTGCGTCCAAGTTGGAAGTCAGAATGAACCATCCAGTGCTTCCTAAACCCATCATGGCCGCTGCTCAGTACACAGTAGCTGAAGGAACAATGAGGGGAACAATTGAACTGGACATTTTCCCAGAAGAAGCCGACAAAATTACTGGAACTGTGGAAACTCAGAGAATTTCAGAAAATGCTATCAGGGCAGAAGTCTTCTTGACTGGCAGGATGTTGAAAGTGAACCCTAAGGCTATCATCACTGCTGCCTATGCACCAGAAACAGTTGCTTTGGATGTAGTGTTCCACAAGACTCCGTCTGCAGCACCAATCTTCGCCATTGCTGCCAAGTATGACAAGACTGCAGCTCACAGTGCAGCTGCCACATTGACAGTAAAGATGGAAGAGCGACCTGTCTTTGAGATGAGTGCAGTGACCGAACCTGAGGAAGCAGCCACCTGCAATGGCATCAGAATGAATGCTGTTGCTTATGCAGCAGCTTTTGGAAAGTACAACGTGTTCTCCAAGATGTGCAGGCCCGCCTTCATTGAGGTGACCGCAATGCGACCTGGTGGAGCAAAGGAGTACACTGCCAAGCTTGGCCTCCGATACCCTGACGCTGCTGAAGCAGGCGTATATGTGACGAGTGGCAGAGCTGGAGAGAGTCGCGGTGTTGCTGTTGCTGCTGTGAAGCTGGCTTCACCCAAAGTACTGAAAGTCGAGATGGCTTATGAGCCGCAAGAAGCAGAAGCAATAAGCATTGAAATGACTGAAGGATTTGAGAAGATTGCTGTATCATTCAAGTCTGTTGCAATAGAGTTCGTCCAATTCCTCAAGGAAGAGGCTGCTGCAAAGGGTGTTCAGTTCTCTTCATCTCAGTTAGTCAATCTAATGGGAGTTGCTAAGGAGGAAATTGTAGAGATCTATCGAGATATTCTCTCCGAGGCAAGAATTTTTCATACCGAAATCCTTGCTAATATTCTGGAAAGTCCTGTGGTATCCGTCATATCACGAGTCTACTTCGGTGTGCGGTCGGAAATTGTTCGCCTTCAACACCAGCTTTCCGTAACCCTCATCCAGGCGATAGAAGGAGGCCAAGAGGAATTAGCAATCGTTTATGAAATCGTAATGGAAGTTGTGATGACGGCAGCACGCATGGCAGAAACTGGAGAAGTCCCTGTGGCAGTGCTTGAGGCACTTGAGGAAATCAAAGCCAGCAAAGCTTTCAGGGTTGTGAAGAGAGAAGTGGATGTCATTTTGAGAGAATATCCGGAGGAGTATGAAGCTGTCAAGCACATCTTTGGCAACGTGGTGGCAATTCTCAAGCGAGATGTTGGCATTGTTCGTGAGTGGCTCATGGAGATTCCAGCTGTTCAGAGAGTCATCGACTACACCATGTATCACTTCCATTCGGAACGAGCATTTGCTGCAGAAGCAGAAAAGGTCGTTCGCCTCATTCTCGACGAACTTCTCTTCGTTTCAATGGAAAGCGAAGGCAACGGCGTTGCAGTCCGAATTCCCCTCCACCGACCCTTTTATTCACTAACGCAAGTGGCACAAGAAGCAGTGCCCAGCCCTGTCACAATGCTCGAGAACCTGATATTTGCCTACCTTGAATACATTCCCATCCCTGTGAGCGACGCAATCTGGGCCTACTACAACTTCCTTCCACGCTACATCACGGACGCGCTGCCGCCCTACCCACGAACAGCCATGGTGGTTGGCGGCACTGAGATCCTCAGCTTCAGCGGCCTTGTTGTGCGAGCACCTCGCTCGCCCTGCAAGCTTCTCCTGGCTGCTCACGGCTCCCACCGCCTCATCATGTCCCACCCGCAAGCCTCAGCCCCGGCACAGCTTGAGCTCAAGACACCAGCAGCCACCGTGATCATCAAGCCTGACTTTGAAGTCCTGGTTAATGGCCAAGCCCTCGGGGGATCCCAGCAAACCATCGGAAACGTTAGGATTGTGAACACAGCCAAGCACATTGAGGTGGGATGTCCCCTGATGAGGGTGATCGTTGCCAAGGCAGGCGAGGCCGTAGCTGTTGAGGCTTCAGGCTGGATCTTTGGACGCGTAGCAGGGCTACTGGGCCCCAACACTGGAGAAATTGCCAATGACCGTCTCATGCCCAGCGGTGCAGCAGCCTCCAACCCCCGCGATTTGGTAGCTGCTTGGCAGGAGGACCCGCAGTGCTCCACCCCTGAGGTTCCTCATGCTGAGACCACAGTAGGTCGCCTGGTTCAGTGTGAAGCGTTGTTGGGGATTCGCTCCAGGTGTAACCCAGTGGTTCACCCACAGCCATTCATCAGCATGTGTCACACTGCCCACAAGGCTTGCGATGCCGCCCATGCTTACAGAACCATTTGCTCTCTGAGAGGAGTGGAAGAAGTTTTCCCTATGGCGTGCTAACAACACCTGTTGACATGTTAACATAGACATTAAATGTTGACGACTCATTCAGATTTTGGATATGGCTATAGGTATTAACTGTATTTTTGTTACGTAAATGCATCATAAAATGTACAAAAAAAAATCCTCAATAAAATTGCAACCAACTAAAAAAAAAAAAAAAAAAAAAAAAAAAAA

[clustalw.aln](https://www.genome.jp/tools-bin/pushfile?200823083600qN8b1+clustalw.aln)

CLUSTAL 2.1 multiple sequence alignment

CL2883.CONTIG1_ALL -------ACCTGCCACAAAGGCGAGAACGAGTAGTCGTGGTGAGCTCGTCCAGCCAACAT

CL2883.CONTIG4_ALL -----------------------CCGGGGAGTAGTCTTGGTGATCGCTCAGAACCACCAT

CL2883.CONTIG5_ALL GCCCTTGAGCTGGACCTTGGACTTTCCGGAGTAGTCTTGGTGATCGCTCAGAACCACCAT

XM_027379601 -----------------------------------------------------------T

AY321153.2 -----------------------------AGTAGTCGTGGTGAGCTCGTCCAGCCAACAT

CL2833.CONTIG3 ------------------------------------------------------------

CL2883.CONTIG2_ALL -------ACCTGCCACAAAGGCGAGAACGAGTAGTCGTGGTGAGCTCGTCCAGCCAACAT

CL2883.CONTIG1_ALL GACGACCTCAACTCTCCTCTTCGTTCTCGCCTTTGTGGCAGGTTGTCTGGCAGCCCCCTG

CL2883.CONTIG4_ALL GACGACCTCACAGCTCTTCTTGGTTCTCGCCCTCGTGGCAGGCAGCCTGGCAGCCCCCTG

CL2883.CONTIG5_ALL GACGACCTCACAGCTCTTCTTGGTTCTCGCCCTCGTGGCAGGCAGCCTGGCAGCCCCCTG

XM_027379601 GACGACCTCACAGCTCTTCTTGGTTNNCGCCCTCGTGGCAGGCAGCCTGGCAGCCCCCTG

AY321153.2 GACGACCTCAACTCTCCTCTTCGTTCTCGCCTTTGTGGCAGGTTGTCTGGCAGCCCCCTG

CL2833.CONTIG3 ------------------------------------------------------------

CL2883.CONTIG2_ALL GACGACCTCAACTCTCCTCTTCGTTCTCGCCTTTGTGGCAGGTTGTCTGGCAGCCCCCTG

CL2883.CONTIG1_ALL GGGAGCGGACGTGCCAAGATGCTCCACCGAATGCCCCGTCACCGGATCCCCCAAACTGGC

CL2883.CONTIG4_ALL GGGAGCGGACGTGCCAAGATGCTCCACCGAATGCCCCGTCACCGGATCCCCCAAACTGGC

CL2883.CONTIG5_ALL GGGAGCGGACGTGCCAAGATGCTCCACCGAATGCCCCGTCACCGGATCCCCCAAACTGGC

XM_027379601 GGGAGCGGACGTGCCAAGATGCTCCACCGAATGCCCCGTCACCGGATCCCCCAAACTGGC

AY321153.2 GGGAGCGGACGTGCCAAGATGCTCCACCGAATGCCCCATCACCGGATCCCCCAAACTGGC

CL2833.CONTIG3 -------------CCAAGATGCTCCACCGAATGCCCCGTCACCGGATCCCCCAAACTGGC

CL2883.CONTIG2_ALL GGGAGCGGACGTGCCAAGATGCTCCACCGAATGCCCCGTCACCGGATCCCCCAAACTGGC

************************.**********************

CL2883.CONTIG1_ALL CTACCAACCCGACAAGACCTACGCCTACCAATACTCCGGCAAGTCCAAGGTCCAGCTCAA

CL2883.CONTIG4_ALL CTACCAACCCGACAAGACCTACGCCTACCAATACTCCGGCAAGTCCAAGGTCCAGCTCAA

CL2883.CONTIG5_ALL CTACCAACCCGACAAGACCTACGCCTACCAATACTCCGGCAAGTCCAAGGTCCAGCTCAA

XM_027379601 CTACCAACCCGACAAGACCTACGCCTACCAATACTCCGGCAAGTCCAAGGTCCAGCTCAA

AY321153.2 CTACCAACCCGACAAGACCTACGCCTACCAATACTCCGGCAAGTCCAAGGTCCAGCTCAA

CL2833.CONTIG3 CTACCAACCCGACAAGACCTACGCCTACCAATACTCCGGCAAGTCCAAGGTCCAGCTCAA

CL2883.CONTIG2_ALL CTACCAACCCGACAAGACCTACGCCTACCAATACTCCGGCAAGTCCAAGGTCCAGCTCAA

************************************************************

CL2883.CONTIG1_ALL GGGCGTGGACAACGGCGACTCGGAGACCGAGTGGACGGCACAAGTTGATCTCACCTGGAT

CL2883.CONTIG4_ALL GGGCGTGGACAACGGCGACTCGGAGACCGAGTGGACGGCACAAGTTGATCTCACCTGGAT

CL2883.CONTIG5_ALL GGGCGTGGACAACGGCGACTCGGAGACCGAGTGGACGGCACAAGTTGATCTCACCTGGAT

XM_027379601 GGGCGTGGACAACGGCGACTCGGAGACCGAGTGGACGGCACAAGTTGATCTCACCTGGAT

AY321153.2 GGGCGTGGACAACGGCGACTCGGAGACCGAGTGGACGGCACAAGTTGATCTCACCTGGAT

CL2833.CONTIG3 GGGCGTGGACAACGGCGACTCGGAGACCGAGTGGACGGCACAAGTTGATCTCACCTGGAT

CL2883.CONTIG2_ALL GGGCGTGGACAACGGCGACTCGGAGACCGAGTGGACGGCACAAGTTGATCTCACCTGGAT

************************************************************

CL2883.CONTIG1_ALL CAGCCCTTGCGACATGGCCATCTCCTTCAAGAATACCAAGGTGGATGGCACCCCCGGTCC

CL2883.CONTIG4_ALL CAGCCCTTGCGACATGGCCATCTCCTTCAAGAATACCAAGGTGGATGGCACCCCCGGTCC

CL2883.CONTIG5_ALL CAGCCCTTGCGACATGGCCATCTCCTTCAAGAATACCAAGGTGGATGGCACCCCCGGTCC

XM_027379601 CAGCCCTTGCGACATGGCCATCTCCTTCAAGAATACCAAGGTGGATGGCACCCCCGGTCC

AY321153.2 CAGCCCTTGCGACATGGCCATCTCCTTCAAGAATACCAAGGTGGATGGCACCCCCGGTCC

CL2833.CONTIG3 CAGCCCTTGCGACATGGCCATCTCCTTCAAGAATACCAAGGTGGATGGCACCCCCGGTCC

CL2883.CONTIG2_ALL CAGCCCTTGCGACATGGCCATCTCCTTCAAGAATACCAAGGTGGATGGCACCCCCGGTCC

************************************************************

CL2883.CONTIG1_ALL CATCGTTGCCAGGACGCTGGAGAGACATCCACTGGTGGTGGCCGTCGTCGACGGAAGGGT

CL2883.CONTIG4_ALL CATCGTTGCCAGGACGCTGGAGAGACATCCACTGGTGGTGGCCGTCGTCGACGGAAGGGT

CL2883.CONTIG5_ALL CATCGTTGCCAGGACGCTGGAGAGACATCCACTGGTGGTGGCCGTCGTCGACGGAAGGGT

XM_027379601 CATCGTTGCCAGGACGCTGGAGAGACATCCACTGGTGGTGGCCGTCGTCGACGGAAGGGT

AY321153.2 CATCGTTGCCAGGACGCTGGAGAGACATCCACTGGTGGTGGCCGTCGTCGACGGAAGGGT

CL2833.CONTIG3 CATCGTTGCCAGGACGCTGGAGAGACATCCACTGGTGGTGGCCGTCGTCGACGGAAGGGT

CL2883.CONTIG2_ALL CATCGTTGCCAGGACGCTGGAGAGACATCCACTGGTGGTGGCCGTCGTCGACGGAAGGGT

************************************************************

CL2883.CONTIG1_ALL GCAGCACGTGTGCGCTCACCCAGAGGACGAACCATGGGCCATCAACCTGAAAAAGGGCGT

CL2883.CONTIG4_ALL GCAGCACGTGTGCGCTCACCCAGAGGACGAACCATGGGCCATCAACCTGAAAAAGGGCGT

CL2883.CONTIG5_ALL GCAGCACGTGTGCGCTCACCCAGAGGACGAACCATGGGCCATCAACCTGAAAAAGGGCGT

XM_027379601 GCAGCACGTGTGCGCTCACCCAGAGGACGAACCATGGGCCATCAACCTGAAAAAGGGCGT

AY321153.2 GCAGCACGTGTGCGCTCACCCAGAGGACGAACCATGGGCCATCAACCTGAAAAAGGGCGT

CL2833.CONTIG3 GCAGCACGTGTGCGCTCACCCAGAGGACGAACCATGGGCCATCAACCTGAAAAAGGGCGT

CL2883.CONTIG2_ALL GCAGCACGTGTGCGCTCACCCAGAGGACGAACCATGGGCCATCAACCTGAAAAAGGGCGT

************************************************************

CL2883.CONTIG1_ALL GGCTTCGGCTTTCCAGAACTCCATTCCTTCTCTGTCTGCTGTCAGCTCAGGCATCACAGT

CL2883.CONTIG4_ALL GGCTTCGGCTTTCCAGAACTCCATTCCTTCTCTGTCTGCTGTCAGCTCAGGCATCACAGT

CL2883.CONTIG5_ALL GGCTTCGGCTTTCCAGAACTCCATTCCTTCTCTGTCTGCTGTCAGCTCAGGCATCACAGT

XM_027379601 GGCTTCGGCTTTCCAGAACTCCATTCCTTCTCTGTCTGCTGTCAGCTCAGGCATCACAGT

AY321153.2 GGCTTCGGCTTTCCAGAACTCCATTCCTTCTCTGTCTGCTGTCAGCTCAGGCATCACAGT

CL2833.CONTIG3 GGCTTCGGCTTTCCAGAACTCCATTCCTTCTCTGTCTGCTGTCAGCTCAGGCATCACAGT

CL2883.CONTIG2_ALL GGCTTCGGCTTTCCAGAACTCCATTCCTTCTCTGTCTGCTGTCAGCTCAGGCATCACAGT

************************************************************

CL2883.CONTIG1_ALL AACGGAGACTGATGTTGTGGGAAAATGCCCAACAAAGTATGAAATTGAGACCGAAGGAGA

CL2883.CONTIG4_ALL AACGGAGACTGATGTTGTGGGAAAATGCCCAACAAAGTATGAAATTGAGACCGAAGGAGA

CL2883.CONTIG5_ALL AACGGAGACTGATGTTGTGGGAAAATGCCCAACAAAGTATGAAATTGAGACCGAAGGAGA

XM_027379601 AACGGAGACTGATGTTGTGGGAAAATGCCCAACAAAGTATGAAATTGAGACCGAAGGAGA

AY321153.2 AACGGAGACTGATGTTGTGGGAAAATGCCCAACAAAGTATGAAATTGAGACCGAAGGAGA

CL2833.CONTIG3 AACGGAGACTGATGTTGTGGGAAAATGCCCAACAAAGTATGAAATTGAGACCGAAGGAGA

CL2883.CONTIG2_ALL AACGGAGACTGATGTTGTGGGAAAATGCCCAACAAAGTATGAAATTGAGACCGAAGGAGA

************************************************************

CL2883.CONTIG1_ALL GAAAGTCATTGTCGTCAAGGAGAAGAACCACCGCCACTGTCAACAACGTTACCCAACACC

CL2883.CONTIG4_ALL GAAAGTCATTGTCGTCAAGGAGAAGAACCACCGCCACTGTCAACAACGTTACCCAACACC

CL2883.CONTIG5_ALL GAAAGTCATTGTCGTCAAGGAGAAGAACCACCGCCACTGTCAACAACGTTACCCAACACC

XM_027379601 GAAAGTCATTGTCGTCAAGGAGAAGAACCACCGCCACTGTCAACAACGTTACCCANCACC

AY321153.2 GAAAGTCATTGTCGTCAAGGAGAAGAACCACCGCCACTGTCAACAACGTTACCTAACACC

CL2833.CONTIG3 GAAAGTCATTGTCGTCAAGGAGAAGAACCACCGCCACTGTCAACAACGTTACCCAACACC

CL2883.CONTIG2_ALL GAAAGTCATTGTCGTCAAGGAGAAGAACCACCGCCACTGTCAACAACGTTACCCAACACC

***************************************************** * ****

CL2883.CONTIG1_ALL CGCTCATATACCTGCACTATGGCTGAAGGCTCCCCTGCCAATCCAGGAATCCACGTCACA

CL2883.CONTIG4_ALL CGCTCATATACCTGCACTATGGCTGAAGGCTCCCCTGCCAATCCAGGAATCCACGTCACA

CL2883.CONTIG5_ALL CGCTCATATACCTGCACTATGGCTGAAGGCTCCCCTGCCAATCCAGGAATCCACGTCACA

XM_027379601 CGCTCATATACCTGCACTATGGCTGAAGGCTCCCCTGCCAATCCAGGAATCCACGTCACA

AY321153.2 CGCTCATATACCTGCACTATGGCTGAAGGCTCCCCTGCCAATCCAGGAATCCACGTCACA

CL2833.CONTIG3 CGCTCATATACCTGCACTATGGCTGAAGGCTCCCCTGCCAATCCAGGAATCCACGTCACA

CL2883.CONTIG2_ALL CGCTCATATACCTGCACTATGGCTGAAGGCTCCCCTGCCAATCCAGGAATCCACGTCACA

************************************************************

CL2883.CONTIG1_ALL GTGCAAGCAGGAAATCGCCAATGGCATTTACACCGCCATCACGTGTCAGGACAAGAACAT

CL2883.CONTIG4_ALL GTGCAAGCAGGAAATCGCCAATGGCATTTACACCGCCATCACGTGTCAGGACAAGAACAT

CL2883.CONTIG5_ALL GTGCAAGCAGGAAATCGCCAATGGCATTTACACCGCCATCACGTGTCAGGACAAGAACAT

XM_027379601 GTGCAAGCAGGAAATCGCCAATGGCATTTACACCGCCATCACGTGTCAGGACAAGAACAT

AY321153.2 GTGCAAGCAGGAAATCGCCAATGGCATTTACACCGCCATCACGTGTCAGGACAAGAACAT

CL2833.CONTIG3 GTGCAAGCAGGAAATCGCCAATGGCATTTACACCGCCATCACGTGTCAGGACAAGAACAT

CL2883.CONTIG2_ALL GTGCAAGCAGGAAATCGCCAATGGCATTTACACCGCCATCACGTGTCAGGACAAGAACAT

************************************************************

CL2883.CONTIG1_ALL CGTTCGACCTGCCATTGGAATCTACAAGTACGTGGAGGCCAGTCAGGATTCAACACTTCG

CL2883.CONTIG4_ALL CGTTCGACCTGCCATTGGAATCTACAAGTACGTGGAGGCCAGTCAGGATTCAACACTTCG

CL2883.CONTIG5_ALL CGTTCGACCTGCCATTGGAATCTACAAGTACGTGGAGGCCAGTCAGGATTCAACACTTCG

XM_027379601 CGTTCGACCTGCCATTGGAATCTACAAGTACGTGGAGNCCAGTCAGGATTCAACACTTCG

AY321153.2 CGTTCGACCTGCCATTGGAATCTACAAGTACGTGGAGGCCAGTCAGGATTCAACACTTCG

CL2833.CONTIG3 CGTTCGACCTGCCATTGGAATCTACAAGTACGTGGAGGCCAGTCAGGATTCAACACTTCG

CL2883.CONTIG2_ALL CGTTCGACCTGCCATTGGAATCTACAAGTACGTGGAGGCCAGTCAGGATTCAACACTTCG

*************************************.**********************

CL2883.CONTIG1_ALL CTTCATCTCGGAGTCCTCCGACACTTCAGCTATCAGTGCCATCCCTTCAGGAGAAATGCA

CL2883.CONTIG4_ALL CTTCATCTCGGAGTCCTCCGACACTTCAGCTATCAGTGCCATCCCTTCAGGAGAAATGCA

CL2883.CONTIG5_ALL CTTCATCTCGGAGTCCTCCGACACTTCAGCTATCAGTGCCATCCCTTCAGGAGAAATGCA

XM_027379601 CTTCATCTCGGAGTCCTCCGACACTTCAGCTATCAGTGCCATCCCTTCAGGAGAAATGCA

AY321153.2 CTTCATCTCGGAGTCCTCCGACACTTCAGCTATCAGTGCCATCCCTTCAGGAGAAATGCA

CL2833.CONTIG3 CTTCATCTCGGAGTCCTCCGACACTTCAGCTATCAGTGCCATCCCTTCAGGAGAAATGCA

CL2883.CONTIG2_ALL CTTCATCTCGGAGTCCTCCGACACTTCAGCTATCAGTGCCATCCCTTCAGGAGAAATGCA

************************************************************

CL2883.CONTIG1_ALL AGTTGAAAGCCTCCTGTACAACCACCAAACAATGAAGGACCCACAACTGGCACCTGAGCT

CL2883.CONTIG4_ALL AGTTGAAAGCCTCCTGTACAACCACCAAACAATGAAGGACCCACAACTGGCACCTGAGCT

CL2883.CONTIG5_ALL AGTTGAAAGCCTCCTGTACAACCACCAAACAATGAAGGACCCACAACTGGCACCTGAGCT

XM_027379601 AGTTGAAAGCCTCCTGTACAACCACCAAACAATGAAGGACCCACAACTGGCACCTGAGCT

AY321153.2 GGTTGAAAGCCTCCTGTACAACCACCAAACAATGAAGGACCCACAACTGGCACCTGAGCT

CL2833.CONTIG3 AGTTGAAAGCCTCCTGTACAACCACCAAACAATGAAGGACCCACAACTGGCACCTGAGCT

CL2883.CONTIG2_ALL AGTTGAAAGCCTCCTGTACAACCACCAAACAATGAAGGACCCACAACTGGCACCTGAGCT

.***********************************************************

CL2883.CONTIG1_ALL GGATGAGCTCATGAAGGGGATCTGTGACAAGACCAAGGACACAGTTGAGGCTGAAGCTGC

CL2883.CONTIG4_ALL GGATGAGCTCATGAAGGGGATCTGTGACAAGACCAAGGACACAGTTGAGGCTGAAGCTGC

CL2883.CONTIG5_ALL GGATGAGCTCATGAAGGGGATCTGTGACAAGACCAAGGACACAGTTGAGGCTGAAGCTGC

XM_027379601 GGATGAGCTCATGAAGGGGATCTGTGACAAGACCAAGGACACAGTTGAGGCTGAAGCTGC

AY321153.2 GGATGAGCTCATGAAGGGGATCTGTGACAAGACCAAGGACACAGTTGAGGCTGAAGCTGC

CL2833.CONTIG3 GGATGAGCTCATGAAGGGGATCTGTGACAAGACCAAGGACACAGTTGAGGCTGAAGCTGC

CL2883.CONTIG2_ALL GGATGAGCTCATGAAGGGGATCTGTGACAAGACCAAGGACACAGTTGAGGCTGAAGCTGC

************************************************************

CL2883.CONTIG1_ALL TGCTTTGGTTGCCAAGGCTCTCCATCTGTTACGTCGTGTTCCAGAGACAGTTGTGGTGGA

CL2883.CONTIG4_ALL TGCTTTGGTTGCCAAGGCTCTCCATCTGTTACGTCGTGTTCCAGAGACAGTTGTGGTGGA

CL2883.CONTIG5_ALL TGCTTTGGTTGCCAAGGCTCTCCATCTGTTACGTCGTGTTCCAGAGACAGTTGTGGTGGA

XM_027379601 TGCTTTGGTTGCCAAGGCTCTCCATCTGTTACGTCGTGTTCCAGAGACAGTTGTGGTGGA

AY321153.2 TGCTTTGGTTGCCAAGGCTCTCCATCTGTTACGTCGTGTTCCAGAGACAGTTGTGGTGGA

CL2833.CONTIG3 TGCTTTGGTTGCCAAGGCTCTCCATCTGTTACGTCGTGTTCCAGAGACAGTTGTGGTGGA

CL2883.CONTIG2_ALL TGCTTTGGTTGCCAAGGCTCTCCATCTGTTACGTCGTGTTCCAGAGACAGTTGTGGTGGA

************************************************************

CL2883.CONTIG1_ALL GACTGCACAGAAAGTGAGACAAGGACATTACTGCAGTGACTCTGCCAGGCTGGAGAGTAT

CL2883.CONTIG4_ALL GACTGCACAGAAAGTGAGACAAGGACATTACTGCAGTGACTCTGCCAGGCTGGAGAGTAT

CL2883.CONTIG5_ALL GACTGCACAGAAAGTGAGACAAGGACATTACTGCAGTGACTCTGCCAGGCTGGAGAGTAT

XM_027379601 GACTGCACAGAAAGTGAGACAAGGACATTACTGCAGTGACTCTGCCAGGCTGGAGAGTAT

AY321153.2 GACTGCACAGAAAGTGAGACAAGGACATTACTGCAGTGACTCTGCCAGGCTGGAGAGTAT

CL2833.CONTIG3 GACTGCACAGAAAGTGAGACAAGGACATTACTGCAGTGACTCTGCCAGGCTGGAGAGTAT

CL2883.CONTIG2_ALL GACTGCACAGAAAGTGAGACAAGGACATTACTGCAGTGACTCTGCCAGGCTGGAGAGTAT

************************************************************

CL2883.CONTIG1_ALL CTTCTTGGACGCAGTTGCTTTCCTGCATGAGTCTGGTGCAGTAAAGGTCATGGTCCAAGA

CL2883.CONTIG4_ALL CTTCTTGGACGCAGTTGCTTTCCTGCATGAGTCTGGTGCAGTAAAGGTCATGGTCCAAGA

CL2883.CONTIG5_ALL CTTCTTGGACGCAGTTGCTTTCCTGCATGAGTCTGGTGCAGTAAAGGTCATGGTCCAAGA

XM_027379601 CTTCTTGGACGCAGTTGCTTTCCTGCATGAGTCTGGTGCAGTAAAGGTCATGGTCCAAGA

AY321153.2 CTTCTTGGACGCAGTTGCTTTCCTGCATGAGTCTGGTGCAGTACAGGTCATGGTCCAAGA

CL2833.CONTIG3 CTTCTTGGACGCAGTTGCTTTCCTGCATGAGTCTGGTGCAGTAAAGGTCATGGTCCAAGA

CL2883.CONTIG2_ALL CTTCTTGGACGCAGTTGCTTTCCTGCATGAGTCTGGTGCAGTACAGGTCATGGTCCAAGA

*******************************************.****************

CL2883.CONTIG1_ALL AATCGAGAATGGACGAGCAACAGGGGGACGTCTCGCTCTGTACACGGCAGCGCTCTACCT

CL2883.CONTIG4_ALL AATCGAGAATGGACGAGCAACAGGGGGACGTCTCGCTCTGTACACGGCAGCGCTCTACCT

CL2883.CONTIG5_ALL AATCGAGAATGGACGAGCAACAGGGGGACGTCTCGCTCTGTACACGGCAGCGCTCTACCT

XM_027379601 AATCGAGAATGGACGAGCAACAGGGGGACGTCTCGCTCTGTACACGGCAGCGCTCTACCT

AY321153.2 AATCCAGAATGGACGAGCAACAGGGGGACGTCTCGCTCTGTACACGGCAGCGCTCTACCT

CL2833.CONTIG3 AATCGAGAATGGACGAGCAACAGGGGGACGTCTCGCTCTGTACACGGCAGCGCTCTACCT

CL2883.CONTIG2_ALL AATCGAGAATGGACGAGCAACAGGGGGACGTCTCGCTCTGTACACGGCAGCGCTCTACCT

**** *******************************************************

CL2883.CONTIG1_ALL CATCCCACGACCCAGCATTGAGGCAGTCAAGGCTCTCACGCCACTCTTTGAAAGCCCTCG

CL2883.CONTIG4_ALL CATCCCACGACCCAGCATTGAGGCAGTCAAGGCTCTCACGCCACTCTTTGAAAGCCCTCG

CL2883.CONTIG5_ALL CATCCCACGACCCAGCATTGAGGCAGTCAAGGCTCTCACGCCACTCTTTGAAAGCCCTCG

XM_027379601 CATCCCACGACCCAGCATTGAGGCAGTCAAGGCTCTCACGCCACTCTTTGAAAGCCCTCG

AY321153.2 CATCCCACGACCCAGCATTGAGGCAGTCAAGGCTCTCACGCCACTCTTTGAAAGCCCTCG

CL2833.CONTIG3 CATCCCACGACCCAGCATTGAGGCAGTCAAGGCTCTCACGCCACTCTTTGAAAGCCCTCG

CL2883.CONTIG2_ALL CACCCCACGACCCAGCATTGAGGCAGTCAAGGCTCTCACGCCACTCTTTGAAAGCCCTCG

** *********************************************************

CL2883.CONTIG1_ALL CCCAGTGCCCTCGGTGCTGCTGGCAGCTGCTTCCATGATAAACCACTACTGCCTTCATAC

CL2883.CONTIG4_ALL CCCAGTGCCCTCGGTGCTGCTGGCAGCTGCTTCCATGATAAACCACTACTGCCTTCATAC

CL2883.CONTIG5_ALL CCCAGTGCCCTCGGTGCTGCTGGCAGCTGCTTCCATGATAAACCACTACTGCCTTCATAC

XM_027379601 CCCAGTGCCCTCGGTGCTGCTGGCAGCTGCTTCCATGATAAACCACTACTGCCTTCATAC

AY321153.2 CCCAGTGCCCTCGGTGCTGCTGGCAGCTGCTTCCATGATAAACCACTACTGCCTTCATAC

CL2833.CONTIG3 CCCAGTGCCCTCGGTGCTGCTGGCAGCTGCTTCCATGATAAACCACTACTGCCTTCATAC

CL2883.CONTIG2_ALL CCCAGTGCCCTCGGTGTTGCTGGCAGCTGCTTCCATGATAAACCACTACTGCCTTCATAC

**************** *******************************************

CL2883.CONTIG1_ALL TCCAGCTTGCCACCAGAAAGCTCCAGTGGCGAGAATTGCAGAGATTCTGGCCACCAGAGT

CL2883.CONTIG4_ALL TCCAGCTTGCCACCAGAAAGCTCCAGTGGCGAGAATTGCAGAGATTCTGGCCACCAGAGT

CL2883.CONTIG5_ALL TCCAGCTTGCCACCAGAAAGCTCCAGTGGCGAGAATTGCAGAGATTCTGGCCACCAGAGT

XM_027379601 TCCAGCTTGCCACCAGAAAGCTCCAGTGGCGAGAATTGCAGAGATTCTGGCCACCAGAGT

AY321153.2 TCCAGCTTGCCACCAGAAAGCTCCAGTGGCGAGAATTGCAGAGATTCTGGCCACCAGAGT

CL2833.CONTIG3 TCCAGCTTGCCACCAGAAAGCTCCAGTGGCGAGAATTGCAGAGATTCTGGCCACCAGAGT

CL2883.CONTIG2_ALL TCCAGCTTGCCACCAGAAAGCTCCAGTGGCGAGAATTGCAGAGATTCTGGCCACCAGAGT

************************************************************

CL2883.CONTIG1_ALL CCAGAGTCACTGCTCTCCTTCTGCTGGTGCTGAGGGCGAGGAAGTACCCCTTGCATTCTT

CL2883.CONTIG4_ALL CCAGAGTCACTGCTCTCCTTCTGCTGGTGCTGAGGGCGAGGAAGTACCCCTTGCATTCTT

CL2883.CONTIG5_ALL CCAGAGTCACTGCTCTCCTTCTGCTGGTGCTGAGGGCGAGGAAGTACCCCTTGCATTCTT

XM_027379601 CCAGAGTCACTGCTCTCCTTCTGCTGGTGCTGAGGGCGAGGAAGTACCCCTTGCATTCTT

AY321153.2 CCAGAGTCACTGCTCTCCTTCTGCTGGTGCTGAGGGCGAGGAAGTACCCCTTGCATTCTT

CL2833.CONTIG3 CCAGAGTCACTGCTCTCCTTCTGCTGGTGCTGAGGGCGAGGAAGTACCCCTTGCATTCTT

CL2883.CONTIG2_ALL CCAGAGTCACTGCTCTCCTTCTGCTGGTGCTGAGGGCGAGGAAGTACCCCTTGCATTCTT

************************************************************

CL2883.CONTIG1_ALL CAAGGCAATAGGGAATATGGGTGTAGCTACACCTGCCGTGACAAGGGCAGCCGTCCAATG

CL2883.CONTIG4_ALL CAAGGCAATAGGGAATATGGGTGTAGCTACACCTGCCGTGACAAGGGCAGCCGTCCAATG

CL2883.CONTIG5_ALL CAAGGCAATAGGGAATATGGGTGTAGCTACACCTGCCGTGACAAGGGCAGCCGTCCAATG

XM_027379601 CAAGGCAATAGGGAATATGGGTGTAGCTACACCTGCCGTGACAAGGGCAGCCGTCCAATG

AY321153.2 CAAGGCAATAGGGAATATGGGTGTAGCTACACCTGCCGTGACAAGGGCAGCCGTCCAATG

CL2833.CONTIG3 CAAGGCAATAGGGAATATGGGTGTAGCTACACCTGCCGTGACAAGGGCAGCCGTCCAATG

CL2883.CONTIG2_ALL CAAGGCAATAGGGAATATGGGTGTAGCTACACCTGCCGTGACAAGGGCAGCCGTCCAATG

************************************************************

CL2883.CONTIG1_ALL CATTGAAGAAGAAGGACTGGAAACCAGCATTCGGGTAGCTGCAGCACAAGCCTTCAGACA

CL2883.CONTIG4_ALL CATTGAAGAAGAAGGACTGGAAACCAGCATTCGGGTAGCTGCAGCACAAGCCTTCAGACA

CL2883.CONTIG5_ALL CATTGAAGAAGAAGGACTGGAAACCAGCATTCGGGTAGCTGCAGCACAAGCCTTCAGACA

XM_027379601 CATTGAAGAAGAAGGACTGGAAACCAGCATTCGGGTAGCTGCAGCACAAGCCTTCAGACA

AY321153.2 CATTGAAGAAGAAGGACTGGAAACCAGCATTCGGGTAGCTGCAGCACAAGCCTTCAGACA

CL2833.CONTIG3 CATTGAAGAAGAAGGACTGGAAACCAGCATTCGGGTAGCTGCAGCACAAGCCTTCAGACA

CL2883.CONTIG2_ALL CATTGAAGAAGAAGGACTGGAAACCAGCATTCGGGTAGCTGCAGCACAAGCCTTCAGACA

************************************************************

CL2883.CONTIG1_ALL AGCCAATTGCTTCCGTCCAGCAGTTGAAAAGCTAGTAGACATTGCCGTCCGACCAGCCTT

CL2883.CONTIG4_ALL AGCCAATTGCTTCCGTCCAGCAGTTGAAAAGCTAGTAGACATTGCCGTCCGACCAGCCTT

CL2883.CONTIG5_ALL AGCCAATTGCTTCCGTCCAGCAGTTGAAAAGCTAGTAGACATTGCCGTCCGACCAGCCTT

XM_027379601 AGCCAATTGCTTCCGTCCAGCAGTTGAAAAGCTAGTAGACATTGCCGTCCGACCAGCCTT

AY321153.2 AGCCAATTGCTTCCGTCCAGCAGTTGAAAAGCTAGTAGACATTGCCGTGCGACCAGCCTT

CL2833.CONTIG3 AGCCAATTGCTTCCGTCCAGCAGTTGAAAAGCTAGTAGACATTGCCGTCCGACCAGCCTT

CL2883.CONTIG2_ALL AGCCAATTGCTTCCGTCCAGCAGTTGAAAAGCTAGTAGACATTGCCGTCCGACCAGCCTT

************************************************ ***********

CL2883.CONTIG1_ALL TGACACCGAAGTCCGCATCGCTTCCTATCTGGCAGCTGTCCGATGTGCTGAACAGGAACA

CL2883.CONTIG4_ALL TGACACCGAAGTCCGCATCGCTTCCTATCTGGCAGCTGTCCGATGTGCTGAACAGGAACA

CL2883.CONTIG5_ALL TGACACCGAAGTCCGCATCGCTTCCTATCTGGCAGCTGTCCGATGTGCTGAACAGGAACA

XM_027379601 TGACACCGAAGTCCGCATCGCTTCCTATCTGGCAGCTGTCCGATGTGCTGAACAGGAACA

AY321153.2 TGACACCGAAGTCCGCATCGCTTCCTATCTGGCAGCTGTCCGATGTGCTGAACAGGAACA

CL2833.CONTIG3 TGACACCGAAGTCCGCATCGCTTCCTATCTGGCAGCTGTCCGATGTGCTGAACAGGAACA

CL2883.CONTIG2_ALL TGACACCGAAGTCCGCATCGCTTCCTATCTGGCAGCTGTCCGATGTGCTGAACAGGAACA

************************************************************

CL2883.CONTIG1_ALL CCTGGAGAAAATTATTGAGAAGATTTCAAAGGAAGAGAATACCCAAGTGCGTGGATTTGT

CL2883.CONTIG4_ALL CCTGGAGAAAATTATTGAGAAGATTTCAAAGGAAGAGAATACCCAAGTGCGTGGATTTGT

CL2883.CONTIG5_ALL CCTGGAGAAAATTATTGAGAAGATTTCAAAGGAAGAGAATACCCAAGTGCGTGGATTTGT

XM_027379601 CCTGGAGAAAATTATTGAGAAGATTTCAAAGGAAGAGAATACCCAAGTGCGTGGATTTGT

AY321153.2 CCTGGAGAAAATTATTGAGAAGATTTCAAAGGAAGAGAATACCCAAGTGCGTGGATTTGT

CL2833.CONTIG3 CCTGGAGAAAATTATTGAGAAGATTTCAAAGGAAGAGAATACCCAAGTGCGTGGATTTGT

CL2883.CONTIG2_ALL CCTGGAGAAAATTATTGAGAAGATTTCAAAGGAAGAGAATACTCAAGTGCGTGGATTTGT

****************************************** *****************

CL2883.CONTIG1_ALL TTTGGGTCACCTGATCAACATCCAAGAGGGTAGCTGCCCCAACAAAGAAAACCTCAGGTA

CL2883.CONTIG4_ALL TTTGGGTCACCTGATCAACATCCAAGAGGGTAGCTGCCCCAACAAAGAAAACCTCAGGTA

CL2883.CONTIG5_ALL TTTGGGTCACCTGATCAACATCCAAGAGGGTAGCTGCCCCAACAAAGAAAACCTCAGGTA

XM_027379601 TTTGGGTCACCTGATCAACATCCAAGAGGGTAGCTGCCCCAACAAAGAAAACCTCAGGTA

AY321153.2 TTTGGGTCACCTGATCAACATCCAAGAGGGTAGCTGCCCCAACAAAGAAAACCTCAGGTA

CL2833.CONTIG3 TTTGGGTCACCTGATCAACATCCAAGAGGGTAGCTGCCCCAACAAAGAAAACCTCAGGTA

CL2883.CONTIG2_ALL TTTGGGTCACCTGATCAACATCCAAGAGGGTAGCTGCCCCAACAAAGAAAACCTCAGGTA

************************************************************

CL2883.CONTIG1_ALL CCTCCTTGCCAACGTTGTCATCCCTACCGACTTCGAGAAGGACTTCAGGAAATTCTCTCG

CL2883.CONTIG4_ALL CCTCCTTGCCAACGTTGTCATCCCTACCGACTTCGAGAAGGACTTCAGGAAATTCTCTCG

CL2883.CONTIG5_ALL CCTCCTTGCCAACGTTGTCATCCCTACCGACTTCGAGAAGGACTTCAGGAAATTCTCTCG

XM_027379601 CCTCCTTGCCAACGTTGTCATCCCTACCGACTTCGAGAAGGACTTCAGGAAATTCTCTCG

AY321153.2 CCTCCTTGCCAACGTTGTCATCCCTACCGACTTCGAGAAGGACTTCAGGAAATTCTCCCG

CL2833.CONTIG3 CCTCCTTGCCAACGTTGTCATCCCTACCGACTTCGAGAAGGACTTCAGGAAATTCTCTCG

CL2883.CONTIG2_ALL CCTCCTTGCCAACGTTGTCATCCCTACCGACTTCGAGAAGGACTTCAGGAAATTCTCTCG

********************************************************* **

CL2883.CONTIG1_ALL ACATATAGATATGGCTTACTATGCCCCTGCCTTTGGCATGGGTGCCGGCCTCGAGTCGAA

CL2883.CONTIG4_ALL ACATATAGATATGGCTTACTATGCCCCTGCCTTTGGCATGGGTGCCGGCCTCGAGTCGAA

CL2883.CONTIG5_ALL ACATATAGATATGGCTTACTATGCCCCTGCCTTTGGCATGGGTGCCGGCCTCGAGTCGAA

XM_027379601 ACATATAGATATGGCTTACTATGCCCCTGCCTTTGGCATGGGTGCCGGCCTCGAGTCGAA

AY321153.2 ACATATAGATATGGCTTACTATGCCCCTGCCTTTGGCATGGGTGCCGGCCTCGAGTCGAA

CL2833.CONTIG3 ACATATAGATATGGCTTACTATGCCCCTGCCTTTGGCATGGGTGCCGGCCTCGAGTCGAA

CL2883.CONTIG2_ALL ACATATAGATATGGCTTACTATGCCCCTGCCTTTGGCATGGGTGCCGGCCTCGAGTCGAA

************************************************************

CL2883.CONTIG1_ALL CATCATCTATGCTCCCGGATCTTTCATTCCTCGTGCTGTTAACCTGAACATGAGAGCAAC

CL2883.CONTIG4_ALL CATCATCTATGCTCCCGGATCTTTCATTCCTCGTGCTGTTAACCTGAACATGAGAGCAAC

CL2883.CONTIG5_ALL CATCATCTATGCTCCCGGATCTTTCATTCCTCGTGCTGTTAACCTGAACATGAGAGCAAC

XM_027379601 CATCATCTATGCTCCCGGATCTTTCATTCCTCGTGCTGTTAACCTGAACATGAGAGCAAC

AY321153.2 CATCATCTATGCTCCCGGATCTTTCATTCCTCGTGCTGTTAACCTGAACATGAGAGCAAC

CL2833.CONTIG3 CATCATCTATGCTCCCGGATCTTTCATTCCTCGTGCTGTTAACCTGAACATGAGAGCAAC

CL2883.CONTIG2_ALL CATCATCTATGCTCCCGGATCTTTCATTCCTCGTGCTGTTAACCTGAACATGAGAGCAAC

************************************************************

CL2883.CONTIG1_ALL TGTGGATGAGACGCCCATGGACATAGCAGAGATTGGTGCGCGCTTTGAAGGAGTCGACTC

CL2883.CONTIG4_ALL TGTGGATGAGACGCCCATGGACATAGCAGAGATTGGTGCGCGCTTTGAAGGAGTCGACTC

CL2883.CONTIG5_ALL TGTGGATGAGACGCCCATGGACATAGCAGAGATTGGTGCGCGCTTTGAAGGAGTCGACTC

XM_027379601 TGTGGATGAGACGCCCATGGACATAGCAGAGATTGGTGCGCGCTTTGAAGGAGTCGATTC

AY321153.2 TGTGGATGAGACGCCCATGGACATAGCAGAGATTGGTGCGCGCTTTGAAGGAGTCGACTC

CL2833.CONTIG3 TGTGGATGAGACGCCCATGGACATAGCAGAGATTGGTGCGCGCTTTGAAGGAGTAGATTC

CL2883.CONTIG2_ALL TGTGGATGAGACGCCCATGGACATAGCAGAGATTGGTGCGCGCTTTGAAGGAGTCGACTC

******************************************************.** **

CL2883.CONTIG1_ALL CATCATTGAAGAGCTCTTGGGCCCACAGGGATACCTACGCAAAGCAACATTTGGAAAGAT

CL2883.CONTIG4_ALL CATCATTGAAGAGCTCTTGGGCCCACAGGGATACCTACGCAAAGCAACATTTGGAAAGAT

CL2883.CONTIG5_ALL CATCATTGAAGAGCTCTTGGGCCCACAGGGATACCTACGCAAAGCAACATTTGGAAAGAT

XM_027379601 CATCATTGAAGAGCTCTTGGGCCCACAGGGATACCTACGCAAAGCAACATTTGGAAAGAT

AY321153.2 CATCATTGAAGAGCTCTTGGGCCCACAGGGATACCTACGCAAAGCAACATTTGGAAAGAT

CL2833.CONTIG3 CATCATTGAAGAGCTCTTGGGCCCACAGGGATACCTACGCAAAGCAACATTTGGAAAGAT

CL2883.CONTIG2_ALL CATCATTGAAGAGCTCTTGGGCCCACAGGGATACCTACGCAAAGCAACATTTGGAAAGAT

************************************************************

CL2883.CONTIG1_ALL TATGGAGGACATTACGGGTTTTGCAGGAGAGAAAGGCCTCAAGATCATGGAGCACTTCAA

CL2883.CONTIG4_ALL TATGGAGGACATTACGGGTTTTGCAGGAGAGAAAGGCCTCAAGATCATGGAGCACTTCAA

CL2883.CONTIG5_ALL TATGGAGGACATTACGGGTTTTGCAGGAGAGAAAGGCCTCAAGATCATGGAGCACTTCAA

XM_027379601 TATGGAGGACATTACGGGTTTTGCAGGAGAGAAAGGCCTCAAGATCATGGAGCACTTCAA

AY321153.2 TATGGAGGACATTACGGGTTTTGCAGGAGAGAAAGGCCTCAAGATCATGGAGCACATCAA

CL2833.CONTIG3 TATGGAGGACATTACGGGTTTTGCAGGAGAGAAAGGCCTCAAGATCATGGAGCACTTCAA

CL2883.CONTIG2_ALL TATGGAGGACATTACGGGTTTTGCAGGAGAGAAAGGCCTCAAGATCATGGAGCACATCAA

*******************************************************:****

CL2883.CONTIG1_ALL GCACACAATGAGGACCAGGCGATCCATCGATGCTTCTGTCATCTCCGACTTCTTCGGCAA

CL2883.CONTIG4_ALL GCACACAATGAGGACCAGGCGATCCATCGATGCTTCTGTCATCTCCGACTTCTTCGGCAA

CL2883.CONTIG5_ALL GCACACAATGAGGACCAGGCGATCCATCGATGCTTCTGTCATCTCCGACTTCTTCGGCAA

XM_027379601 GCACACAATGAGGACCAGGCGATCCATCGATGCTTCTGTCATCTCCGACTTCTTCGGCAA

AY321153.2 GCACACAATGAGGACCAGGCGATCCATCGATGCTTCTGTCATCTCCGACTTCTTCGGCAA

CL2833.CONTIG3 GCACACAATGAGGACCAGGCGATCCATCGATGCTTCTGTCATCTCCGACTTCTTCGGCAA

CL2883.CONTIG2_ALL GCACACAATGAGGACCAGGCGATCCATCGATGCTTCTGTCATCTCCGACTTCTTCGGCAA

************************************************************

CL2883.CONTIG1_ALL GCTGTATGGTGAGAGCAGTTCGCACACCCACGCCGATATATTCGCCCGGTTCATGGGGCA

CL2883.CONTIG4_ALL GCTGTATGGTGAGAGCAGTTCGCACACCCACGCCGATATATTCGCCCGGTTCATGGGGCA

CL2883.CONTIG5_ALL GCTGTATGGTGAGAGCAGTTCGCACACCCACGCCGATATATTCGCCCGGTTCATGGGGCA

XM_027379601 GCTGTATGGTGAGAGCAGTTCGCACACCCACGCCGATATATTCGCCCGGTTCATGGGACA

AY321153.2 GCTGTATGGTGAGAGCAGTTCGCACACCCACGCCGATATATTCGCCCGGTTCATGGGACA

CL2833.CONTIG3 GCTGTATGGTGAGAGCAGTTCGCACACCCACGCCGATATATTCGCCCGGTTCATGGGGCA

CL2883.CONTIG2_ALL GCTGTATGGTGAGAGCAGTTCGCACACCCACGCCGATATATTCGCCCGGTTCATGGGACA

*********************************************************.**

CL2883.CONTIG1_ALL CGAGATTACTTTCGCAGATGTTGCCCAAAGCCTCAAGGGTGTCACAGCTGACACACTCAT

CL2883.CONTIG4_ALL CGAGATTACTTTCGCAGATGTTGCCCAAAGCCTCAAGGGTGTCACAGCTGACACACTCAT

CL2883.CONTIG5_ALL CGAGATTACTTTCGCAGATGTTGCCCAAAGCCTCAAGGGTGTCACAGCTGACACACTCAT

XM_027379601 CGAGATTACTTTCGCAGATGTTGCCCAAAGCCTCAAGGGCGTCACAGCTGACACACTCAT

AY321153.2 CGAGATTACTTTCGCAGATGTTGCCCAAAGCCTCAAGGGCGTCACAGCTGACACACTCAT

CL2833.CONTIG3 CGAGATTACTTTCGCAGATGTTGCCCAAAGCCTCAAGGGTGTCACAGCTGACACACTCAT

CL2883.CONTIG2_ALL CGAGATTACTTTCGCAGATGTTGCCCAAAGCCTCAAGGGCGTCACAGCTGACACACTCAT

*************************************** ********************

CL2883.CONTIG1_ALL TGAGACTTTCTTCTCTTTCTTCGAGAATTCCTTGGAACATATGAAGGATCTTAACCTGAA

CL2883.CONTIG4_ALL TGAGACTTTCTTCTCTTTCTTCGAGAATTCCTTGGAACATATGAAGGATCTTAACCTGAA

CL2883.CONTIG5_ALL TGAGACTTTCTTCTCTTTCTTCGAGAATTCCTTGGAACATATGAAGGATCTTAACCTGAA

XM_027379601 TGAGACTTTCTTCTCTTTCTTCGAGAATTCCTTGGAACATATGAAGGATCTTAACCTGAA

AY321153.2 TGAGACTTTCTTCTCTTTCTTCGAGAATTCCTTGGAACATATGAAGGATCTTAACCTGAA

CL2833.CONTIG3 TGAGACTTTCTTCTCTTTCTTCGAGAATTCCTTGGAACATATGAAGGATCTTAACCTGAA

CL2883.CONTIG2_ALL TGAGACCTTCTTCTCTTTCTTCGAGAATTCCTTGGAACATATGAAGGATCTTAACCTAAA

****** **************************************************.**

CL2883.CONTIG1_ALL CACAGCAAGAACTGCTCAGCTTTCCATGGATTACTCACTGCCCACCATTCAGGGCACACC

CL2883.CONTIG4_ALL CACAGCAAGAACTGCTCAGCTTTCCATGGATTACTCACTGCCCACCATTCAGGGCACACC

CL2883.CONTIG5_ALL CACAGCAAGAACTGCTCAGCTTTCCATGGATTACTCACTGCCCACCATTCAGGGCACACC

XM_027379601 CACAGCAAGAACTGCTCAGCTTTCCATGGATTACTCACTGCCCACCATTCAGGGCACACC

AY321153.2 CACAGCAAGAACTGCTCAGCTTTCCATGGATTACTCACTGCCCACCATTCAGGGCACACC

CL2833.CONTIG3 CACAGCAAGAACTGCTCAGCTTTCCATGGATTACTCACTGCCCACCATTCAGGGCACACC

CL2883.CONTIG2_ALL CACAGCAAGAACTGCTCAGCTTTCCATGGATTACTCACTACCCACCATTCAGGGCACACC

***************************************.********************

CL2883.CONTIG1_ALL ACTCAAGCTGAACTTAGCTGCAACTGCTGTTGCTGGCCTCAAGATGGAGGGCAACGTCAA

CL2883.CONTIG4_ALL ACTCAAGCTGAACTTAGCTGCAACTGCTGTTGCTGGCCTCAAGATGGAGGGCAACGTCAA

CL2883.CONTIG5_ALL ACTCAAGCTGAACTTAGCTGCAACTGCTGTTGCTGGCCTCAAGATGGAGGGCAACGTCAA

XM_027379601 ACTCAAGCTGAACTTAGCTGCAACTGCTGTTGCTGGCCTCAAGATGGAGGGCAACGTCAA

AY321153.2 ACTCAAGCTGAACTTAGCTGCAACTGCTGTTGCTGGCCTCAAGATGGAGGGCAACGTCAA

CL2833.CONTIG3 ACTCAAGCTGAACTTAGCTGCAACTGCTGTTGCTGGCCTCAAGATGGAGGGCAACGTCAA

CL2883.CONTIG2_ALL ACTCAAGCTGAACTTAGCTGCAACTGCTGTTGCTGGCCTCAAGATGGAGGGCAACGTCAA

************************************************************

CL2883.CONTIG1_ALL CATTGGCCAGATCCTCTCTGACCTGGGCAATTCCCACACCGGCATCAAGGTGTTCCCAGG

CL2883.CONTIG4_ALL CATTGGCCAGATCCTCTCTGACCTGGGCAATTCCCACACCGGCATCAAGGTGTTCCCAGG

CL2883.CONTIG5_ALL CATTGGCCAGATCCTCTCTGACCTGGGCAATTCCCACACCGGCATCAAGGTGTTCCCAGG

XM_027379601 CATTGGCCAGATCCTCTCTGACCTGGGCAATTCCCACACCGGCATCAAGGTGTTCCCAGG

AY321153.2 CATTGGCCAGATCCTCTCTGACCTGGGCAATTCCCACACCGGCATCAAGGTGTTCCCAGG

CL2833.CONTIG3 CATTGGCCAGATCCTCTCTGACCTGGGCAATTCCCACACCGGCATCAAGGTGTTCCCAGG

CL2883.CONTIG2_ALL CATTGGCCAGATCCTCTCTGACCTGGGCAATTCCCACACCGGCATCAAGGTGTTCCCAGG

************************************************************

CL2883.CONTIG1_ALL CCTTTCTGTACAAGCCACTGGTTTTGTTGGCTTTGAGTGCCGCTTTACCAAGGTGGGAAT

CL2883.CONTIG4_ALL CCTTTCTGTACAAGCCACTGGTTTTGTTGGCTTTGAGTGCCGCTTTACCAAGGTGGGAAT

CL2883.CONTIG5_ALL CCTTTCTGTACAAGCCACTGGTTTTGTTGGCTTTGAGTGCCGCTTTACCAAGGTGGGAAT

XM_027379601 CCTTTCTGTACAAGCCACTGGTTTTGTTGGCTTTGAGTGCCGCTTTACCAAGGTGGGAAT

AY321153.2 CCTTTCTGTACAAGCCACTGGTTTTGTTGGCTTTGAGTGCCGCTTTACCAAGGTGGGAAT

CL2833.CONTIG3 CCTTTCTGTACAAGCCACTGGTTTTGTTGGCTTTGAGTGCCGCTTTACCAAGGTGGGAAT

CL2883.CONTIG2_ALL CCTTTCTGTACAAGCCACTGGTTTTGTTGGCTTTGAGTGCCGCTTTACCAAGGTGGGAAT

************************************************************

CL2883.CONTIG1_ALL CGAGATGCAGAACACCATCTCTAGTGCCACTGGAGCCGCCATCAACATCAGAACAACTGA

CL2883.CONTIG4_ALL CGAGATGCAGAACACCATCTCTAGTGCCACTGGAGCCGCCATCAACATCAGAACAACTGA

CL2883.CONTIG5_ALL CGAGATGCAGAACACCATCTCTAGTGCCACTGGAGCCGCCATCAACATCAGAACAACTGA

XM_027379601 CGAGATGCAGAACACCATCTCTAGTGCCACTGGAGCCGCCATCAACATCAGAACAACTGA

AY321153.2 CGAGATGCAGAACACCATCTCTAGTGCCACTGGAGCCGCCATCAACATCAGAACAACTGA

CL2833.CONTIG3 CGAGATGCAGAACACCATCTCTAGTGCCACTGGAGCCGCCATCAACATCAGAACAACTGA

CL2883.CONTIG2_ALL CGAGATGCAGAACACCATCTCTAGTGCCACTGGAGCCGCCATCAACATCAGAACAACTGA

************************************************************

CL2883.CONTIG1_ALL AAACAAGAAGATCGAGCTGGAATTGGAGATCCCTGACAAGATGGAACTCCTCAACATCAA

CL2883.CONTIG4_ALL AAACAAGAAGATCGAGCTGGAATTGGAGATCCCTGACAAGATGGAACTCCTCAACATCAA

CL2883.CONTIG5_ALL AAACAAGAAGATCGAGCTGGAATTGGAGATCCCTGACAAGATGGAACTCCTCAACATCAA

XM_027379601 AAACAAGAAGATCGAGCTGGAATTGGAGATCCCTGACAAGATGGAACTCCTCAACATCAA

AY321153.2 AAACAAGAAGATCGAGCTGGAATTGGAGATCCCTGACAAGATGGAACTCCTCAACATCAA

CL2833.CONTIG3 AAACAAGAAGATCGAGCTGGAATTGGAGATCCCTGACAAGATGGAACTCCTCAACATCAA

CL2883.CONTIG2_ALL AAACAAGAAGATCGAGCTGGAATTGGAGATCCCTGACAAGATGGAACTCCTCAACATCAA

************************************************************

CL2883.CONTIG1_ALL GGCCGAGACTTACCTTGTCAAAGCTAGGGGAAAGAAGATGACTAAGATTTCTCCTTCCTC

CL2883.CONTIG4_ALL GGCCGAGACTTACCTTGTCAAAGCTAGGGGAAAGAAGATGACTAAGATTTCTCCTTCCTC

CL2883.CONTIG5_ALL GGCCGAGACTTACCTTGTCAAAGCTAGGGGAAAGAAGATGACTAAGATTTCTCCTTCCTC

XM_027379601 GGCCGAGACTTACCTTGTCAAAGCTAGGGGAAAGAAGATGACTAAGATTTCTCCTTCCTC

AY321153.2 GGCCGAGACTTACCTTGTCAAAGCTAGGGGAAAGAAGATGACTAAGATTTCTCCTTCCTC

CL2833.CONTIG3 GGCCGAGACTTACCTTGTCAAAGCTAGGGGAAAGAAGATGACTAAGATTTCTCCTTCCTC

CL2883.CONTIG2_ALL GGCCGAGACTTACCTTGTCAAAGCTAGGGGAAAGAAGATGACTAAGATTTCTCCTTCCTC

************************************************************

CL2883.CONTIG1_ALL CATGAGAGATGTCAGGATTGAGCGCAGGTCCTGCATTGCTGCTTTGGAACCAGTATTTGG

CL2883.CONTIG4_ALL CATGAGAGATGTCAGGATTGAGCGCAGGTCCTGCATTGCTGCTTTGGAACCAGTATTTGG

CL2883.CONTIG5_ALL CATGAGAGATGTCAGGATTGAGCGCAGGTCCTGCATTGCTGCTTTGGAACCAGTATTTGG

XM_027379601 CATGAGAGATGTCAGGATTGAGCGCAAGTCCTGCATTGCTGCTTTGGAACCAGTATTTGG

AY321153.2 CATGAGAGATGTCAGGATTGAGCGCAAGTCCTGCATTGCTGCTTTGGAACCAGTATTTGG

CL2833.CONTIG3 CATGAGAGATGTCAGGATTGAGCGCAGGTCCTGCATTGCTGCTTTGGAACCAGTATTTGG

CL2883.CONTIG2_ALL CATGAGAGATGTCAGGATTGAGCGCAAGTCCTGCATTGCTGCTTTGGAACCAGTATTTGG

**************************.*********************************

CL2883.CONTIG1_ALL CCTCAAGGTGTGCTATGACATGAACTTCCCTGATGTGTTCCGTGCTAATGCCCTGCCACT

CL2883.CONTIG4_ALL CCTCAAGGTGTGCTATGACATGAACTTCCCTGATGTGTTCCGTGCTAATGCCCTGCCACT

CL2883.CONTIG5_ALL CCTCAAGGTGTGCTATGACATGAACTTCCCTGATGTGTTCCGTGCTAATGCCCTGCCACT

XM_027379601 CCTCAAGGTGTGCTATGACATGAACTTCCCTGATGTGTTCCGTGCTAATGCCCTGCCACT

AY321153.2 CCTCAAGGTGTGCTATGACATGAACTTCCCTGATGTGTTCCGTGCTAATGCCCTGCCACT

CL2833.CONTIG3 CCTCAAGGTGTGCTATGACATGAACTTCCCTGATGTGTTCCGTGCTAATGCCCTGCCACT

CL2883.CONTIG2_ALL CCTCAAGGTGTGCTATGACATGAACTTCCCTGATGTGTTCCGTGCTAATGCCCTGCCACT

************************************************************

CL2883.CONTIG1_ALL TGGTGAACCAGCCATCGCCAAGCTGTACGTTGAGAAGGCAGATCCTTCCATGAGAGGTTA

CL2883.CONTIG4_ALL TGGTGAACCAGCCATCGCCAAGCTGTACGTTGAGAAGGCAGATCCTTCCATGAGAGGTTA

CL2883.CONTIG5_ALL TGGTGAACCAGCCATCGCCAAGCTGTACGTTGAGAAGGCAGATCCTTCCATGAGAGGTTA

XM_027379601 TGGTGAACCAGCCATCGCCAAGCTGTACGTTGAGAAGGCAGATCCTTCCATGAGAGGTTA

AY321153.2 TGGTGAACCAGCCATCGCCAAGCTGTACGTTGAGAAGGCAGATCCTTCCATGAGAGGTTA

CL2833.CONTIG3 TGGTGAACCAGCCATCGCCAAGCTGTACGTTGAGAAGGCAGATCCTTCCATGAGAGGTTA

CL2883.CONTIG2_ALL TGGTGAACCAGCCATCGCCAAGCTGTACGTTGAGAAGGCAGATCCTTCCATGAGAGGTTA

************************************************************

CL2883.CONTIG1_ALL CTTAGTGACTGCTGCCATTAAGAACAAGAGAGGCAACAAGCTCATTAAGATGAATGTAGA

CL2883.CONTIG4_ALL CTTAGTGACTGCTGCCATCAAGAACAAGAGAGGTAACAAGCTCATTAAGATGAATGTAGA

CL2883.CONTIG5_ALL CTTAGTGACTGCTGCCATCAAGAACAAGAGAGGTAACAAGCTCATTAAGATGAATGTAGA

XM_027379601 CTTAGTGACTGCTGCCATCAAGAACAAGAGAGGTAACAAGCTCATTAAGATGAATGTAGA

AY321153.2 CTTAGTGACTGCTGCCATCAAGAACAAGAGAGGTAACAAGCTCATTAAGATGAATGTAGA

CL2833.CONTIG3 CTTAGTGACTGCTGCCATCAAGAACAAGAGAGGTAACAAGCTCATTAAGATGAATGTAGA

CL2883.CONTIG2_ALL CTTAGTGACTGCTGCCATCAAGAACAAGAGAGGTAACAAGCTCATTAAGATGAATGTAGA

****************** ************** **************************

CL2883.CONTIG1_ALL AGCAGCTGGTGCCTCAACACCAAGAAGAGCAGAAATGACCCTGTCCTACACCAAGGAAGA

CL2883.CONTIG4_ALL AGCAGCTGGTGCCTCAACACCAAGAAGAGCAGAAATGACCCTGTCCTACACCAAGGAAGA

CL2883.CONTIG5_ALL AGCAGCTGGTGCCTCAACACCAAGAAGAGCAGAAATGACCCTGTCCTACACCAAGGAAGA

XM_027379601 AGCAGCTGGTGCCTCAACACCAAGAAGAGCAGAAATGACCCTGTCCTACACCAAGGAAGA

AY321153.2 AGCAGCTGGTGCCTCAACACCAAGAAGAGCAGAAATGACCCTGTCCTACACCAAGGAAGA

CL2833.CONTIG3 AGCAGCTGGTGCCTCAACACCAAGAAGAGCAGAAATGACCCTGTCCTACACCAAGGAAGA

CL2883.CONTIG2_ALL AGCAGCTGGTGCCTCAACACCAAGAAGAGCAGAAATGACCCTGTCCTACACCAAGGAAGA

************************************************************

CL2883.CONTIG1_ALL AGGAAGCCACATTGTTTCTGCCAAGCTTGATTCCTCCAGCATTGCTGCAGGAGTGTGGAC

CL2883.CONTIG4_ALL AGGAAGCCACATTGTTTCTGCCAAGCTTGATTCCTCCAGCATTGCTGCAGGAGTGTGGAC

CL2883.CONTIG5_ALL AGGAAGCCACATTGTTTCTGCCAAGCTTGATTCCTCCAGCATTGCTGCAGGAGTGTGGAC

XM_027379601 AGGAAGCCACATTGTTTCTGCCAAGCTTGATTCCTCCAGCATTGCTGCAGGAGTGTGGAC

AY321153.2 AGGAAGCCACATTGTTTCTGCCAAGCTTGATTCCTCCAGCATTGCTGCAGGAGTGTGGAC

CL2833.CONTIG3 AGGAAGCCACATTGTTTCTGCCAAGCTTGATTCCTCCAGCATTGCTGCAGGAGTGTGGAC

CL2883.CONTIG2_ALL AGGAAGCCACATTGTTTCTGCCAAGCTTGATTCCTCCAGCATTGCTGCAGGAGTGTGGAC

************************************************************

CL2883.CONTIG1_ALL TACTCTCACCAACGAGCAAGGACACAAGGCAGTAGAGACTTATGTCAACTTCAAATATGG

CL2883.CONTIG4_ALL TACTCTCACCAACGAGCAAGGACACAAGGCAGTAGAGACTTATGTCAACTTCAAATATGG

CL2883.CONTIG5_ALL TACTCTCACCAACGAGCAAGGACACAAGGCAGTAGAGACTTATGTCAACTTCAAATATGG

XM_027379601 TACTCTCACCAACGAGCAAGGACACAAGGCAGTAGAGACTTATGTCAACTTCAAATATGG

AY321153.2 CACTCTCATCAACGAGCAAGGACACAAGGCAGTAGAGACTTATGTCAACTTCAAATATGG

CL2833.CONTIG3 TACTCTCACCAACGAGCAAGGACACAAGGCAGTAGAGACTTATGTCAACTTCAAATATGG

CL2883.CONTIG2_ALL TACTCTCACCAACGAGCAAGGACACAAGGCAGTAGAGACTTATGTCAACTTCAAATATGG

******* ***************************************************

CL2883.CONTIG1_ALL TCAGACTGCTATTTCTCGAGGCATCAAGCTGGAAGCGATTGCAAGGGAAGGAAGCGTGGG

CL2883.CONTIG4_ALL TCAGACTGCTATTTCTCGAGGCATCAAGCTGGAAGCGATTGCAAGGGAAGGAAGCGTGGG

CL2883.CONTIG5_ALL TCAGACTGCTATTTCTCGAGGCATCAAGCTGGAAGCGATTGCAAGGGAAGGAAGCGTGGG

XM_027379601 TCAGACTGCTATTTCTCGAGGCATCAAGCTGGAAGCGATTGCAAGGGAAGGAAGTGTGGG

AY321153.2 TCAGACTGCTATTTCTCGAGGCATCAAGCTGGAAGCGATTGCAAGGGAAGGAAGTGTGGG

CL2833.CONTIG3 TCAGACTGCTATTTCTCGAGGCATCAAGCTGGAAGCGATTGCAAGGGAAGGAAGCGTGGG

CL2883.CONTIG2_ALL TCAGACTGCTATTTCTCGAAGCATCAAGCTGGAAGCGATTGCAAGGGAAGGAAGTGTGGG

*******************.********************************** *****

CL2883.CONTIG1_ALL AGAGGAATTCCAAGTGAACGTTTTCAGCAGCGGCACCAGGAGCTTCCCCTCTAAATCTCA

CL2883.CONTIG4_ALL AGAGGAATTCCAAGTGAACGTTTTCAGCAGCGGCACCAGGAGCTTCCCCCTCGACTCTCA

CL2883.CONTIG5_ALL AGAGGAATTCCAAGTGAACGTTTTCAGCAGCGGCACCAGGAGCTTCCCCCTCGACTCTCA

XM_027379601 AGAGGAATTCCAAGTGAACGTTTTCAGCAGCGGCACCAGGAGCTTCCCCCTCGACTCTCA

AY321153.2 AGAGGAATTCCAAGTGAACGTTTTCAGCAGCGGCACCAGGAGCTTCCCCCTCGACTCTCA

CL2833.CONTIG3 AGAGGAATTCCAAGTGAACGTTTTCAGCAGCGGCACCAGGAGCTTCCCCTCTAAATCTCA

CL2883.CONTIG2_ALL AGAGGAATTCCAAGTGAACGTTTTCAGCAGCGGCACCAGGAGCTTCCCCTCTAAATCTCA

************************************************* .*.*****

CL2883.CONTIG1_ALL AATTGTAGAGGCTAAATTCATCAAGAAAACTAGTGGACCTGAATTCAATGTGGATGTGAT

CL2883.CONTIG4_ALL CATTGTGGAGGCTAAATTCATCAAGAAAACTAGTGGACCTGAATTCAATGTGGATGTGAT

CL2883.CONTIG5_ALL CATTGTGGAGGCTAAATTCATCAAGAAAACTAGTGGACCTGAATTCAATGTGGATGTGAT

XM_027379601 CATTGTGGAGGCTAAATTCATCAAGAAAACTAGTGGACCTGAATTCAATGTGGATGTGAT

AY321153.2 CATTGTGGAGGCTAAATTCATCAAGAAAACTAGTGGACCTGAATTCAATGTGGATGTGAT

CL2833.CONTIG3 AATTGTAGAGGCTAAATTCATCAAGAAAACTAGCGGACCCGTAGTTAATGTGGATGTGAT

CL2883.CONTIG2_ALL AATTGTAGAGGCTAAATTCATCAAGAAAACTAGCGGACCCGTAGTTAATGTGGATGTGAT

.*****.************************** ***** *:* * **************

CL2883.CONTIG1_ALL CTGCATGACCAAAAATGCTTTAGCTGATTATTTCGACTTAAACATTGAAGTTGGAGCTGA

CL2883.CONTIG4_ALL CTGCATGACCAAAAATGCTTTAGCTGATTATTTCGACTTAAACATTGAAGTTGGAGCTGA

CL2883.CONTIG5_ALL CTGCATGACCAAAAATGCTTTAGCTGATTATTTCGACTTAAACATTGAAGTTGGAGCTGA

XM_027379601 CTGCATGACCAAAAATGCTTTAGCTGATTATTTCGACTTAAACATTGAAGTTGGAGCTGA

AY321153.2 CTGCATGACCAAAAATGCTTTAGCTGATTATTTCGACTTAAACATTGAAGTTGGAGCTGA

CL2833.CONTIG3 TTGCAGGACCAGAAATGGTCTGGCTGAATACTTCAACCTCAATATGGAAGTGGGAGCTGA

CL2883.CONTIG2_ALL TTGCAGGACCAGAAATGGTCTGGCTGAATACTTCAACCTCAATATGGAAGTGGGAGCTGA

**** *****.***** * *.*****:** ***.** *.** ** ***** ********

CL2883.CONTIG1_ALL TTTCATGAGATTTTCTCCTAAAGCTCTGTATTCAACAAGATACATTCCCAAGACCCGCAT

CL2883.CONTIG4_ALL TTTCATGAGATTTTCTCCTAAAGCTCTGTATTCAACAAGATACATTCCCAAGACCCGCAT

CL2883.CONTIG5_ALL TTTCATGAGATTTTCTCCTAAAGCTCTGTATTCAACAAGATACATTCCCAAGACCCGCAT

XM_027379601 TTTCATGAGATTTTCTCCTAAGGCTCTGTATTCAACAAGATACATTCCCAAGACCCGCAT

AY321153.2 TTTCATGAGATTTTCTCCTAAAGCTCTGTATTCAACAAGATACATTCCCAAGACCCGCAT

CL2833.CONTIG3 CCTCATGGAGTTCTCCTCAGAGGACACATACAGGACCAGGTACATTCCCAAGTTCCGCAT

CL2883.CONTIG2_ALL CCTCATGGAGTTCTCCTCAGAGGACACATACAGGACCAGGTACATTCCCAAGTTCCGCAT

*****...** ** *:.*.*. . .** : .**.**.************: ******

CL2883.CONTIG1_ALL TTTCTTACCTGTAAACCTGCGAAAGCTAGAAATCAATGCTGCCACTGCAGCCTGGAAAGT

CL2883.CONTIG4_ALL TTTCTTACCTGTAAACCTGCGAAAGCTAGAAATCAATGCTGCCACTGCAGCCTGGAAAGT

CL2883.CONTIG5_ALL TTTCTTACCTGTAAACCTGCGAAAGCTAGAAATCAATGCTGCCACTGCAGCCTGGAAAGT

XM_027379601 TTTCTTACCTGTAAACCTGCGAAAGCTAGAAATCAATGCTGCCACTGCAGCCTGGAAAGT

AY321153.2 TTTCTTACCTGTAAACCTGCGAAAGCTAGAAATCAATGCTGCCACTGCAGCCTGGAAAGT

CL2833.CONTIG3 TCTTTTCCCTGTAACTTTGCGGAAGGTGGAAGTGCATGCCGAAACTGGAGCTTGGAGACT

CL2883.CONTIG2_ALL TCTTTTCCCTGTAACTTTGCGGAAGGTGGAAGTGCATGCCGAAACTGGAGCTTGGAGACT

* * **.*******. ****.*** *.***.* .**** *..**** *** ****.* *

CL2883.CONTIG1_ALL GACGTCGTACATTCGTGAAGGAAGTCAATCTGGCGAAAGCCGTGAGTTCAGTTCTGCTTT

CL2883.CONTIG4_ALL GACGTCGTACATTCGTGAAGGAAGTCAATCTGGCGAAAGCCGTGAGTTCAGTTCTGCTTT

CL2883.CONTIG5_ALL GACGTCGTACATTCGTGAAGGAAGTCAATCTGGCGAAAGCCGTGAGTTCAGTTCTGCTTT

XM_027379601 GACGTCGTACATTCGTGAAGGAAGTCAATCTGGCGAAAGCCGTGAGTTCAGTTCTGCTTT

AY321153.2 GACGTCGTACATTCGTGAAGGAAGTCAATCTGGCGAAAGCCGTGAGTTCAGTTCTGCTTT

CL2833.CONTIG3 GGCATCATACATCCGCGAAGGCAGCCAGTCTGGACAAATCAGCGAGCACATTTCTGCACT

CL2883.CONTIG2_ALL GGCATCATACATCCGCGAAGGCAGCCAGTCTGGACAAATCAGCGAGCACATTTCTGCACT

*.*.**.***** ** *****.** **.*****. *** *.* *** :** ******: *

CL2883.CONTIG1_ALL CAAGCTTGCCAAGGGAAGGACGGATGTCATCTTTGTACAGGCTACTCATACGATTGAAGG

CL2883.CONTIG4_ALL CAAGCTTGCCAAGGGAAGGACGGATGTCATCTTTGTACAGGCTACTCATACGATTGAAGG

CL2883.CONTIG5_ALL CAAGCTTGCCAAGGGAAGGACGGATGTCATCTTTGTACAGGCTACTCATACGATTGAAGG

XM_027379601 CAAGCTTGCCAAGGGAAGGACGGATGTCATCTTTGTACAGGCTACTCATACGATTGAAGG

AY321153.2 CAAGCTTGCCAAGGGAAGGACGGATGTCATCTTTGTACAGGCTACTCATACGATTGAAGG

CL2833.CONTIG3 CAGGCTAGCAAAGGGAAGCAAAGATATCATCTCTGTTGAAGCCACTCACACGATTGAGGG

CL2883.CONTIG2_ALL CAGGCTAGCAAAGGGAAGCAAAGATATCATCTCTGTTGAAGCCACTCACACGATTGAGGG

**.***:**.******** *..***.****** ***: *.** ***** ********.**

CL2883.CONTIG1_ALL CAGATTCCCACAAAACGTCATCATCAAAAATGTAGCAACAGCCAAAGTTGGCAGATCATC

CL2883.CONTIG4_ALL CAGATTCCCACAAAACGTCATCATCAAAAATGTAGCAACAGCCAAAGTTGGCAGATCATC

CL2883.CONTIG5_ALL CAGATTCCCACAAAACGTCATCATCAAAAATGTAGCAACAGCCAAAGTTGGCAGATCATC

XM_027379601 CAGATTCCCACAAAACGTCATCATCAAAAATGTAGCAACAGCCAAAGTTGGCAGATCATC

AY321153.2 CAGATTCCCACAAAACGTCATCATCAAAAATGTAGCAACAGCCAAAGTTGGCAGATCATC

CL2833.CONTIG3 AGCTTTCCCGGAAAACATCATCATCAAAAATGAAGCAACAGTTGATATTGGCAGATCATC

CL2883.CONTIG2_ALL AGCTTTCCCGGAAAACATCATCATCAAAAATGAAGCAACAGTTGATATTGGCAGATCATC

.. :*****. *****.***************:******** .*:.*************

CL2883.CONTIG1_ALL ATACAGAGCAATGTATGATGTCTTCTATCACCCTGAAAAAGTGGGAGCTTCTATTGAGGT

CL2883.CONTIG4_ALL ATACAGAGCAATGTATGATGTCTTCTATCACCCTGAAAAAGTGGGAGCTTCTATTGAGGT

CL2883.CONTIG5_ALL ATACAGAGCAATGTATGATGTCTTCTATCACCCTGAAAAAGTGGGAGCTTCTATTGAGGT

XM_027379601 ATACAGAGCAATGTATGATGTCTTCTATCACCCTGAAAAAGTGGGAGCTTCTATTGAGGT

AY321153.2 ATACAGAGCAATGTATGATGTCTTCTATCACCCTGAAAAAGTGGGAGCTTCTATTGAGGT

CL2833.CONTIG3 ATACAAAGCCATCTATGATGTAATCTACCAGGCTGAAAAGGTCGGGGCTTCTATGGAAGT

CL2883.CONTIG2_ALL ATACAAAGCCATCTATGATGTAATCTACCAGGCTGAAAAGGTCGGGGCTTCTATGGAAGT

*****.***.** ********.:**** ** *******.** **.******** **.**

CL2883.CONTIG1_ALL TTTGCAGGCAGCAGGTAATGAGAAGGTTGCCCAGATAGAAGCAATTTACGAAATTTCCGG

CL2883.CONTIG4_ALL TTTGCAGGCAGCAGGTAATGAGAAGGTTGCCCAGATAGAAGCAATTTACGAAATTTCCGG

CL2883.CONTIG5_ALL TTTGCAGGCAGCAGGTAATGAGAAGGTTGCCCAGATAGAAGCAATTTACGAAATTTCCGG

XM_027379601 TTTGCAGGCAGCAGGTAATGAGAAGGTTGCCCAGATAGAAGCAATTTACGAAATTTCCGG

AY321153.2 TTTGCAGGCAGCAGGTAATGAGAAGGTTGCCCAGATAGAAGCAATTTACGAAATTTCCGG

CL2833.CONTIG3 CGTCCGCACGGGAGACAATGAGAAAATTGTTAAACTGGACGCAATCTATCATCATTCAGG

CL2883.CONTIG2_ALL CGTCCGCACGGGAGACAATGAGAAAATTGTTAAACTGGACGCAATCTATCATCATTCAGG

* *. .*.* **. ********..*** .*..*.**.***** ** *:.:***.**

CL2883.CONTIG1_ALL AGAGAAGCACTGCGCTAAATTCTTGGCGGCCATTCCTGGCTACATTCAACCAGTTAAAGT

CL2883.CONTIG4_ALL AGAGAAGCACTGCGCTAAATTCTTGGCGGCCATTCCTGGCTACATTCAACCAGTTAAAGT

CL2883.CONTIG5_ALL AGAGAAGCACTGCGCTAAATTCTTGGCGGCCATTCCTGGCTACATTCAACCAGTTAAAGT

XM_027379601 AGAGAAGCACTGCGCTAAATTCTTGGCGGCCATTCCTGGCTACATTCAACCAGTTAAAGT

AY321153.2 AGAGAAGCACTGCGCTAAATTCTTGGCGGCCATTCCTGGCTACATTCAACCAGTTAAAGT

CL2833.CONTIG3 AGCAGAATACAGCACTAATTTCTTGCTGAAGGTTCCCACTTACATGAAACCTCTCAAGAT

CL2883.CONTIG2_ALL AGCAGAATACAGCACTAATTTCTTGCTGAAGGTTCCCACTTACATGAAACCTCTCAAGAT

**...*. **:**.****:****** *.. .**** . ***** .****: * **..*

CL2883.CONTIG1_ALL TGAAGCCGAGATTGAACAAGAAGCAGAAGGTCGCTACGCACTGGAGGCCGCCATCAAATA

CL2883.CONTIG4_ALL TGAAGCCGAGATTGAACAAGAAGCAGAAGGTCGCTACGCACTGGAGGCCGCCATCAAATA

CL2883.CONTIG5_ALL TGAAGCCGAGATTGAACAAGAAGCAGAAGGTCGCTACGCACTGGAGGCCGCCATCAAATA

XM_027379601 TGAAGCCGAGATTGAACAAGAAGCAGAAGGTCGCTACGCACTGGAGGCCGCCATCAAATA

AY321153.2 TGAAGCCGAGATTGAACAAGAAGCAGAAGGTCGCTACGCACTGGAGTCCGCCATCAAATA

CL2833.CONTIG3 TCAGGCCAAGATTGGAGAAGAAGCAGAGGGTCGCTACATGCTGGAAACAGCCATCAAATA

CL2883.CONTIG2_ALL TCAGGCCAAGATTGGAGAAGAAGCAGAGGGTCGCTACATGCTGGAAACAGCCATCAAATA

* *.***.******.* **********.*********. .*****. *.***********

CL2883.CONTIG1_ALL TGGACCACGTACAGTACTTGGAGTGAGTGGACCAGTCCTAGCTCGTTTCACCTCCAAAGC

CL2883.CONTIG4_ALL TGGACCACGTACAGTACTTGGAGTGAGTGGACCAGTCCTAGCTCGTTTCACCTCCAAAGC

CL2883.CONTIG5_ALL TGGACCACGTACAGTACTTGGAGTGAGTGGACCAGTCCTAGCTCGTTTCACCTCCAAAGC

XM_027379601 TGGACCACGTACAGTACTTGGAGTGAGTGGACCAGTCCTAGCTCGTTTCACCTCCAAAG-

AY321153.2 TGGACCACGTACAGTACTTGGAGTGAGTGGACCAGTCCTAGCTCGTTTCACCTCCAAAGC

CL2833.CONTIG3 TGGTCAGCGTATGATACTTGAAGCTAATGGGCCAATCATGGCACGTCTTTCCTCCAAGAT

CL2883.CONTIG2_ALL TGGTCAGCGTATGATACTTGAAGCTAATGGGCCAATCATGGCACGTCTTTCCTCCAAGAT

***:*..**** ..******.** *.***.***.**.*.**:*** * :*******..

CL2883.CONTIG1_ALL CAACAAGCTGCAAGCCAACATCAAGCTCAGGGCAATGGCAAGTGAGCCCTACATCATTGG

CL2883.CONTIG4_ALL CAACAAGCTGCAAGCCAACATCAAGCTCAGGGCAATGGCAAGTGAGCCCTACATCATTGG

CL2883.CONTIG5_ALL CAACAAGCTGCAAGCCAACATCAAGCTCAGGGCAATGGCAAGTGAGCCCTACATCATTGG

XM_027379601 --CAAAGCTGCAAGCCAACATCAAGCTCAGGGCAATGGCAAGTGAGCCCTACATCATTGG

AY321153.2 CAACAAGCTGCAAGCCAACATCAAGCTCAGGGCAATGGCAAGTGAGCCCTACATCATTGG

CL2833.CONTIG3 TGCAAAGATGCAGGCCAACATCAAACTCAGTGCATTGGCAAGTGAACCCTACATCATTGG

CL2883.CONTIG2_ALL TGCAAAGATGCAGGCCAACATCAAACTCAGTGCATTGGCAAGTGAACCCTACATCATTGG

..***.****.***********.***** ***:**********.**************

CL2883.CONTIG1_ALL TGCCAATGTTGTGTTTGGCAACAAGAAACAGGTGATAGCCATGGAAATCAAGGAGCGAGA

CL2883.CONTIG4_ALL TGCCAATGTTGTGTTTGGCAACAAGAAACAGATGATCGCCATGGAAATCAAGGAGCGATC

CL2883.CONTIG5_ALL TGCCAATGTTGTGTTTGGCAACAAGAAACAGATGATCGCCATGGAAATCAAGGAGCGATC

XM_027379601 TGCCAATGTTGTGTTTGGCAACAAGAAACAGATGATCGCCATGGAAATCAAGGAGCGATC

AY321153.2 TGCCAATGTTGTGTTTGGCAACAAGAAACAGATGATCGCCATGGAAATCAAGGAGCGATC

CL2833.CONTIG3 TGCCAATGTTGTGTTTGGCAACAAGAAACAGGTGATAGCCATGGAAATCAAGGAGCGAGA

CL2883.CONTIG2_ALL TGCCAATGTTGTGTTTGGCAACAAGAAACAGGTGATAGCCATGGAAATCAAGGAGCGAGA

*******************************.****.********************* .

CL2883.CONTIG1_ALL AGAGCCACTCTTTGGTGTAGAATGGAAGATGGTTCAAGAGAGTTCTGAGAAGACCACTGT

CL2883.CONTIG4_ALL AGAACCTCTCATTGGTCTTGAATGGAGAATGGTCCGAGAAAGTTCCGAGAAGACCACTAT

CL2883.CONTIG5_ALL AGAACCTCTCATTGGTCTTGAATGGAGAATGGTCCGAGAAAGTTCCGAGAAGACCACTAT

XM_027379601 AGAACCTCTCATTGGTCTTGAATGGAGAATGGTCCGAGAAAGTTCCGAGAAGACCACTAT

AY321153.2 AGAACCTCTCATTGGTCTTGAATGGAGAATGGTCCGAGAAAGTTCCGAGAAGACCACTAT

CL2833.CONTIG3 AGAGCCACTCTTTGGTGTAGAATGGAAGATGGTTCAAGAGAGTTCTGAGAAGACCACTGT

CL2883.CONTIG2_ALL AGAGCCACTCTTTGGTGTAGAATGGAAGATGGTTCAAGAGAGTTCTGAGAAGACCACTGT

***.**:***:***** *:*******..***** *.***.***** ************.*

CL2883.CONTIG1_ALL TGGCATAGCATTCGTTCTCCCTGCCCTTATTGAGAACAAGGTCGATGCCATTATTACTGA

CL2883.CONTIG4_ALL TGGTGTAGTGTTTGTCCTCCCTGCCCTTATTGAGCAGAAAGTCGATGCTGAAATTACTGA

CL2883.CONTIG5_ALL TGGTGTAGTGTTTGTCCTCCCTGCCCTTATTGAGCAGAAAGTCGATGCTGAAATTACTGA

XM_027379601 TGGTGTAGTGTTTGTCCTCCCTGCCCTTATTGAGCAGAAAGTCGATGCTGAAATTACTGA

AY321153.2 TGGTGTAGTGTTTGTCCTCCCTGCCCTTATTGAGCAGAAAGTCGATGCTGAAATTACTGA

CL2833.CONTIG3 TGGCATAGCATTCGTTCTCCCTGCCCTTATTGAGAACAAGGTCGATGCCATTATTACTGA

CL2883.CONTIG2_ALL TGGCATAGCATTCGTTCTCCCTGCCCTTATTGAGAACAAGGTCGATGCCATTATTACTGA

*** .*** .** ** ******************.* **.******** .::********

CL2883.CONTIG1_ALL GGAACTTATCCATGTTAATTTTAACAACTTGGTTCTGCCTAATACTTCATCCCGCCGTCG

CL2883.CONTIG4_ALL TGAACTTGTCCATGTTAGTTTCAACAACCTGGTTCTACCCAAGACTTCATCCCGCCGTCG

CL2883.CONTIG5_ALL TGAACTTGTCCATGTTAGTTTCAACAACCTGGTTCTACCCAAGACTTCATCCCGCCGTCG

XM_027379601 TGAACTTGTCCATGTTAGTTTCAACAACCTGGTTCTACCCAAGACTTCATCCCGCCGTCG

AY321153.2 TGAACTTGTCCATGTTAGTTTCAACAACCTGGTTCTACCCAAGACTTCATCCCGCCGTCG

CL2833.CONTIG3 GGAACTTATCCATGTTAATTTTAACAACTTGGTTCTGCCTAATACTTCATCCCGCCGTCG

CL2883.CONTIG2_ALL GGACCTTATCCATGTTAATTTTAACAACTTGGTTCTGCCTAATACTTCATCCCGCCGTCG

**.***.*********.*** ****** *******.** ** *****************

CL2883.CONTIG1_ALL GGTCAAGGGATTCACTGATGTCAACATTGCAGAGAAAAGGGCAAATGTGGAATTTTCTTG

CL2883.CONTIG4_ALL AGTCAAGGGATTCGCTGATGTCAACATTGCAGAGAAAAGGGCAAATGTGGAATTTTCTTG

CL2883.CONTIG5_ALL AGTCAAGGGATTCGCTGATGTCAACATTGCAGAGAAAAGGGCAAATGTGGAATTTTCTTG

XM_027379601 AGTCAAGGGATTCGCTGATGTCAACATTGCAGAGAAAAGGGCAAATGTGGAGTTTTCTTG

AY321153.2 AGTCAAGGGATTCGCTGATGTCAACATTGCAGAGAAAAGGGCAAATGTGGAGTTTTCTTG

CL2833.CONTIG3 GGTCAAGGGATTCACTGATGTCAACATTGCAGAGAAAAGGGCAAATGTGGAATTTTCTTG

CL2883.CONTIG2_ALL GGTCAAGGGATTCACTGATGTCAACATTGCAGAGAAAAGGGCAAATGTGGAGTTTTCTTG

.************.*************************************.********

CL2883.CONTIG1_ALL GGATGCCGATAATGCTCCCGAAAAGAAGTTGGTGTTGGATGCAAGTCTGATCAGCAGTCC

CL2883.CONTIG4_ALL GGATGCCGATAATGCTCCCGAAAAGAAGTTGGTGTTGGATGCAAGTCTGATCAGCAGTCC

CL2883.CONTIG5_ALL GGATGCCGATAATGCTCCCGAAAAGAAGTTGGTGTTGGATGCAAGTCTGATCAGCAGTCC

XM_027379601 GGATGCCGATAATGCTCCCGAAAAGAAGTTGGTGTTGGATGCAAGTCTGATCAGCAGTCC

AY321153.2 GGATGCCGATAATGCTCCCGAAAAGAAGTTGGTGTTGGATGCAAGTCTGATCAGCAGTCC

CL2833.CONTIG3 GGATGCCGATAATGCTCCCGAAAAGAAGTTGGTGTTGGATGCAAGTCTGATCAGCAGTCC

CL2883.CONTIG2_ALL GGATGCCGATAATGCTCCCGAAAAGAAGTTGGTGTTGGATGCAAGTCTGATCAGCAGTCC

************************************************************

CL2883.CONTIG1_ALL TGCCAACCCTGGACATGCTGAGATCCACGGGAATGTCGTCATTGCCGGAGAGCCTTACCA

CL2883.CONTIG4_ALL TGCCAACCCTGGACATGCTGAGATCCACGGGAATGTCGTCATTGCCGGAGAGCCTTACCA

CL2883.CONTIG5_ALL TGCCAACCCTGGACATGCTGAGATCCACGGGAATGTCGTCATTGCCGGAGAGCCTTACCA

XM_027379601 TGCCAACCCTGGACATGCTGAGATCCACGGGAATGTCGTCATTGCCGGAGAGCCTTACCA

AY321153.2 TGCCAACCCTGGACATGCTGAGATCCACGGGAATGTCGTCATTGCCGGAGAGCCTTACCA

CL2833.CONTIG3 TGCCAACCCTGGACATGCTGAGATCCACGGGAATGTCGTCATTGCCGGAGAGCCTTACCA

CL2883.CONTIG2_ALL TGCCAACCCTGGACATGCTGAGATCCACGGGAATGTCGTCATTGCCGGAGAGCCTTACCA

************************************************************

CL2883.CONTIG1_ALL CGCCAAATTGATTCTGACTGCCACAGATCTCATAGAGCACATGGAAGGGGAAAATGGATT

CL2883.CONTIG4_ALL CGCCAAACTGGTTCTGACTGCCACAAATCTCGTAGAGCACATGGAAGGGGAAAATGGATT

CL2883.CONTIG5_ALL CGCCAAACTGGTTCTGACTGCCACAAATCTCGTAGAGCACATGGAAGGGGAAAATGGATT

XM_027379601 CGCCAAACTGGTTCTGACTGCCACAAATCTCGTAGAGCACATGGAAGGGGAAAATGGATT

AY321153.2 CGCCAAACTGGTTCTGACTGCCACAAATCTCGTAGAGCACATGGAAGGGGAAAATGGATT

CL2833.CONTIG3 CGCCAAATTGATTCTGACTGCCACAGATCTCATAGAGCACATGGAAGGGGAAAATGGATT

CL2883.CONTIG2_ALL CGCCAAATTGATTCTGACTGCCACAGATCTCATAGAGCACATGGAAGGGGAAAATGGATT

******* **.**************.*****.****************************

CL2883.CONTIG1_ALL CAAGTTGATCCTGACAACTCCTAGCCAGAAGACGGTTGTCGTGGGAGCCTCCTGTGATGT

CL2883.CONTIG4_ALL CAAGTTGATCCTGACAACTCCTAGCCAGAAGACGGTTGTCGTGGGAGCCTCCTGTGATGT

CL2883.CONTIG5_ALL CAAGTTGATCCTGACAACTCCTAGCCAGAAGACGGTTGTCGTGGGAGCCTCCTGTGATGT

XM_027379601 CAAGTTGATCCTGACAACTCCTAGCCAGAAGACGGTTGTCGTGGGAGCCTCCTGTGATGT

AY321153.2 CAAGTTGATCCTGACAACTCCTAGCCAGAAGACGGTTGTCGTGGGAGCCTCCTGTGATGT

CL2833.CONTIG3 CAAGTTGATCCTGACAACTCCTAGCCAGAAGACGGTTGTCGTGGGAGCCTCCTGTGATGT

CL2883.CONTIG2_ALL CAAGTTGATCCTGACAACTCCTAGCCAGAAGACGGTTGTCGTGGGAGCCTCCTGTGATGT

************************************************************

CL2883.CONTIG1_ALL CCAGCTGGCAGGAGCCACCACTAAAGTCATTTCCACCGTTGAATACAAGAACGTGAGGGA

CL2883.CONTIG4_ALL CCAGCTGGCAGGAACCACCACTAAAGTCCTTTCCACCGTTGAATACAAGAACGTGAGGGA

CL2883.CONTIG5_ALL CCAGCTGGCAGGAGCCACCACTAAAGTCATTTCCACCGTTGAATACAAGAACGTGAGGGA

XM_027379601 CCAGCTGGCAGGAGCCACCACTAAAGTCATTTCCACCGTTGAATACAAGAACGTGAGGGA

AY321153.2 CCAGCTGGCAGGAACCACCACTAAAGTCCTTTCCACCGTTGAATACAAGAACGTGAGGGA

CL2833.CONTIG3 CCAGCTGGCAGGAACCACCACTAAAGTCCTTTCCACCGTTGAATACAAGAACGTGAGGGA

CL2883.CONTIG2_ALL CCAGCTGGCAGGAGCCACCACTAAAGTCATTTCCACCGTTGAATACAAGAACGTGAGGGA

*************.**************.*******************************

CL2883.CONTIG1_ALL TAGGAAATACAAATATACAAGTGTGATTGCCTTGGAGAGGCTTGGTGGTCCACTTAATTA

CL2883.CONTIG4_ALL TAGGAAATACAAATATACAAGTGTGATTGCCTTGGAGAGGCTTGGTGGTCCACTTAATTA

CL2883.CONTIG5_ALL TAGGAAATACAAATATACAAGTGTGATTGCCTTGGAGAGGCTTGGTGGTCCACTTAATTA

XM_027379601 TAGGAAATACAAATATACAAGTGTGATTGCCTTGGAGAGGCTTGGTGGTCCACTTAATTA

AY321153.2 TAGGAAATACAAATATACAAGTGTGATTGCCTTGGAGAGGCTTGGTGGTCCACTTAATTA

CL2833.CONTIG3 TAGGAAATACAAATATACAAGTGTGATTGCCTTGGAGAGGCTTGGTGGTCCACTTAATTA

CL2883.CONTIG2_ALL TAGGAAATACAAATATACAAGTGTGATTGCCTTGGAGAGGCTTGGTGGTCCACTTAATTA

************************************************************

CL2883.CONTIG1_ALL TGCCGTAGAAGCCAAGGTAACTTACAAACAACCTGGAACAGCAGAAATAAAGGTAGAAAC

CL2883.CONTIG4_ALL TGCCGTAGAAGCCAAGGTAACTTACAAACAACCTGGAACAGCAGAAATAAAGGTAGAAAC

CL2883.CONTIG5_ALL TGCCGTAGAAGCCAAGGTAACTTACAAACAACCTGGAACAGCAGAAATAAAGGTAGAAAC

XM_027379601 TGCCGTAGAAGCCAAGGTAACTTACAAACAACCTGGAACAGCAGAAATAAAGGTAGAAAC

AY321153.2 TGCCGTAGAAGCCAAGGTAACTTACAAACAACCTGGAACAGCAGAAATAAAGGTAGAAAC

CL2833.CONTIG3 TGCCGTAGAAGCCAAGGTAACTTACAAACAACCTGGAACAGCAGAAATAAAGGTAGAAAC

CL2883.CONTIG2_ALL TGCCGTAGAAGCCAAGGTAACTTACAAACAACCTGGAACAGCAGAAATAAAGGTAGAAAC

************************************************************

CL2883.CONTIG1_ALL AACAGCAAAACATCATTGGACACCAGAAGAACATGTTGTAGCATTCAAGGTGGCTGCTGA

CL2883.CONTIG4_ALL AACAGCAAAACATCATTGGACACCAGAAGAACATGTTGTAGCATTCAAGGTGGCTGCTGA

CL2883.CONTIG5_ALL AACAGCAAAACATCATTGGACACCAGAAGAACATGTTGTAGCATTCAAGGTGGCTGCTGA

XM_027379601 AACAGCAAAACATCATTGGACACCAGAAGAACATGTTGTAGCATTCAAGGTGGCTGCTGA

AY321153.2 AACAGCAAAACATCATTGGACACCAGAAGAACATGTTGTAGCATTCAAGGTGGCTGCTGA

CL2833.CONTIG3 AACAGCAAAACATCATTGGACACCAGAAGAACATGTTGTAGCATTCAAGGTGGCTGCTGA

CL2883.CONTIG2_ALL AACAGCTAAACATCATTGGACACCAGAAGAACATGTTGTAGCATTCAAGGTGGCTGCTGA

******:*****************************************************

CL2883.CONTIG1_ALL AGCTCCAGTACTGAAGACGCCTGCCATGATTGCATTCTCCATTCACAATGCACCAAACGC

CL2883.CONTIG4_ALL AGCTCCAGTACTGAAGACGCCTGCCATGATTGCATTCTCCATTCACAATGCACCAAACGC

CL2883.CONTIG5_ALL AGCTCCAGTACTGAAGACGCCTGCCATGATTGCATTCTCCATTCACAATGCACCAAACGC

XM_027379601 AGCTCCAGTACTGAAGACGCCTGCCATGATTGCATTCTCCATTCACAATGCACCAAACGC

AY321153.2 AGCTCCAGTACTGAAGACGCCTGCCATGATTGCATTCTCCATTCACAATGCACCAAACGC

CL2833.CONTIG3 AGCTCCAGTACTGAAGACGCCTGCCATGATTGCATTCTCCATTCACAATGCACCAAACGC

CL2883.CONTIG2_ALL AGCTCCAGTACTGAAGACGCCTGCCATGATTGCATTCTCCATTCACAATGCACCAAACGC

************************************************************

CL2883.CONTIG1_ALL TTTTGTTGGAGTCTGCAAGATCGAAAGAACTGCTCCTTTCACTGCCTTTGAATGGAATGT

CL2883.CONTIG4_ALL TTTTGTTGGAGTCTGCAAGATCGAAAGAACTGCTCCTTTCACTGCCTTTGAATGGAATGT

CL2883.CONTIG5_ALL TTTTGTTGGAGTCTGCAAGATCGAAAGAACTGCTCCTTTCACTGCCTTTGAATGGAATGT

XM_027379601 TTTTGTTGGAGTCTGCAAGATCGAAAGAACTGCTCCTTTCACTGCCTTTGAATGGAATGT

AY321153.2 TTTTGTTGGAGTCTGCAAGATCGAAAGAACTGCTCCTTTCACTGCCTTTGAATGGAATGT

CL2833.CONTIG3 TTTTGTTGGAGTCTGCAAGATCGAAAGAACTGCTCCTTTCACTGCCTTTGAATGGAATGT

CL2883.CONTIG2_ALL TTTTGCTGGAGTCTGCAAGATCGAAAGAACTGCTCCTTTCACTGCCTTTGAATGGAATGT

***** ******************************************************

CL2883.CONTIG1_ALL ACAGGTTACTCCTGAAGGAGGAATTGAAGCTGTTGAAGCTGGTGTGGACATGAAAGCCAT

CL2883.CONTIG4_ALL GCAGGTTACTCCTGAAGGAGGAATTGAAGCTGTTGAAGCTGGTGTGGACATGAAAGCCAT

CL2883.CONTIG5_ALL GCAGGTTACTCCTGAAGGAGGAATTGAAGCTGTTGAAGCTGGTGTGGACATGAAAGCCAT

XM_027379601 GCAGGTTACTCCTGAAGGAGGAATTGAAGCTGTTGAAGCTGGTGTGGACATGAAAGCCAT

AY321153.2 ACAGGTTACTCCTGAAGGAGGAATTGAAGCTGTTGAAGCTGGTGTGGACATGAAAGCCAT

CL2833.CONTIG3 ACAGGTTACTCCTGAAGGAGGAATTGAAGCTGTTGAAGCTGGTGTGGACATGAAAGCCAT

CL2883.CONTIG2_ALL ACAGGTTACTCCTGAAGGAGGAATTGAAGCTGTTGAAGCTGGTGTGGACATGAAAGCCAT

.***********************************************************

CL2883.CONTIG1_ALL CATTGAAGTTCTGAAGATTGTTCGTGCCATTGCTACTCTGGAGGAAGAGAGTTATGAAAC

CL2883.CONTIG4_ALL CATTGAAGTTCTGAAGATTGTTCGTGCCATTGCTACTCTGGAGGAAGAGAGTTATGAAAC

CL2883.CONTIG5_ALL CATTGAAGTTCTGAAGATTGTTCGTGCCATTGCTACTCTGGAGGAAGAGAGTTATGAAAC

XM_027379601 CATTGAAGTTCTGAAGATTGTTCGTGCCATTGCTACTCTGGAGGAAGAGAGTTATGAAAC

AY321153.2 CATTGAAGTTCTGAAGATTGTTCGTGCCATTGCTACTCTGGAGGAAGAGAGTTATGAAAC

CL2833.CONTIG3 CATTGAAGTTCTGAAGATTGTTCGTGCCATTGCTACTCTGGAGGAAGAGAGTTATGAAAC

CL2883.CONTIG2_ALL CATTGAAGTTCTGAAGATTGTTCGTGCCATTGCTACTCTGGAGGAAGAGAGTTATGAAAC

************************************************************

CL2883.CONTIG1_ALL TTATGGCCCACACACAGCTCAGTACCAGTACCGCTTCACAAGGCCATCACCCACTTCTTA

CL2883.CONTIG4_ALL TTATGGCCCACACACAGCTCAGTACCAGTACCGCTTCACAAGGCCATCACCCACTTCTTA

CL2883.CONTIG5_ALL TTATGGCCCACACACAGCTCAGTACCAGTACCGCTTCACAAGGCCATCACCCACTTCTTA

XM_027379601 TTATGGCCCACACACAGCTCAGTACCAGTACCGCTTCACAAGGCCATCACCCACTTCTTA

AY321153.2 TTATGGCCCACACACAGCTCAGTACCAGTACCGCTTCACAAGGCCATCACCCACTTCTTA

CL2833.CONTIG3 TTATGGCCCACACACAGCTCAGTACCAGTACCGCTTCACAAGGCCATCACCCACTTCTTA

CL2883.CONTIG2_ALL TTATGGCCCACACACAGCTCAGTACCAGTACCGCTTCACAAGGCCATCACCCACTTCTTA

************************************************************

CL2883.CONTIG1_ALL CACCATGCAGATGAGGACTCCAACCCGCACCATGGAAGGAAGAGCTAAACTATCACCAAG

CL2883.CONTIG4_ALL CACCATGCAGATGAGGACTCCAACCCGCACCATGGAAGGAAGAGCTAAACTATCACCAAG

CL2883.CONTIG5_ALL CACCATGCAGATGAGGACTCCAACCCGCACCATGGAAGGAAGAGCTAAACTATCACCAAG

XM_027379601 CACCATGCAGATGAGGACTCCAACCCGCACCATGGAAGGAAGAGCTAAACTATCACCAAG

AY321153.2 CACCATGCAGATGAGGACTCCAACCCGCACCATGGAAGGAAGAGCTAAACTATCACCAAG

CL2833.CONTIG3 CACCATGCAGATGAGGACTCCAACCCGCACCATGGAAGGAAGAGCTAAACTATCACCAAG

CL2883.CONTIG2_ALL CACCATGCAGATGAGGACTCCAACCCGCACCATGGAAGGAAGAGCTAAACTATCACCAAG

************************************************************

CL2883.CONTIG1_ALL GGAATCTGGAATCAAGTTCTACCCCAATAAGGGCAAAACTGAATCCAAATACGAAATTGG

CL2883.CONTIG4_ALL GGAATCTGGAATCAAGTTCTACCCCAATAAGGGCAAAACTGAATCCAAATACGAAATTGG

CL2883.CONTIG5_ALL GGAATCTGGAATCAAGTTCTACCCCAATAAGGGCAAAACTGAATCCAAATACGAAATTGG

XM_027379601 GGAATCTGGAATCAAGTTCTACCCCAATAAGGGCAAAACTGAATCCAAATACGAAATTGG

AY321153.2 GGAATCTGGAATCAAGTTCTACCCCAATAAGGGCAAAACTGAATCCAAATACGAAATTGG

CL2833.CONTIG3 GGAATCTGGAATCAAGTTCTACCCCAATAAGGGCAAAACTGAATCCAAATACGAAATTGG

CL2883.CONTIG2_ALL GGAATCTGGAATCAAGTTCTACCCCAATAAGGGCAAAACTGAATCCAAATACGAAATTGG

************************************************************

CL2883.CONTIG1_ALL ATACAAGGTCAACCACGAGGGAAGGTGGGCACAACGTGCGTCCAAGTTGGAAGTCAGAAT

CL2883.CONTIG4_ALL ATACAAGGTCAACCACGAGGGAAGGTGGGCACAACGTGCGTCCAAGTTGGAAGTCAGAAT

CL2883.CONTIG5_ALL ATACAAGGTCAACCACGAGGGAAGGTGGGCACAACGTGCGTCCAAGTTGGAAGTCAGAAT

XM_027379601 ATACAAGGTCAACCACGAGGGAAGGTGGGCACAACGTGCGTCCAAGTTGGAAGTCAGAAT

AY321153.2 ATACAAGGTCAACCACGAGGGAAGGTGGGGACAACGTGCGTCCAAGTTGGAAGTCAGAAT

CL2833.CONTIG3 ATACAAGGTCAACCACGAGGGAAGGTGGGGACAACGTGCGTCCAAGTTGGAAGTCAGAAT

CL2883.CONTIG2_ALL ATACAAGGTCAACCACGAGGGAAGGTGGGGACAACGTGCGTCCAAGTTGGAAGTCAGAAT

***************************** ******************************

CL2883.CONTIG1_ALL GAACCATCCAGTGCTTCCTAAACCCATCATGGCCGCTGCTCAGTACACAGTAGCTGAAGG

CL2883.CONTIG4_ALL GAACCATCCAGTGCTTCCTAAACCCATCATGGCCGCTGCTCAGTACACAGTAGCTGAAGG

CL2883.CONTIG5_ALL GAACCATCCAGTGCTTCCTAAACCCATCATGGCCGCTGCTCAGTACACAGTAGCTGAAGG

XM_027379601 GAACCATCCAGTGCTTCCTAAACCCATCATGGCCGCTGCTCAGTACACAGTAGCTGAAGG

AY321153.2 GAACCATCCAGTGCTTCCTAAACCCATCATGGCCGCTGCTCAGTACACAGTAGCTGAAGG

CL2833.CONTIG3 GAACCATCCAGTGCTTCCTAAACCCATCATGGCCGCTGCTCAGTACACAGTAGCTGAAGG

CL2883.CONTIG2_ALL GAACCATCCAGTGCTTCCTAAACCCATCATGGCCGCTGCTCAGTACACAGTAGCTGAAGG

************************************************************

CL2883.CONTIG1_ALL AACAATGAGGGGAACAATTGAACTGGACATTTTCCCAGAAGAAGCCGACAAAATTACTGG

CL2883.CONTIG4_ALL AACAATGAGGGGAACAATTGAACTGGACATTTTCCCAGAAGAAGCCGACAAAATTACTGG

CL2883.CONTIG5_ALL AACAATGAGGGGAACAATTGAACTGGACATTTTCCCAGAAGAAGCCGACAAAATTACTGG

XM_027379601 AACAATGAGGGGAACAATTGAACTGGACATTTTCCCAGAAGAAGCCGACAAAATTACTGG

AY321153.2 AACAATGAGGGGAACAATTGAACTGGACATTTTCCCAGAAGAAGCCGACAAAATTACTGG

CL2833.CONTIG3 AACAATGAGGGGAACAATTGAACTGGACATTTTCCCAGAAGAAGCCGACAAAATTACTGG

CL2883.CONTIG2_ALL AACAATGAGGGGAACAATTGAACTGGACATTTTCCCAGAAGAAGCCGACAAAATTACTGG

************************************************************

CL2883.CONTIG1_ALL AACTGTGGAAACTCAGAGAATTTCAGAAAATGCTATCAGGGCAGAAGCCTTCTTGACTGG

CL2883.CONTIG4_ALL AACTGTGGAAACTCAGAGAATTTCAGAAAATGCTATCAGGGCAGAAGCCTTCTTGACTGG

CL2883.CONTIG5_ALL AACTGTGGAAACTCAGAGAATTTCAGAAAATGCTATCAGGGCAGAAGTCTTCTTGACTGG

XM_027379601 AACTGTGGAAACTCAGAGAATTTCAGAAAATGCTATCAGGGCAGAAGCCTTCTTGACTGG

AY321153.2 AACTGTGGAAACTCAGAGAATTTCAGAAAATGCTATCAGGGCAGAAGTCTTCTTGACTGG

CL2833.CONTIG3 AACTGTGGAAACTCAGAGAATTTCAGAAAATGCTATCAGGGCAGAAGCCTTCTTGACTGG

CL2883.CONTIG2_ALL AACTGTGGAAACTCAGAGAATTTCAGAAAATGCTATCAGGGCAGAAGCCTTCTTGACTGG

*********************************************** ************

CL2883.CONTIG1_ALL TAGGATGTTGAAAGTGAACCCTAAAGCTATCATCACTGCTGCCTATGCACCAGAAACAGT

CL2883.CONTIG4_ALL TAGGATGTTGAAAGTGAACCCTAAAGCTATCATCACTGCTGCCTATGCACCAGAAACAGT

CL2883.CONTIG5_ALL CAGGATGTTGAAAGTGAACCCTAAGGCTATCATCACTGCTGCCTATGCACCAGAAACAGT

XM_027379601 CAGGATGTTGAAAGTGAACCCTAAAGCTATCATCACTGCTGCCTATGCACCAGAAACAGT

AY321153.2 CAGGATGTTGAAAGTGAACCCTAAGGCTATCATCACTGCTGCCTATGCACCAGAAACAGT

CL2833.CONTIG3 TAGGATGTTGAAAGTGAACCCTAAAGCTATCATCACTGCTGCCTATGCACCAGAAACAGT

CL2883.CONTIG2_ALL TAGGATGTTGAAAGTGAACCCTAAAGCTATCATCACTGCTGCCTATGCACCAGAAACAGT

***********************.***********************************

CL2883.CONTIG1_ALL TGCTTTGGATGTAGTGTTCCACAAGACTCCGTCTGCAGCACCAATCTTCGCCATTGCTGC

CL2883.CONTIG4_ALL TGCTTTGGATGTAGTGTTCCACAAGACTCCGTCTGCAGCACCAATCTTCGCCATTGCTGC

CL2883.CONTIG5_ALL TGCTTTGGATGTAGTGTTCCACAAGACTCCGTCTGCAGCACCAATCTTCGCCATTGCTGC

XM_027379601 TGCTTTGGATGTAGTGTTCCACAAGACTCCATCTGCAGCACCAATCTTCGCCATTGCTGC

AY321153.2 TGCTTTGGATGTAGTGTTCCACAAGACTCCGTCTGCAGCACCAATCTTCGCCATTGCTGC

CL2833.CONTIG3 TGCTTTGGATGTAGTGTTCCACAAGACTCCATCTGCAGCACCAATCTTCGCCATTGCTGC

CL2883.CONTIG2_ALL TGCTTTGGATGTAGTGTTCCACAAGACTCCGTCTGCAGCACCAATCTTCGCCATTGCTGC

******************************.*****************************

CL2883.CONTIG1_ALL CAAGTATGACAAGACTGCAGCTCACAGTGCAGCTGCCACATTGACAGTAAAGATGGAAGA

CL2883.CONTIG4_ALL CAAGTATGACAAGACTGCAGCTCACAGTGCAGCTGCCACATTGACAGTAAAGATGGAAGA

CL2883.CONTIG5_ALL CAAGTATGACAAGACTGCAGCTCACAGTGCAGCTGCCACATTGACAGTAAAGATGGAAGA

XM_027379601 CAAGTATGACAAGACTGCAGCTCACAGTGCAGCTGCCACATTGACAGTAAAGATGGAAGA

AY321153.2 CAAGTATGACAAGACTGCAGCTCACAGTGCAGCTGCCACATTGACAGTAAAGATGGAAGA

CL2833.CONTIG3 CAAGTATGACAAGACTGCAGCTCACAGTGCAGCTGCCACATTGACAGTAAAGATGGAAGA

CL2883.CONTIG2_ALL CAAGTATGACAAGACTGCAGCTCACAGTGCAGCTGCCACATTGACAGTAAAGATGGAAGA

************************************************************

CL2883.CONTIG1_ALL GCGACCTGTCTTTGAGATGAGTGCAGTGACCGAACCCGAGGAACCAGCCACCTGCAATGG

CL2883.CONTIG4_ALL GCGACCTGTCTTTGAGATGAGTGCAGTGACCGAACCCGAGGAACCAGCCACCTGCAATGG

CL2883.CONTIG5_ALL GCGACCTGTCTTTGAGATGAGTGCAGTGACCGAACCCGAGGAACCAGCCACCTGCAATGG

XM_027379601 GCGACCTGTCTTTGAGATGAGTGCAGTGACCGAACCCGAGGAAGCAGCCACCTGCAATGG

AY321153.2 GCGACCTGTCTTTGAGATGAGTGCAGTGACCGAACCTGAGGAAGCAGCCACCTGCAATGG

CL2833.CONTIG3 GCGACCTGTCTTTGAGATGAGTGCAGTGACCGAACCCGAGGAACCAGCCACCTGCAATGG

CL2883.CONTIG2_ALL GCGACCTGTCTTTGAGATGAGTGCAGTGACCGAACCCGAGGAACCAGCCACCTGCAATGG

************************************ ****** ****************

CL2883.CONTIG1_ALL CATCAGAATGAATGCTGTTGCTTATGCAGCAGCTTTTGGAAAGTACAACGTGTTCTCCAA

CL2883.CONTIG4_ALL CATCAGAATGAATGCTGTTGCTTATGCAGCAGCTTTTGGAAAGTACAACGTGTTCTCCAA

CL2883.CONTIG5_ALL CATCAGAATGAATGCTGTTGCTTATGCAGCAGCTTTTGGAAAGTACAACGTGTTCTCCAA

XM_027379601 CATCAGAATGAATGCTGTTGCTTATGCAGCAGCTTTTGGAAAGTACAACGTGTTCTCCAA

AY321153.2 CATCAGAATGAATGCTGTTGCTTATGCAGCAGCTTTTGGAAAGTACAACGTGTTCTCCAA

CL2833.CONTIG3 CATCAGAATGAATGCTGTTGCTTATGCAGCAGCTTTTGGAAAGTACAACGTGTTCTCCAA

CL2883.CONTIG2_ALL CATCAGAATGAATGCTGTTGCTTATGCAGCAGCTTTTGGAAAGTACAACGTGTTCTCCAA

************************************************************

CL2883.CONTIG1_ALL GATGTGCAGGCCCGCCTTCATTGAGGTGACCGCAATGCGACCTGGTGGAGCAAAGGAGTA

CL2883.CONTIG4_ALL GATGTGCAGGCCCGCCTTCATTGAGGTGACCGCAATGCGACCTGGTGGAGCAAAGGAGTA

CL2883.CONTIG5_ALL GATGTGCAGGCCCGCCTTCATTGAGGTGACCGCAATGCGACCTGGTGGAGCAAAGGAGTA

XM_027379601 GATGTGCAGGCCCGCCTTCATTGAGGTGACCGCAATGCGACCTGGTGGAGCAAAGGAGTA

AY321153.2 GATGTGCAGGCCCGCCTTCATTGAGGTGACCGCAATGCGACCTGGTGGAGCAAAGGAGTA

CL2833.CONTIG3 GATGTGCAGGCCCGCCTTCATTGAGGTGACCGCAATGCGACCTGGTGGAGCAAAGGAGTA

CL2883.CONTIG2_ALL GATGTGCAGGCCCGCCTTCATTGAGGTGACCGCAATGCGACCTGGTGGAGCAAAGGAGTA

************************************************************

CL2883.CONTIG1_ALL CACTGCCAAGCTTGGCCTCCGATACCCTGACGCTGCTGAAGCAGGCGTATATGTGGCGAG

CL2883.CONTIG4_ALL CACTGCCAAGCTTGGCCTCCGATACCCTGACGCTGCTGAAGCAGGCGTATATGTGGCGAG

CL2883.CONTIG5_ALL CACTGCCAAGCTTGGCCTCCGATACCCTGACGCTGCTGAAGCAGGCGTATATGTGGCGAG

XM_027379601 CACTGCCAAGCTTGGCCTCCGATACCCTGACGCTGCTGAAGCAGGCGTATATGTGGCGAG

AY321153.2 CACTGCCAAGCTTGGCCTCCGATACCCTGACGCTGCTGAAGCAGGCGTATATGTGACGAG

CL2833.CONTIG3 CACTGCCAAGCTTGGCCTCCGATACCCTGACGCTGCTGAAGCAGGCGTATATGTGGCGAG

CL2883.CONTIG2_ALL CACTGCCAAGCTTGGCCTCCGATTCCCTGACGCTGCTGAAGCAGGCGTATATGTGGCGAG

***********************:*******************************.****

CL2883.CONTIG1_ALL TGGCAGAGCTGGAGAGAGTCGCGGTGTTGCTGTTGCTGCTGTGAAGCTGGCTTCACCCAC

CL2883.CONTIG4_ALL TG------CTGGAGAGAGTCGCGGTGTTGCTGTTGCTGCTGTGAAGCTGGCTTCACCCAC

CL2883.CONTIG5_ALL TGGCAGAGCTGGAGAGAGTCGCGGTGTTGCTGTTGCTGCTGTGAAGCTGGCTTCACCCAA

XM_027379601 TG------CTGGAGAGAGTCGCGGTGTTGCTGTTGCTGCTGTGAAGCTGGCTTCACCCAC

AY321153.2 TGGCAGAGCTGGAGAGAGTCGCGGTGTTGCTGTTGCTGCTGTGAAGCTGGCTTCACCCAA

CL2833.CONTIG3 TGGCAGAGCTGGAGAGAGTCGCGGTGTTGCTGTTGCTGCTGTGAAGCTGGCTTCACCCAC

CL2883.CONTIG2_ALL TG------CTGGAGAGAGTCGCGGTGTTGCTGTTGCTGCTGTGAAGCTGGCTTCACCCAA

** ***************************************************.

CL2883.CONTIG1_ALL AATGCTACAGTTCGAGGTGGCTCATGAACCAGAAGAAGCACACATTGTAATGAGTGAAGT

CL2883.CONTIG4_ALL AATGCTACAGTTCGAGGTGGCTCATGAACCAGAAGAAGCACACATTGTAATGAGTGAAGT

CL2883.CONTIG5_ALL AGTACTGAAAGTCGAGATGGCTTATGAGCCGCAAGAAGCAGAAGCAATAAGCATTGAAAT

XM_027379601 AATGCTACAGTTCGAGGTGGCTCATGAACCAGAAGAAGCACACATTGTAATGAGTGAAGT

AY321153.2 AGTACTGAAAGTCGAGATGGCTTATGAGCCGCAAGAAGCAGAAGCAATAAGCATTGAAAT

CL2833.CONTIG3 AATGCTACAGTTCGAGGTGGCTCATGAACCAGAAGAAGCACACATTGTAATGAGTGAAGT

CL2883.CONTIG2_ALL AGTACTGAAAGTCGAGATGGCTTATGAGCCGCAAGAAGCAGAAGCAATAAGCATTGAAAT

*.*.**..*. *****.***** ****.**. ******** *.. :.*** * ****.*

CL2883.CONTIG1_ALL GACAAGTACCCTCAGAAAAGTCGCCATGTCTCTCGAAACAGTTGCAATGGAGGCCGTCCA

CL2883.CONTIG4_ALL GACAAGTACCCTCAGAAAAGTCGCCATGTCTCTCGAAACAGTTGCAATGGAGGCCGTCCA

CL2883.CONTIG5_ALL GACTGAAGGATTTGAGAAGATTGCTGTATCATTCAAGTCTGTTGCAATGGAGTTCGTCCA

XM_027379601 GACAAGTACCCTCAGAAAAGTCGCCATGTCTCTCGAAACAGTTGCAATGGAGGCCGTCCA

AY321153.2 GACTGAAGGATTTGAGAAGATTGCTGTATCATTCAAGTCTGTTGCAATAGAGTTCGTCCA

CL2833.CONTIG3 GACAAGTACCCTCAGAAAAGTCGCCATGTCTCTCGAAACAGTTGCAATGGAGGCCGTCCA

CL2883.CONTIG2_ALL GACTGAAGGATTTGAGAAGATTGCTGTATCATTCAAGTCTGTTGCAATGGAGTTCGTCCA

***:..:. . * ...**..* ** .*.**: **.*.:*:********.*** ******

CL2883.CONTIG1_ALL GTTCCTCAAGGAAGAAGCTGCTGCAAAGGGTGTCGAGTTCCCGTCATCTCACTTTGTCAG

CL2883.CONTIG4_ALL GTTCCTCAAGGAAGAAGCTGCTGCAAAGGGTGTCGAGTTCCCGTCATCTCACTTTGTCAG

CL2883.CONTIG5_ALL ATTCCTCAAGGAAGAGGCTGCTGCAAAGGGTGTTCAGTTCTCTTCATCTCAGTTAGTCAA

XM_027379601 GTTCCTCAAGGAAGAAGCTGCTGCAAAGGGTGTCGAGTTCCCGTCATCTCACTTTGTCAG

AY321153.2 ATTCCTCAAGGAAGAGGCTGCTGCAAAGGGTGTTCAGTTCTCTTCATCTCAGTTAGTCAA

CL2833.CONTIG3 GTTCCTCAAGGAAGAAGCTGCTGCAAAGGGTGTCGAGTTCCCGTCATCTCACTTTGTCAG

CL2883.CONTIG2_ALL ATTCCTCAAGGAAGAGGCTGCTGCAAAGGGTGTTCAGTTCTCTTCATCTCAGTTAGTCAA

.**************.***************** ***** * ******** **:****.

CL2883.CONTIG1_ALL TCTAGTGGATGAGGCGAATGAGGAAATCAAAGCCATTTACCGAGATATCGTCTCAGAGAT

CL2883.CONTIG4_ALL TCTAGTGGATGAGGCGAATGAGGAAATCAAAGCCATTTACCGAGATATCGTCTCAGAGAT

CL2883.CONTIG5_ALL TTTAATGGGAGTTGCTAAGGAGGAAATTGTAGAGATCTATCGAGATATTCTCTCCGAGGC

XM_027379601 TCTAGTGGATGAGGCGAATGAGGAAATCAAAGCCATTTACCGAGATATCGTCTCAGAGAT

AY321153.2 TCTAATGGGAGTTGCTAAGGAGGAAATTGTAGAGATCTATCGAGATATTCTCTCCGAGGC

CL2833.CONTIG3 TCTAGTGGATGAGGCGAATGAGGAAATCAAAGCCATTTACCGAGATATCGTCTCAGAGAT

CL2883.CONTIG2_ALL TTTAATGGGAGTTGCTAAGGAGGAAATTGTAGAGATCTATCGAGATATTCTCTCCGAGGC

* **.***.:*: ** ** ******** .:**. ** ** ******** ****.***.

CL2883.CONTIG1_ALL GAGAATCCTTGACACTGAGTTGATTGCTGATATCTTGGAAAGCCCTACGGTGTCCTTCGT

CL2883.CONTIG4_ALL GAGAATCCTTGACACTGAGTTGATTGCTGATATCTTGGAAAGCCCTACGGTGTCCTTCGT

CL2883.CONTIG5_ALL AAGAATTTTTCATACCGAAATCCTTGCTAATATTCTGGAAAGTCCTGTGGTATCCGTCAT

XM_027379601 GAGAATCCTTGACACTGAGTTGATTGCTGATATCTTGGAAAGCCCTACGGTGTCCTTCGT

AY321153.2 AAGAATTTTTCATACCGAAATCCTTGCTAATATTCTGGAAAGTCCTGTGGTATCCGTCAT

CL2833.CONTIG3 GAGAATCCTTGACACTGAGTTGATTGCTGATATCTTGGAAAGCCCTACGGTGTCCTTCGT

CL2883.CONTIG2_ALL AAGAATTTTTCATACCGAAATCCTTGCTAATATTCTGGAAAGTCCTGTGGTATCCGTCAT

.***** ** * ** **.:* .*****.**** ******* ***. ***.*** **.*

CL2883.CONTIG1_ALL GTCGCGTGTCTACCTTGGAGTATGGTCACAGATTGCTCGCCTTCAACATCACTTTTCAAC

CL2883.CONTIG4_ALL GTCGCGTGTCTACCTTGGAGTATGGTCACAGATTGCTCGCCTTCAACATCACTTTTCAAC

CL2883.CONTIG5_ALL ATCACGAGTCTACTTCGGTGTGCGGTCGGAAATTGTTCGCCTTCAACACCAGCTTTCCGT

XM_027379601 GTCGCGTGTCTACCTTGGAGTATGGTCACAGATTGCTCGCCTTCAACATCACTTTTCAAC

AY321153.2 ATCACGAGTCTACTTCGGTGTGCGGTCGGAAATTGTTCGCCTTCAACACCAGCTTTCCGT

CL2833.CONTIG3 GTCGCGTGTCTACCTTGGAGTATGGTCACAGATTGCTCGCCTTCAACATCACTTTTCAAC

CL2883.CONTIG2_ALL ATCACGAGTCTACTTCGGTGTGCGGTCGGAAATTGTTCGCCTTCAACACCAGCTTTCCGT

.**.**:****** * **:**. ****. *.**** ************ ** ****..

CL2883.CONTIG1_ALL CAGGGCGGTTGAAATGATCCAGCAATGGCAGGAACAACTAACAGACGTTTCTGAAATCTT

CL2883.CONTIG4_ALL CAGGGCGGTTGAAATGATCCAGCAATGGCAGGAACAACTAACAGACGTTTCTGAAATCTT

CL2883.CONTIG5_ALL AACCCTCATCCAGGCGATAGAAGGAGGCCAGGAGGAATTAGCAATCGTTTATGAAATCGT

XM_027379601 CAGGGCGGTTGAAATGATCCAGCAATGGCAGGAACAACTAACAGACGTTTCTGAAATCTT

AY321153.2 AACCCTCATCCAGGCGATAGAAGGAGGCCAAGAGGAATTAGCAATCGTTTATGAAATCGT

CL2833.CONTIG3 CAGGGCGGTTGAAATGATCCAGCAATGGCAGGAACAACTAACAGACGTTTCTGAAATCTT

CL2883.CONTIG2_ALL AACCCTCATCCAGGCGATAGAAGGAGGCCAGGAGGAATTAGCAATCGTTTATGAAATCGT

.* .* *.. ***. *. .* * **.**. ** **.**.:*****.******* *

CL2883.CONTIG1_ALL TATCGAAGCTGTTATGGAGATAGTGCAACTCTTGGAAGCCGGAGAAGTACCTGAAACAGT

CL2883.CONTIG4_ALL TATCGAAGCTGTTATGGAGATAGTGCAACTCTTGGAAGCCGGAGAAGTACCTGAAACAGT

CL2883.CONTIG5_ALL AATGGAAGTTGTGATGACGGCAGCACGCATGGCAGAAACTGGAGAAGTCCCTGTGGCAGT

XM_027379601 TATCGAAGCTGTTATGGAGATAGTGCAACTCTTGGAAGCCGGAGAAGTACCTGAAACAGT

AY321153.2 AATGGAAGTTGTGATGACGGCAGCACGCATGGCAGAAACTGGAGAAGTCCCTGTGGCAGT

CL2833.CONTIG3 TATCGAAGCTGTTATGGAGATAGTGCAACTCTTGGAAGCCGGAGAAGTACCTGAAACAGT

CL2883.CONTIG2_ALL AATGGAAGTTGTGATGACGGCAGCACGCATGGCAGAAACTGGAGAAGTCCCTGTGGCAGT

:** **** *** ***..*. ** .*...* .***.* ********.****:..****

CL2883.CONTIG1_ALL TCGTGTAATTCTGGAAAAAATTGAGAACACTGAGGTGTTCAGGATTGTAAAGAGAGAAGT

CL2883.CONTIG4_ALL TCGTGTAATTCTGGAAAAAATTGAGAACACTGAGGTGTTCAGGATTGTAAAGAGAGAAGT

CL2883.CONTIG5_ALL GCTTGAGGCACTTGAGGAAATCAAAGCCAGCAAAGCTTTCAGGGTTGTGAAGAGAGAAGT

XM_027379601 TCGTGTAATTCTGGAAAAAATTGAGAACACTGAGGTGTTCAGGATTGTAAAGAGAGAAGT

AY321153.2 GCTTGAGGCACTTGAGGAAATCAAAGCCAGCAAAGCTTTCAGGGTTGTGAAGAGAGAAGT

CL2833.CONTIG3 TCGTGTAATTCTGGAAAAAATTGAGAACACTGAGGTGTTCAGGATTGTAAAGAGAGAAGT

CL2883.CONTIG2_ALL GCTTGAGGCACTTGAGGAAATCAAAGCCAGCAAAGCTTTCAGGGTTGTGAAGAGAGAAGT

* **:.. :** **..**** .*...** .*.* ******.****.***********

CL2883.CONTIG1_ALL GAACGCAGTGTTGGCAGAGTATCCTGAGGAGTATGAGGCCGTCAAGCACATCCTCACCAG

CL2883.CONTIG4_ALL GAACGCAGTGTTGGCAGAGTATCCTGAGGAGTATGAGGCCGTCAAGCACATCCTCACCAG

CL2883.CONTIG5_ALL GGATGTCATTTTGAGAGAATATCCGGAGGAGTATGAAGCTGTCAAGCACATCTTTGGCAA

XM_027379601 GAACGCAGTGTTGGCAGAGTATCCTGAGGAGTATGAGGCCGTCAAGCACATCCTCACCAG

AY321153.2 GGATGTCATTTTGAGAGAATATCCGGAGGAGTATGAAGCTGTCAAGCACATCTTTGGCAA

CL2833.CONTIG3 GAACGCAGTGTTGGCAGAGTATCCTGAGGAGTATGAGGCCGTCAAGCACATCCTCACCAG

CL2883.CONTIG2_ALL GGATGTCATTTTGAGAGAATATCCGGAGGAGTATGAAGCTGTCAAGCACATCTTTGGCAA

*.* * ..* ***. ***.***** ***********.** ************ * . **.

CL2883.CONTIG1_ALL GGTGACGGCCACTCTCAAGCACGATGCTGATATTGTGTACAAGAGGATCATGGAGACACC

CL2883.CONTIG4_ALL GGTGACGGCCACTCTCAAGCACGATGCTGATATTGTGTACAAGAGGATCATGGAGACACC

CL2883.CONTIG5_ALL CGTGGTGGCAATTCTCAAGCGAGATGTTGGCATTGTTCGTGAGTGGCTCATGGAGATTCC

XM_027379601 GGTGACGGCCACTCTCAAGCACGATGCTGATATTGTGTACAAGAGGATCATGGAGACACC

AY321153.2 CGTGGTGGCAATTCTCAAGCGAGATGTTGGCATTGTTCGTGAGTGGCTCATGGAGATTCC

CL2833.CONTIG3 GGTGACGGCCACTCTCAAGCACGATGCTGATATTGTGTACAAGAGGATCATGGAGACACC

CL2883.CONTIG2_ALL CGTGGTGGCAATTCTCAAGCGAGATGTTGGCATTGTTCGTGAGTGGCTCATGGAGATTCC

***. ***.* ********..**** **. ***** . .**:**.********* :**

CL2883.CONTIG1_ALL AGCTGTTCAAAGGATCTTTGCCTATGTTATGCAGTACATCAACTCGGAGCGCGTGTTTGC

CL2883.CONTIG4_ALL AGCTGTTCAAAGGATCTTTGCCTATGTTATGCAGTACATCAACTCGGAGCGCGTGTTTGC

CL2883.CONTIG5_ALL AGCTGTTCAGAGAGTCATCGACTACACCATGTATCACTTCCATTCGGAACGAGCATTTGC

XM_027379601 AGCTGTTCAAAGGATCTTTGCCTATGTTATGCAGTACATCAACTCGGAGCGCGTGTTTGC

AY321153.2 AGCTGTTCAGAGAGTCATCGACTACACCATGTATCACTTCCATTCGGAACGAGCATTTGC

CL2833.CONTIG3 AGCTGTTCAAAGGATCTTTGCCTATGTTATGCAGTACATCAACTCGGAGCGCGTGTTTGC

CL2883.CONTIG2_ALL AGCTGTTCAGAGAGTCATCGACTACACCATGTATCACTTCCATTCGGAACGAGCATTTGC

*********.**..**:* *.*** . *** * **:**.* *****.**.* .*****

CL2883.CONTIG1_ALL TGAGGAAGCAGGAAGTGTTGCCAGCCTCATTCTCAAAGAATTTCTTTTCGTTTCAATTGA

CL2883.CONTIG4_ALL TGAGGAAGCAGGAAGTGTTGCCAGCCTCATTCTCAAAGAATTTCTTTTCGTTTCAATTGA

CL2883.CONTIG5_ALL TGCAGAAGCAGAAAAGGTCGTTCGCCTCATTCTCGACGAACTTCTCTTCGTTTCAATGGA

XM_027379601 TGAGGAAGCAGGAAGTGTTGCCAGCCTCATTCTCAAAGAATTTCTTTTCGTTTCAATTGA

AY321153.2 TGCAGAAGCAGAAAAGGTCGTTCGCCTCATTCTCGACGAACTTCTCTTCGTTTCAATGGA

CL2833.CONTIG3 TGAGGAAGCAGGAAGTGTTGCCAGCCTCATTCTCAAAGAATTTCTTTTCGTTTCAATTGA

CL2883.CONTIG2_ALL TGCAGAAGCAGAAAAGGTCGTTCGCCTCATTCTCGACGAACTTCTCTTCGTTTCAATGGA

**..*******.**. ** * .***********.*.*** **** *********** **

CL2883.CONTIG1_ALL AAGCGAAGGCAACGGCATTGCAGTCCGAATTCCCCTCCACCGACCCTTGTATTCACTGAC

CL2883.CONTIG4_ALL AAGCGAAGGCAACGGCATTGCAGTCCGAATTCCCCTCCACCGACCCTTGTATTCACTGAC

CL2883.CONTIG5_ALL AAGCGAAGGCAACGGCGTTGCAGTCCGAATTCCCCTCCACCGACCCTTGTATTCACTGAC

XM_027379601 AAGCGAAGGCAACGGCATTGCAGTCCGAATTCCCCTCCACCGACCCTTGTATTCACTGAC

AY321153.2 AAGCGAAGGCAACGGCGTTGCAGTCCGAATTCCCCTCCACCGACCCTTTTATTCACTAAC

CL2833.CONTIG3 AAGCGAAGGCAACGGCATTGCAGTCCGAATTCCCCTCCACCGACCCTTGTATTCACTGAC

CL2883.CONTIG2_ALL AAGCGAAGGCAACGGCGTTGCAGTCCGAATTCCCCTCCACCGACCCTTTTATTCACTAAC

****************.******************************* ********.**

CL2883.CONTIG1_ALL GCAAGTGGCACAAGAAGCAGTGCCCAACCCTGTCACAATGCTCGAGAACCTGATATTTGC

CL2883.CONTIG4_ALL GCAAGTGGCACAAGAAGCAGTGCCCAACCCTGTCACAATGCTCGAGAACCTGATATTTGC

CL2883.CONTIG5_ALL GCAAGTGGCACAAGAAGCAGTGCCCAACCCTGTCACAATGCTCGAGAACCTGATATTTGC

XM_027379601 GCAAGTGGCACAAGAAGCAGTGCCCAACCCTGTCACAATGCTCGAGAACCTGATATTTGC

AY321153.2 GCAAGTGGCACAAGAAGCAGTGCCCAGCCCTGTCACAATGCTCGAGAACCTGATATTTGC

CL2833.CONTIG3 GCAAGTGGCACAAGAAGCAGTGCCCAACCCTGTCACAATGCTCGAGAACCTGATATTTGC

CL2883.CONTIG2_ALL GCAAGTGGCACAAGAAGCAGTGCCCAGCCCTGTCACAATGCTCGAGAACCTGATATTTGA

**************************.********************************.

CL2883.CONTIG1_ALL CTACCTTGAATACATTCCCATCCCTGTGAGCGACGCAATCTGGGCCTACTACAACTTCCT

CL2883.CONTIG4_ALL CTACCTTGAATACATTCCCATCCCTGTGAGCGACGCAATCTGGGCCTACTACAACTTCCT

CL2883.CONTIG5_ALL CTACCTTGAATACATTCCCATCCCTGTGAGCGACGCAATCTGGGCCTACTACAACTTCCT

XM_027379601 CTACCTTGAATACATTCCCATCCCTGTGAGCGACGCAATCTGGGCCTACTACAACTTCCT

AY321153.2 CTACCTTGAATACATTCCCATCCCTGTGAGCGACGCAATCTGGGCCTACTACAACTTCCT

CL2833.CONTIG3 CTACCTTGAATACATTCCCATCCCTGTGAGCGACGCAATCTGGGCCTACTACAACTTCCT

CL2883.CONTIG2_ALL CTACCTTGAATACATTCCCATCCCTGTGAGCGACGCAATCTGGGCCTACTACAACTTCCT

************************************************************

CL2883.CONTIG1_ALL TCCACGCTACATCACGGACGCGCTGCCGCCCTACCCACGAACAGCCATGGTGGTTGGCGG

CL2883.CONTIG4_ALL TCCACGCTACATCACGGACGCGCTGCCGCCCTACCCACGAACAGCCATGGTGGTTGGCGG

CL2883.CONTIG5_ALL TCCACGCTACATCACGGACGCGCTGCCGCCCTACCCACGAACAGCCATGGTGGTTGGCGG

XM_027379601 TCCACGCTACATCACGGACGCGCTGNCGCCCTACCCACGAACAGCCATGGTGGTTGGCGG

AY321153.2 TCCACGCTACATCACGGACGCGCTGCCGCCCTACCCACGAACAGCCATGGTGGTTGGCGG

CL2833.CONTIG3 TCCACGCTACATCACGGACGCGCTGCCGCCCTACCCACGAACAGCCATGGTGGTTGGCGG

CL2883.CONTIG2_ALL TCCACGCTACATCACGGACGCGCTGCCGCCCTACCCACGAACAGCCATGGTGGTTGGCGG

************************* **********************************

CL2883.CONTIG1_ALL CACTGAGATCCTCAGCTTCAGCGGCCTTGTTGTGCGAGCACCTCGCTCGCCCTGCAAGCT

CL2883.CONTIG4_ALL CACTGAGATCCTCAGCTTCAGCGGCCTTGTTGTGCGAGCACCTCGCTCGCCCTGCAAGCT

CL2883.CONTIG5_ALL CACTGAGATCCTCAGCTTCAGCGGCCTTGTTGTGCGAGCACCTCGCTCGCCCTGCAAGCT

XM_027379601 CACTGAGATCCTCAGCTTCAGCGGCCTTGTTGTGCGAGCACCTCGCTCGCCCTGCAAGCT

AY321153.2 CACTGAGATCCTCAGCTTCAGCGGCCTTGTTGTGCGAGCACCTCGCTCGCCCTGCAAGCT

CL2833.CONTIG3 CACTGAGATCCTCAGCTTCAGCGGCCTTGTTGTGCGAGCACCTCGCTCGCCCTGCAAGCT

CL2883.CONTIG2_ALL CACTGAGATCCTCAGCTTCAGCGGCCTTGTTGTGCGAGCACCTCGCTCGCCCTGCAAGCT

************************************************************

CL2883.CONTIG1_ALL TCTCCTGGCTGCTCACGGCTCCCACCGCCTCATCATGTCCCACCCGCAAGCCTCAGCCCC

CL2883.CONTIG4_ALL TCTCCTGGCTGCTCACGGCTCCCACCGCCTCATCATGTCCCACCCGCAAGCCTCAGCCCC

CL2883.CONTIG5_ALL TCTCCTGGCTGCTCACGGCTCCCACCGCCTCATCATGTCCCACCCGCAAGCCTCAGCCCC

XM_027379601 TCTCCTGGCTGCTCACGGCTCCCACCGCCTCATCATGTCCCACCCGCAAGCCTCAGCCCC

AY321153.2 TCTCCTGGCTGCTCACGGCTCCCACCGCCTCATCATGTCCCACCCGCAAGCCTCAGCCCC

CL2833.CONTIG3 TCTCCTGGCTGCTCACGGCTCCCACCGCCTCATCATGTCCCACCCGCAAGCCTCAGCCCC

CL2883.CONTIG2_ALL TCTCCTGGCTGCTCACGGCTCCCACCGCCTCATCATGTCCCACCCGCAAGCCTCAGCCCC

************************************************************

CL2883.CONTIG1_ALL GGCACAGCTTGAGCTCAAGACACCAGCAGCCACCGTGATCATCAAGCCTGACTTTGAAGT

CL2883.CONTIG4_ALL GGCACAGCTTGAGCTCAAGACACCAGCAGCCACCGTGATCATCAAGCCTGACTTTGAAGT

CL2883.CONTIG5_ALL GGCACAGCTTGAGCTCAAGACACCAGCAGCCACCGTGATCATCAAGCCTGACTTTGAAGT

XM_027379601 GGCACAGCTTGAGCTCAAGACACCAGCAGCCACCGTGATCATCAAGCCTGACTTTGAAGT

AY321153.2 GGCACAGCTTGAGCTCAAGACACCAGCAGCCACCGTGATCATCAAGCCTGACTTTGAAGT

CL2833.CONTIG3 GGCACAGCTTGAGCTCAAGACACCAGCAGCCACCGTGATCATCAAGCCTGACTTTGAAGT

CL2883.CONTIG2_ALL GGCACAGCTTGAGCTCAAGACACCAGCAGCCACCGTGATCATCAAGCCTGACTTTGAAGT

************************************************************

CL2883.CONTIG1_ALL CCTGGTTAATGGCCAAGCCCTCGGGGGATCCCAGCAAACCATCGGAAACGTTAGGATTGT

CL2883.CONTIG4_ALL CCTGGTTAATGGCCAAGCCCTCGGGGGATCCCAGCAAACCATCGGAAACGTTAGGATTGT

CL2883.CONTIG5_ALL CCTGGTTAATGGCCAAGCCCTCGGGGGATCCCAGCAAACCATCGGAAACGTTAGGATTGT

XM_027379601 CCTGGTTAATGGCCAAGCCCTCNGGGGATCCCAGCAAACCATCGGAAACGTTAGGATTGT

AY321153.2 CCTGGTTAATGGCCAAGCCCTCGGGGGATCCCAGCAAACCATCGGAAACGTTAGGATTGT

CL2833.CONTIG3 CCTGGTTAATGGCCAAGCCCTCGGGGGATCCCAGCAAACCATCGGAAACGTTAGGATTGT

CL2883.CONTIG2_ALL CCTGGTTAATGGCCAAGCCCTCGGGGGATCCCAGCAAACCATCGGAAACGTTAGGATTGT

**********************.*************************************

CL2883.CONTIG1_ALL GAACACAGCCAAGCACATTGAGGTGGGATGTCCCCTGATGAGGGTGATCGTTGCCAAGGC

CL2883.CONTIG4_ALL GAACACAGCCAAGCACATTGAGGTGGGATGTCCCCTGATGAGGGTGATCGTTGCCAAGGC

CL2883.CONTIG5_ALL GAACACAGCCAAGCACATTGAGGTGGGATGTCCCCTGATGAGGGTGATCGTTGCCAAGGC

XM_027379601 GAACACAGCCAAGCACATTGAGGTGGGATGTCCCCTGATGAGGGTGATCGTTGCCAAGGC

AY321153.2 GAACACAGCCAAGCACATTGAGGTGGGATGTCCCCTGATGAGGGTGATCGTTGCCAAGGC

CL2833.CONTIG3 GAACACAGCCAAGCACATTGAGGTGGGATGTCCCCTGATGAGGGTGATCGTTGCCAAGGC

CL2883.CONTIG2_ALL GAACACAGCCAAGCACATTGAGGTGGGATGTCCCCTGATGAGGGTGATCGTTGCCAAGGC

************************************************************

CL2883.CONTIG1_ALL AGGCGAGGCCGTAGCTGTTGAGGCTTCAGGCTGGATCTTTGGACGCGTAGCAGGGCTACT

CL2883.CONTIG4_ALL AGGCGAGGCCGTAGCTGTTGAGGCTTCAGGCTGGATCTTTGGACGCGTAGCAGGGCTACT

CL2883.CONTIG5_ALL AGGCGAGGCCGTAGCTGTTGAGGCTTCAGGCTGGATCTTTGGACGCGTAGCAGGGCTACT

XM_027379601 AGGCGAGNCCGTAGCTGTTGAGGCTTCAGGCTGGATCTTTGGACGCGTAGCAGGGCTACT

AY321153.2 AGGCGAGGCCGTAGCTGTTGAGGCTTCAGGCTGGATCTTTGGACGCGTAGCAGGGCTACT

CL2833.CONTIG3 AGGCGAGGCCGTAGCTGTTGAGGCTTCAGGCTGGATCTTTGGACGCGTAGCAGGGCTACT

CL2883.CONTIG2_ALL AGGCGAGGCCGTAGCTGTTGAGGCTTCAGGCTGGATCTTTGGACGCGTAGCAGGGCTACT

*******.****************************************************

CL2883.CONTIG1_ALL GGGCCCCAACACTGGAGAAATTGCCAATGACCGTCTCATGCCCAGCGGTGCAGCAGCCTC

CL2883.CONTIG4_ALL GGGCCCCAACACTGGAGAAATTGCCAATGACCGTCTCATGCCCAGCGGTGCAGCAGCCTC

CL2883.CONTIG5_ALL GGGCCCCAACACTGGAGAAATTGCCAATGACCGTCTCATGCCCAGCGGTGCAGCAGCCTC

XM_027379601 GGGCCCCAACACTGGAGAAATTGCCAATGACCGTCTCATGCCCAGCGGTGCAGCAGCCTC

AY321153.2 GGGCCCCAACACTGGAGAAATTGCCAATGACCGTCTCATGCCCAGCGGTGCAGCAGCCTC

CL2833.CONTIG3 GGGCCCCAACACTGGAGAAATTGCCAATGACCGTCTCATGCCCAGCGGTGCAGCAGCCTC

CL2883.CONTIG2_ALL GGGCCCCAACACTGGAGAAATTGCCAATGACCGTCTCATGCCCAGCGGTGCAGCAGCCTC

************************************************************

CL2883.CONTIG1_ALL CAACCCCCGCGATTTGGTAGCTGCTTGGCAGGAGGACCCGCAGTGCTCCACCCCTGAGGT

CL2883.CONTIG4_ALL CAACCCCCGCGATTTGGTAGCTGCTTGGCAGGAGGACCCGCAGTGCTCCACCCCTGAGGT

CL2883.CONTIG5_ALL CAACCCCCGCGATTTGGTAGCTGCTTGGCAGGAGGACCCGCAGTGCTCCACCCCTGAGGT

XM_027379601 CAACCCCCGCGATTTGGTAGCTGCTTGGCAGGAGGACCCGCAGTGCTCCACCCCTGAGGT

AY321153.2 CAACCCCCGCGATTTGGTAGCTGCTTGGCAGGAGGACCCGCAGTGCTCCACCCCTGAGGT

CL2833.CONTIG3 CAACCCCCGCGATTTGGTAGCTGCTTGGCAGGAGGAGCCGCAGTGCTCCACCCCTGAGGT

CL2883.CONTIG2_ALL CAACCCCCGCGATTTGGTAGCTGCTTGGCAGGAGGACCCGCAGTGCTCCACCCCTGAGGT

************************************ ***********************

CL2883.CONTIG1_ALL TCCTCATGCTGAGACCACAGTAGGTCGCCTGGTTCAGTGTGAAGCGTTGTTGGGGATTCG

CL2883.CONTIG4_ALL TCCTCATGCTGAGACCACAGTAGGTCGCCTGGTTCAGTGTGAAGCGTTGTTGGGGATTCG

CL2883.CONTIG5_ALL TCCTCATGCTGAGACCACAGTAGGTCGCCTGGTTCAGTGTGAAGCATTATTGGGGATTCG

XM_027379601 TCCTCATGCTGAGACCACAGTAGGTCGCCTGGTTCAGTGTGAAGCGTTGTTGGGGATTCG

AY321153.2 TCCTCATGCTGAGACCACAGTAGGTCGCCTGGTTCAGTGTGAAGCGTTGTTGGGGATTCG

CL2833.CONTIG3 TCCTCATGCTGAGACCACAGTAGGTCGCCTGGTTCAGTGTGAAGCGTTGTTGGGGATTCG

CL2883.CONTIG2_ALL TCCTCATGCTGAGACCACAGTAGGTCGCCTGGTTCAGTGTGAAGCATTATTGGGGATTCG

*********************************************.**.***********

CL2883.CONTIG1_ALL CTCCAGGTGTAACCCAGTGGTTCACCCACAGCCATTCATCAGCATGTGTCACACTGCCCA

CL2883.CONTIG4_ALL CTCCAGGTGTAACCCAGTGGTTCACCCACAGCCATTCATCAGCATGTGTCACACTGCCCA

CL2883.CONTIG5_ALL CTCAAGTTGTAACCCAGTGGTTCACCCACAGCCATTCATCAGCATGTGTCACACTGCCCA

XM_027379601 CTCCAGGTGTAACCCAGTGGTTCACCCACAGCCATTCATCAGCATGTGTCACACTGCCCA

AY321153.2 CTCCAGGTGTAACCCAGTGGTTCACCCACAGCCATTCATCAGCATGTGTCACACTGCCCA

CL2833.CONTIG3 CTCCAGGTGTAACCCAGTGGTTCACCCACAGCCATTCATCAGCATGTGTCACACTGCCCA

CL2883.CONTIG2_ALL CTCAAGTTGTAACCCAGTGGTTCACCCACAGCCATTCATCAGCATGTGTCACACTGCCCA

***.** *****************************************************

CL2883.CONTIG1_ALL CAAGGCTTGCGATGCCGCCCAAGCTTACAGAACCATTTGCTCTCTGAGAGGAGTGGAAGA

CL2883.CONTIG4_ALL CAAGGCTTGCGATGCCGCCCATGCTTACAGAACCATTTGCTCTCTGAGAGGAGTGGAAGA

CL2883.CONTIG5_ALL CAAGGCTTGCGATGCCGCCCAAGCTTACAGAACCATTTGCTCTCTGAGAGGAGTGGAAGA

XM_027379601 CAAGGCTTGCGATGCCGCCCATGCTTACAGAACCATTTGCTCTCTGAGAGGAGTGGAAGA

AY321153.2 CAAGGCTTGCGATGCCGCCCATGCTTACAGAACCATTTGCTCTCTGAGAGGAGTGGAAGA

CL2833.CONTIG3 CAAGGCTTGCGATGCCGCCCATGCTTACAGAACCATTTGCTCTCTGAGAGGAGTGGAAGA

CL2883.CONTIG2_ALL CAAGGCTTGCGATGCCGCCCAAGCTTACAGAACCATTTGCTCTCTGAGAGGAGTGGAAGA

*********************:**************************************

CL2883.CONTIG1_ALL AGTTTTCCCTATGGCGTGCTAACAACACCTGTTGACATGTTAACATAGACATTAAATGTT

CL2883.CONTIG4_ALL AGTTTTCCCTATGGCGTGCTAACAACACCTGTTGACATGTTAACATAGACATTAAATGTT

CL2883.CONTIG5_ALL AGTTTTCCCTATGGCGTGCTAACAACACCTGTTGACATGTTAACATAGACATTAAATGTT

XM_027379601 AGTTTTCCCTATGGCGTGCTAACAACACCTGTTGACATGTTAACATAGACATTAAATGTT

AY321153.2 AGTTTTCCCTATGGCGTGCTAACAACACCTGTTGACATGTTAACATAGACATTAAATGTT

CL2833.CONTIG3 AGTTTTCCCTATGGCGTGCTAACAACACCTGTTGACATGTTAACATAGACATTAAATGTT

CL2883.CONTIG2_ALL AGTTTTCCCTATGGCGTGCTAACAACACCTGTTGACATGTTAACATAGACCTAGAATGTT

**************************************************.*:.******

CL2883.CONTIG1_ALL GACGACTCATTCAGATTTTGGATATGGCTATAG---ATAACTGTATTTTTGTTACGTGAA

CL2883.CONTIG4_ALL GACGACTCATTCAGATTTTGGATATGGCTATAGGTATTAACTGTATTTTTGTTACGTAAA

CL2883.CONTIG5_ALL GACGACTCATTCGGATTTTGGATATGGCTATAGGTATTAACTGTATTTTTGTTACGTAAA

XM_027379601 GACGACTCATTCAGATTTTGGATATGGCTATAGGTATTAACTGTATTTTTGTTACGTGAA

AY321153.2 GACGACTCATTCAGATTTTGGATATGGCTATAGGTATTAACTGTATTTTTGTTACGTAAA

CL2833.CONTIG3 GACGACTCATTCAGATTTTGGATATGGCTATAGGTATTAACTGTATTTTTGTTACGTAAA

CL2883.CONTIG2_ALL GACGACTCATTCAGATTTTGGATATGGCTATAG---ATAACTGTATTTTTGTTACGTGAA

************.******************** :********************.**

CL2883.CONTIG1_ALL TGCATCATAAAATG--TACAAAAAAAAATCCTCAATAAAATTGCAAC-------------

CL2883.CONTIG4_ALL TGCATCATAAAATG--TACAAAAAAAAATCCTCAATAAAATTGCAAC-------------

CL2883.CONTIG5_ALL TGCATCATAAAATG--TACAAAAAAAAATCCTCAATAAAATTGCAAC-------------

XM_027379601 TGCATCAT----------------------------------------------------

AY321153.2 TGCATCATAAAATG--TACAAAAAAAAATCCTCAATAAAATTGCAAC-------------

CL2833.CONTIG3 TGCATCATAAAATGTACAAAAAAAAAAATCCTCAATAAAATTGCAAC-------------

CL2883.CONTIG2_ALL TGCATCATAAAAAAAAAAAAAAAAAAAATCCAAGTACTATTATTTACGAGAAGACAGAAT

********

CL2883.CONTIG1_ALL ------------------------------------------------------------

CL2883.CONTIG4_ALL ------------------------------------------------------------

CL2883.CONTIG5_ALL ------------------------------------------------------------

XM_027379601 ------------------------------------------------------------

AY321153.2 ------------------------------------------------------------

CL2833.CONTIG3 ------------------------------------------------------------

CL2883.CONTIG2_ALL ATCTTGGAAAGCCTCGCAAGTCCATTAATATCTCTCAGAAACCGTATGAATCCAGGAAGA

CL2883.CONTIG1_ALL --------------------------------------------------------CAAC

CL2883.CONTIG4_ALL --------------------------------------------------------CAAC

CL2883.CONTIG5_ALL --------------------------------------------------------CAAC

XM_027379601 ------------------------------------------------------------

AY321153.2 --------------------------------------------------------CAAC

CL2833.CONTIG3 --------------------------------------------------------CAAC

CL2883.CONTIG2_ALL AAAAACTCAAATTCTGCATTATGCAATTTTGATCTTTTTGGATGATAAATTCTTCACATC

CL2883.CONTIG1_ALL TACATTTCTGTTTTGTATTCCATTTGCATATCTG--------------------------

CL2883.CONTIG4_ALL TACATTTCTGTTTTGTATTCCATTTGCATATCTG--------------------------

CL2883.CONTIG5_ALL TACAAAA-----------------------------------------------------

XM_027379601 ------------------------------------------------------------

AY321153.2 TAAAAAAAAAAAAAAAAAAAAAAAAAAAAA------------------------------

CL2833.CONTIG3 ------------------------------------------------------------

CL2883.CONTIG2_ALL AAAAAAACTGTCTTGGATCTCATATGTCATACTGGCGGCAGTGAGATCGTCACATCAGTG

CL2883.CONTIG1_ALL ------------------------------------------------------------

CL2883.CONTIG4_ALL ------------------------------------------------------------

CL2883.CONTIG5_ALL ------------------------------------------------------------

XM_027379601 ------------------------------------------------------------

AY321153.2 ------------------------------------------------------------

CL2833.CONTIG3 ------------------------------------------------------------

CL2883.CONTIG2_ALL AAGGGAGTATTCATTCCGTAATGATTCATTTCCAAAGAATGATAAGTCATTTGTAATGTA

CL2883.CONTIG1_ALL ------------------------------------------------------------

CL2883.CONTIG4_ALL ------------------------------------------------------------

CL2883.CONTIG5_ALL ------------------------------------------------------------

XM_027379601 ------------------------------------------------------------

AY321153.2 ------------------------------------------------------------

CL2833.CONTIG3 ------------------------------------------------------------

CL2883.CONTIG2_ALL TGATTTAAAACAAGATGTTAGAATAGTTAATTATGCTAAATTCACATTATATTAGGCTGC

CL2883.CONTIG1_ALL -------TTTCTATATCTTTTTAAGTATCTAATTCCGGAAACCATAACGAGAAAATATTG

CL2883.CONTIG4_ALL -------TTTCTATATCTTTTTAAGTATCTAATTCCG-----------------------

CL2883.CONTIG5_ALL ------------------------------------------------------------

XM_027379601 ------------------------------------------------------------

AY321153.2 ------------------------------------------------------------

CL2833.CONTIG3 ------------------------------------------------------------

CL2883.CONTIG2_ALL ATCATGTTTTATATATAAATTAATGCATCGTATTCATCTTCATTAAAACATACTCATTTG

CL2883.CONTIG1_ALL GATTACGATGTTGCAGAAATAAGAACATAGATTTATTTGCAGTTGGCAGTATGATATCAA

CL2883.CONTIG4_ALL ------------------------------------------------------------

CL2883.CONTIG5_ALL ------------------------------------------------------------

XM_027379601 ------------------------------------------------------------

AY321153.2 ------------------------------------------------------------

CL2833.CONTIG3 ------------------------------------------------------------

CL2883.CONTIG2_ALL AGTACAGTTTATATAGACCTCAACTGCCATTGTCATTGTCATCATCATTGCTATTCTCGG

CL2883.CONTIG1_ALL TTAGAATACAGAAGGCAACTGATTAAACAAAACAAGTATGACATAAAGATATAAACTATA

CL2883.CONTIG4_ALL ---------------------------------------------------GAAACCATA

CL2883.CONTIG5_ALL ------------------------------------------------------------

XM_027379601 ------------------------------------------------------------

AY321153.2 ------------------------------------------------------------

CL2833.CONTIG3 ------------------------------------------------------------

CL2883.CONTIG2_ALL TTACTTTGTATTAATTAGCTTAATAAAGTATGGATGATTTTTTCTTCCGTTCAACATTTA

CL2883.CONTIG1_ALL CAGTTCTTGTAAAATTTCCCCTTTGGCGTGCTAACAACACCTGTTG--------------

CL2883.CONTIG4_ALL CAGTTCTTGTAAAATTTCCCCTT-------------------------------------

CL2883.CONTIG5_ALL ------------------------------------------------------------

XM_027379601 ------------------------------------------------------------

AY321153.2 ------------------------------------------------------------

CL2833.CONTIG3 ------------------------------------------------------------

CL2883.CONTIG2_ALL CTTTTATTCATCACTTTAAGTAATGAATGGAAGCCTATTTATATTTGTATATATTATAGA

CL2883.CONTIG1_ALL ------------------------------------------------------------

CL2883.CONTIG4_ALL ------------------------------------------------------------

CL2883.CONTIG5_ALL ------------------------------------------------------------

XM_027379601 ------------------------------------------------------------

AY321153.2 ------------------------------------------------------------

CL2833.CONTIG3 ------------------------------------------------------------

CL2883.CONTIG2_ALL GATTAAATATTTTTAATAAAAGTGTACCTCACTTATCTCTCTCTCCTTTGAATTAGATTC

CL2883.CONTIG1_ALL ------------------------------------------------------------

CL2883.CONTIG4_ALL ------------------------------------------------------------

CL2883.CONTIG5_ALL ------------------------------------------------------------

XM_027379601 ------------------------------------------------------------

AY321153.2 ------------------------------------------------------------

CL2833.CONTIG3 ------------------------------------------------------------

CL2883.CONTIG2_ALL ATCATACCCTCTGAAACGTCCCCATGACTTCACCAGAAAAGTAACTTTTTTATGAGAATC

CL2883.CONTIG1_ALL ------------------------------------------------------------

CL2883.CONTIG4_ALL ------------------------------------------------------------

CL2883.CONTIG5_ALL ------------------------------------------------------------

XM_027379601 ------------------------------------------------------------

AY321153.2 ------------------------------------------------------------

CL2833.CONTIG3 ------------------------------------------------------------

CL2883.CONTIG2_ALL TTGAATGTTACAATTACTGTCCTTTTCCTCTGATGATTTTTGGTCGTTCTGTTTTGGTGT

CL2883.CONTIG1_ALL ------------------------------------------------------------

CL2883.CONTIG4_ALL ------------------------------------------------------------

CL2883.CONTIG5_ALL ------------------------------------------------------------

XM_027379601 ------------------------------------------------------------

AY321153.2 ------------------------------------------------------------

CL2833.CONTIG3 ------------------------------------------------------------

CL2883.CONTIG2_ALL TGGCTTCGTTCACCCTTTCAGTATTTAATTATTTATTTTTTCTTGCATTTTCATTTATCA

CL2883.CONTIG1_ALL ------------------------------------------------------------

CL2883.CONTIG4_ALL ------------------------------------------------------------

CL2883.CONTIG5_ALL ------------------------------------------------------------

XM_027379601 ------------------------------------------------------------

AY321153.2 ------------------------------------------------------------

CL2833.CONTIG3 ------------------------------------------------------------

CL2883.CONTIG2_ALL ATTTTTTCATTATTTCCTTTTATGTGTTGTTTTTTTCCCTTTCAATAAAAACATCTCCAA

CL2883.CONTIG1_ALL -------------------------------------------------

CL2883.CONTIG4_ALL -------------------------------------------------

CL2883.CONTIG5_ALL -------------------------------------------------

XM_027379601 -------------------------------------------------

AY321153.2 -------------------------------------------------

CL2833.CONTIG3 -------------------------------------------------

CL2883.CONTIG2_ALL AAGTGTGGATATGAAGGTATATTACTGATAATTTACAAGTGCATTTGTG

Supplementary Figure 1:Nucleotide sequence information for the LvV11 gene obtained from different L. vannamei transcriptomes. XM_027379601 and AY321153.2 are the vitellogenin sequences of L. vannamei retrived from the GenBank database. These sequences are analyzed by multiple sequence alignment as described in the Material and Methods section of the MS.
