# Supplementary material for: Investigation of Gene Sequence Divergence, Expression Dynamics, and Endocrine Regulation of the Vitellogenin Gene Family in the Whiteleg Shrimp Litopenaeus vannamei
Source: Front Endocrinol (Lausanne). 2020 Nov 19;11:577745. doi: 10.3389/fendo.2020.577745 (PMC7711153; doi:10.3389/fendo.2020.577745)
Supplement: Supplementary file 2 [file DataSheet_2.docx]

CLUSTAL 2.1 multiple sequence alignment

1Vg270421 ATATATATATATATATATATATATATATATAATATATATATATAATACAATATATGTATA

1Vg294361 -------TACTTGTATAGATATAATTATG-----TGTCTGTATATAACAGTATATCTATC

LVAN2Vgcpromoter ------------ATAAACATAGGGTGGCTGCAAATTTATGCGGCCAGCTATACTGCTACT

LVAN2Vgbpromoter -----TGAAATTCTATGTATTTTTTGATGATGGGTCGGTTAGCGTAGCAAAACCCCCCCG

**:. **: : . * * . :.*:.:* .

1Vg270421 TACACACATACATATCTATCTATCTATCTATCTATCTATCTATCTATCATCTATATATCT

1Vg294361 TATACACGTAAGTACATACAAATATGTATAGG-------CATATATTCATATAAATAGAT

LVAN2Vgcpromoter AGTAAAAGAACTCAATATATGAAGTAGTAAAAGAAAGAAAAATTACTAAGATATAGAGAC

LVAN2Vgbpromoter GCGCGCTATACATGTGTGAGGAACCATTAGAATGTGTCCCGTTGAAGAGGGGGAGAGAGA

. . .:*. . : *: . :. . :: : .. .:. .

1Vg270421 ATCTATCTATATATATATGTATGTATGTATATATATACATATTTATACAAACACACACAC

1Vg294361 ATATATGTACACACACAGACACACACACACACACACACACACACACACACACACACACAC

LVAN2Vgcpromoter AAAAAATATTGCACAAAAATCACATCATCTTATGACACAGATAAGGGATGAGATATTCGA

LVAN2Vgbpromoter GAGGAAGGAGAGAGAGAGAGAGAGAGAGAGAGAGAGGAAGAGAGGAGGAAGGAGGGAAAG

.: *: : . * * * . . : . . : : * ..* * : . . : . * . :..

1Vg270421 ACACATATATATACATATATATATTCATATATT-TTATACATATACATATAAACATTCAT

1Vg294361 ACATATATATATATATATATATATATATATATATATATATATATATATATATATGTGAAC

LVAN2Vgcpromoter GATATGAAGTAGATAGCATAAAATGCAGATAAG--------GTCAGCTTGGTACAGGCGG

LVAN2Vgbpromoter AGGAGGAAGGAAGGGAGGAGGGATTGAGGAGGG-AGAGGGAGAGGAGAGGGAGGGTGAGG

. *:. * . . : . ** * .:. : . : .:. . ..

1Vg270421 CATATATACATATATATATATATATATTATATATATATATATATAATATATATATATATA

1Vg294361 AATAGAGAGAGAGAGAGAGACAGAGGAGAGAGAGAGAGAGACAGAGTATATATATATATA

LVAN2Vgcpromoter AGTGAAATGCGGTGACGTCATGCATATTCGAAATACCGTGCTAGATAATGCATATTTTAA

LVAN2Vgbpromoter GGAGAGGGAGAGGGAGAGGGAGAGGGAGGGAGAGGGAGAGGGAGAGGAAGAGGAGGAAGA

.:. . . . . . . .: * * . . : * * *:. . * :: *

1Vg270421 TATATATATATATATATACAGACAGAGAGAGATACAGAGAGAGAAGAGAGAGAGAGAAGA

1Vg294361 TATATATATATATATATATATATATATATATATATATATATATATATATATATATATATA

LVAN2Vgcpromoter TTTCATCATTATCATTTTCATTTATTAATTTAAATTTGATTCAATGTATGGTATTTTTTA

LVAN2Vgbpromoter GGAAGAGGAAGAGGAAGAGGGAGAGGGAGAGGGTAGAGGGAGGGAGGGAGGGAGGGGAGA

:. : .:: : .:: : . : * * : . : . .:. .:. : : *

1Vg270421 GAGAGAGAGAGAGAGAGAGAGAGAGAGAGAGAGAGAGAGAGAGAGAGAGAGAGAGATAAT

1Vg294361 TATATAGATGGATAGATAGATATAGATATATATATATAGA-----------------TAT

LVAN2Vgcpromoter TGTAATACTG-AGAGATAATGTAAATGTCTCTTTTCTGGT-----------------ACC

LVAN2Vgbpromoter AGAAAAAGAGAGAAGAGAGAGAGAGAGAGAGAGAGAGAGAGAGAG------------AGA

. * :. . . *** *.: : *.: : : : : . .*: :

1Vg270421 ATATGCATTCATATATATATACATATATATATATATATTATATATACATATACATACATA

1Vg294361 ATTTGTTTTCATGGGTATCTATCTATCTATCTATCTAT---ATATCTATATCTATATATA

LVAN2Vgcpromoter TATTTTCTCCTTTTTGGTCTCATATTGCATATATTTTTTTCATTTTCGCATAGATATATA

LVAN2Vgbpromoter GAGAGAGAGAGAGAGAGAGAGAGAGAGAGAGAGAGAGAGAGAGAGAGAGAGAGAGAGAGA

: : : . : .: : : : .: :.: : : * : . * . * * * *

1Vg270421 TGTATACATATATACATATACATATATTAATATATGTTTTATATATATATACATATACAT

1Vg294361 TATATATATATATATATATACATATAT---------------ATATACATACATATATGT

LVAN2Vgcpromoter AGACTGCTTTGGAATTGAATGTTTTCAG-----TTTTATTTACAGTTTTGCTCTTTACTT

LVAN2Vgbpromoter GAGGGGCGGGGGGGGAGGAAAAAGTAGAAAG----------GAAGAGAAAGGGATAGTTA

. . . . : .:: :: *. .:.: : :::. :

1Vg270421 ATATATATATGTATATATATATATATATATATAT-ATATATATATATATATATATATATA

1Vg294361 AAATATATATGTATATATATATATATATATATATTATATATATATATATATATATATATA

LVAN2Vgcpromoter ATATCTTAATGTAATTATTCATTTATTCATATGGTAATTCAGAATACCTTCGCGGGGCCG

LVAN2Vgbpromoter TACATAGAGAGAAATAAATAAAATGAAAAAATGAGAAATCGATACACATGTACACACAAA

::.: : :.:*:*:::*:: *::*.:: *:**. *::*. .:* * .* . . . . .

1Vg270421 TATATATATATATATATATATATATATATAAATGCACAAACATATATACATATATATATG

1Vg294361 TATATATATATATATGTATATATATATATATATATATATATATATATATATATATATATA

LVAN2Vgcpromoter ATTACAATGATACTGGTAACGGTGAGCTCCCCTAAGCCTATACTACCCATAGATGTAGGA

LVAN2Vgbpromoter TATATATATATATATATATATATATATATATATATATATATATATATATATATATATATA

::** *:: *** : .**:. .*.:. : . .*. . .:* * ::. . ::.:: ::. .

1Vg270421 TATATATATGTATATATATATTATATATATATATATATATATATAATATATATATATATA

1Vg294361 TAATATATATATATATATGTTTATGTGTGTATATATATGTATACAGAGAGAGAGAAAGAG

LVAN2Vgcpromoter CGTACCCTGTGGTAGCCCGATCGTAGGGGAAGTACCAGGGACATTGAGGGGTAAAATGTG

LVAN2Vgbpromoter TATATATATATATATCACGCACACACCCATATATATATATATATATATATATTATATATA

.:: : :: : . . .:* ::. * . * * : : :.:*:.:.

1Vg270421 TATTATATATATTTATCTATACACACACACACACACACACACACACACACACACACACCA

1Vg294361 AGAGAGAGAGAGAGAGAGAGAGAGAGAGAGAGAGAGAGAGAGAGAGAGAGAGAGAGAGAG

LVAN2Vgcpromoter AAATATTTATACAGAAAATATCACCCCCCCCCAACCCCTGTCCTGGAAG-----ACAAAG

LVAN2Vgbpromoter TATTATATATATATATATATAATATATATATATATATATATATATATATATATGTATGTG

:.: * : * * : * . : : : : : : : : : .

1Vg270421 CACACACAACATATATATATATATATATATATATATATATATATACATATACATATACAT

1Vg294361 AGAGAGAGAGAGATACATATATATATATATATATATATATATATATATATATGTGTGTGT

LVAN2Vgcpromoter AATCCTCCACGTATTCACGGCAGGATAGTGCTTAGAATGGGTGGTGGATGTGTGGTGTGG

LVAN2Vgbpromoter TGTGTGTGTG-TGTGTGTGTGTGTGTGTGTGTGTGTGTGTGTACATTGTGATAAATAGAT

. : .* . . :. .*. * :.:.*. .*. : : : .*. .

1Vg270421 ATACATATACATATACATATAGATACATGCATACATACATACATACATACACACACACAC

1Vg294361 GTGTGTGTGTGTGTGTGTGTGTGTGTGTGTGTGTGTGTGTGTGTGTGTGTGTGTGTGTGT

LVAN2Vgcpromoter GTGGCTGAAGCTCTTAGTCTGGAGGATAGGGTGACGCCGCGGTGCACAGTGTAACCTTAC

LVAN2Vgbpromoter AAAACAAAAAAATAGAGAGATAGAGAGAGAGATAGAGAGAGAGAATCAGAAAGAAATAGA

.:. :.:. : : .: : . . :* .: . . :. . . .

1Vg270421 ACACACACACACCACACACACACATACACAC-ATATATATATATATATATATATATATAT

1Vg294361 GTGTGTGTGTG---TGTGTAAATATATATAT-ATATATATATATATATATATATATATAT

LVAN2Vgcpromoter ATTTAGGTCCTGGGTTTATGAAGTGATGGGA-ATG-GTCGACAACCGGGTGTGTGCATGC

LVAN2Vgbpromoter GGAAGATAGAGGGAGAGGTAAACTACTGAAAGATAGATGCAATACAGACAGAGTAGATAA

. . . ..* : . . . **. .* * ::. . :.:.*. **.

1Vg270421 ATATATATATATATATATATATATATATATATATAT-TATATATATATGTCAGTGTGTTT

1Vg294361 ATATATATATATATATATATATATGTATATGTATATGTATATGTATATATATATATATAT

LVAN2Vgcpromoter ATCGGAGAGAATGTTGCAGTCCTTCCAGTAATCGGGGATCATGATCGGTCAAGGGACGAC

LVAN2Vgbpromoter TGAGTAAGAGACAAAAGACACACAAAAAAAGAAGAAAAAAAAGTAAAAATAAAGATAAAA

: . :. . * .:: : :. : * ::.:. . :: *:.:: . .:. .: :

1Vg270421 GTGTGTGCATCCATCTATCTATATATGTGTATATACATACATATAATATATATATATAAT

1Vg294361 ATATATATATATATATATATATATATATATATATATATGCATATAAATGTACAAATATGT

LVAN2Vgcpromoter TCGTTTCTTAACATCTGCAAGTAGG-GTCTAGCTGGTTCAGTCCTCGACCGCGACGAGGG

LVAN2Vgbpromoter AAGTAAAAAGGGCAATTTAAAGCGAGATCCAGAATTTAGAGAAAAATAGACCTTTACGCC

.* : : .:.* .:. . . .* * .: :: ..:. :. : . :

1Vg270421 ATATATATATTATATATATTATATATATATATATATATATATATATATATATATATATAT

1Vg294361 GTATATATATTTATATATACGGATATATATACATATGCACACACACACACACACACACAC

LVAN2Vgcpromoter AAAGATCGCGTGTCTCCCCCAGAACTTCCCGTCAGTAACTCCACCCACGTTGTATTTGTG

LVAN2Vgbpromoter TCATTTCCATAATCTTCTGCCTCCAATCTTTCAAGAAAAACGACGACCGCCCAATCGCGT

* :*. . : : : . .:: .:.:. . . * . . : :

1Vg270421 ACATAGA--CACATACACACACACTCACACACACACACAGACACACACACACACACACAC

1Vg294361 ACACACA--CACACACACACACACACACACACACACACACACACACACACCAGCATACTC

LVAN2Vgcpromoter GCGGTCG--AACTTGCACTCTTCTACTTGTACTTCGACG-ACTTTTTCTTAGCCCTAATC

LVAN2Vgbpromoter CTTATCAGCCAAATTAATCCAAAATTTCATCCGCCAGCTGATTAGAATCGAGCTGATTAG

: . .*.: .* *: . : : . .* . .* * : : . : :

1Vg270421 ACACACACACACA-------------------CACACACACACACACACACACACATATG

1Vg294361 ACGGACACATACAGAAAGGGAAAAAGAAA-TGCGCACACAGACACACACACACATACACA

LVAN2Vgcpromoter ACATGCTGCTGATCCATGGCCCAGGGAATGCTTACCCCTTGGCAGAAACTCGACCTGTTG

LVAN2Vgbpromoter GCTAACACGGATGATAGTCATAAGGGGGAAAAAAGTACTGTAAACACTGAACACTTTGTA

.* .*: . . .. ..* *.: :. .. : .

1Vg270421 TATATATATATGTATACATATATATGT---------------------TATATATATATA

1Vg294361 TATATGTATGTGTATACATGTGTATATATGTGTGTGTCTTTGTGTGTGATTGTGTGTCTC

LVAN2Vgcpromoter GAGATGCTCGCAAAGCCCAGTGCAAGCTCTCGCCACATGGGCTGGACCGTGAATGCCTCA

LVAN2Vgbpromoter CCCTGAGGTGTGTACACGGCAGGTTGCACGGGTGATGTACACTCTTTAACCGACCCCCGC

. : . . .:* .* :. ::. .: .

1Vg270421 TATATATATATATATATATATATATATATATATATATATATATATATATATATAGTATAT

1Vg294361 AGTCCGTTTATATATATATATATATATATATATATATATATATATATATATATA-TATAT

LVAN2Vgcpromoter GGATCAACAGGATATTCATATCGAGGTGAGCCCACTAATAGAAGGATCTCATTGATATCG

LVAN2Vgbpromoter GGTAGTACATAATGCAGTAATGATAGTGATGACGTCTGGCTTTCAGGCTTCAGAATCACC

.: : : **. : ::** : .*.: . . :. . :: . .* : . *.:.

1Vg270421 AT---------------------GTATATACATATATATATGAATATATATGTATATGTA

1Vg294361 AT---------------------ATATATATATATATATATATATATATATATATATATA

LVAN2Vgcpromoter AGGCGGACCAGCGACACAGCCGCCTCTCCTGCTTCGCGCCGCAAACTTGTTGCCCGTCTG

LVAN2Vgbpromoter AGTTG------------------CTATTCACCTGCGAACACCTGACTCTGGACAGGAGGT

* *.* : .* . . . :.:.* . . .:

1Vg270421 TATATATATATATATATATATATATATATATATATATATATATATATATTATATATATAA

1Vg294361 TATATATATATATATATATATTATATATTATATATATGTATGTATGTATTATATACATAA

LVAN2Vgcpromoter AGCAGGATTTGCCCGCACTTTCCGCAGCCCATCCCTGAGCAGTACTTGGAAAGTGTACAG

LVAN2Vgbpromoter GGGGGAGGTGGGGGAGGTGGGGGAGGTGGGGAGGTGGGGAGGTGGGGGAGGTGGAGGAGG

. . . * : . . .*. . .:. . . ..

1Vg270421 TG-----CACACACACACACACT-ACATACACACATATATATATATAT-----ATATACA

1Vg294361 ATGCACACACACACACACACACACACACACACACACACATACATATAT-----ATATATA

LVAN2Vgcpromoter TATTATGCTGAAGCACTGACGACACAGCACACGTAATGCTTCATCTAAGACCAATCTACA

LVAN2Vgbpromoter TAGGNTATATATATATATATATATATATATATATTATAATATATATAT-----ATATATA

: : * . * : * . . . * * . : : .*: **.**: **.** *

1Vg270421 TATATATATATTATATATATATAATGTAATATATATATATATATATATATATATATATAT

1Vg294361 TATATATATATATATATATATATATATATATATAATATATATATATATATATATATATAT

LVAN2Vgcpromoter TGGTTTTGTACACTGATATTGTGGTGCCATACACATTTTATAACAATTTAGTGCGTTATG

LVAN2Vgbpromoter TATATATATATATATATATATATATATATATATATATATATATATATATATAATATATAT

*. :*:*.** : : ::::: : .*. .::: : ::::::::: :::::: :. .*:::

1Vg270421 ATATATATATATAATATATATAATATCTGTATATACATATAAATAATATATATATGAATA

1Vg294361 ATAAGTATATATATCTATATATATACATATATATACACACATATGTTTAAACACACACGC

LVAN2Vgcpromoter ATAGCCTGGAACACCTGTAATGAAAAGCATG-GTAGTTACCTGCAATGGAACACTATTGA

LVAN2Vgbpromoter ATATATATATATATATATATATATATATATATATATATATATATATATATATATATATAT

*** : .:* * : :::: *:* .*. .** : * .:. .:: :: : : :

1Vg270421 TATAATATATA-TATATATATACACATATATATAT--------------------AATAT

1Vg294361 ACATGTGTGTG-TGTCTGTGTGTGCATGTGTGTGTGTGTGGGGGGTGAGTGTGCGTATGT

LVAN2Vgcpromoter TCAAGTGGATGGTTGCTGTGAGTAGTTGTTTACTGAGAG-------------TTTGTTTA

LVAN2Vgbpromoter ATATATATATA-TATATATATATATATATATATAT-------------------ATATAT

: ::.*. .*. * .*.*.:. . :*.* *. :* :

1Vg270421 ATATATATATATATATATATATATATATATTATATACATATACACATATATATATATATA

1Vg294361 GTGTGGGTATGTATATGTATATATATAACATATGGACATCTATATCTATACATTTATTCA

LVAN2Vgcpromoter TTGGGAGTGTTTTGTTATAAGTGATTGTCGTTGTGAATGGTTGTACTTTGTGAGTGGCTG

LVAN2Vgbpromoter ATATATATATATAATATATATATATATATATATATATATATATATATATATATATATATA

*. . .*.* *: :: ::: : ::: : *: * : *: : .*:*. .: *. .

1Vg270421 TACATA-----TCATATATATATATATATATATATATGTATATGTGTGTGTGTATGCTTG

1Vg294361 TTAATAGATAGATATACATACATACATCATGTATCATATATATGTGTGTGTGTATGCTTG

LVAN2Vgcpromoter TTCGCTGTGAGCGATGGTTCGGTGTGAGCGGTTGTTCGCTGTGAGCGNTATATATATATA

LVAN2Vgbpromoter TATATATATATATATATATATATATATATATATATATATATATATATATATATATATATA

*: . : **. :*. .*. .: :: : . : : . *.*.***. :*.

1Vg270421 TTCTTTTTTCTGTACCTTCCATTTACCGTTGATTTATCGCGTACTTG--GATAAGCTTCA

1Vg294361 TTCTTTTTTCTGTACCTTCCATTTACCGTTGATTTATCGCGTACTTG--GATAAGCTTCA

LVAN2Vgcpromoter TATATATATATATATATGTAAGGGTATATGAATGATTCATATTATTGACGAGAATTCCCT

LVAN2Vgbpromoter TATATATATATATATATAGTAGGGTATATGAATGATTCATATTATTGACGAGAATTCCCT

*: :*:*:*.*.** .* * :. .* .** ::**. .*:.*** ** ** *:

1Vg270421 GAAAAGAGCTCGGTGATTCACGGCGACATGCAAACGTCACTTAAAACTCTCGAGGAGGTG

1Vg294361 GAAAAGAGCTCGGTGATTCACGGCGACATGCAAACGTCACTTAAAACTCTCGAGGAGGTG

LVAN2Vgcpromoter GCAGAAAGAACTTTGTCAAAAACTGAATTGCTTACAG-ACTGTCTAGTCCAGATTAGAT-

LVAN2Vgbpromoter TCAGAAAGAACTTTGTCAAAAACTGAATTGCTTACAG-ACTGTCTAGTCCAGATTAGATA

.*.*.**.:* **: :.*.. **.:***::**. *** :.:* ** .** **.*

1Vg270421 ACGCTGCGATCCCGGTCAGCTGGAGATGGCGACGGGTATATAAGGCCGAGGCGCAGGGGG

1Vg294361 ACGCTGCGATCCCGGTCAGCTGGAGATGGCGACGGGTATATAAGGCCGAGGCGCAGGGGG

LVAN2Vgcpromoter ---ATGCAATTCG--TGTGACTCATGTCATGTCGGGTATAAAAGGCAGAGACTCAGCAGG

LVAN2Vgbpromoter TGACGGGCAATTCG-TGTGACTCATGTCATGTCGGGTATAAAAGGCAGAGACTCAGCAGG

. * *: * :*. * .* . *:********:*****.***.* *** .**

1Vg270421 TCGGGCAGTAGTCTTGGTGATCGCTCAGAACCACCATG

1Vg294361 TCGGGCAGTAGTCTTGGTGATCGCTCAGAACCACCATG

LVAN2Vgcpromoter TCGTCCAGTAGTCGTGGTGACCGGGTAGAGCCACCATG

LVAN2Vgbpromoter TCGTCCAGTAGTCGTGGTGACCGGGTAGAGCCACCATG

*** ******** ****** ** ***.*****

Supplement File 2: Comparison of the promoter region of LvVg1 (i.e. 1Vg27042 and 1Vg294361) and LvVg2 (i.e. LVAN2Vgcpromoter, LVAN2Vgbpromoter). The 200-300 bp upstream promoter region of LvVg1 and LvVg2 genes are highly homologous. The homologies decrease at the more distal region (i.e. >350 bp onward). The ATG indicates the first codon of all Vg gene and the potential TATA factor binding site is shown by the red boxes.
