# Supplementary material for: Investigation of Gene Sequence Divergence, Expression Dynamics, and Endocrine Regulation of the Vitellogenin Gene Family in the Whiteleg Shrimp Litopenaeus vannamei
Source: Front Endocrinol (Lausanne). 2020 Nov 19;11:577745. doi: 10.3389/fendo.2020.577745 (PMC7711153; doi:10.3389/fendo.2020.577745)
Supplement: Supplementary file 3 [file Table_1.docx]

Supplement Table 1: Primers used in the study

| Primer name | Sequence |
| --- | --- |
| Lv1Vgd1F | GAA CCA CCA TGA CGA CCT CAC AGC T |
| Lv1Vga1F | CCA GCC AAC ATG ACG ACC TCA ACT CT |
| Lv1Vgb1F | ACA TGA CGA CCT CAA CTC TCC TCT TCG |
| Lv1Vge1F | ACC ATG ACG ACC TCA CAG CTC TTC TT |
| Lv1Vg200R | CTT GTC CTG ACA CGT GAT GGC GGT |
| Lv2Vg1F | AAC GGC TTG CTC GGC CAT GGC AAC AT |
| Lv2Vg200R | CTT GTC CTG ACA GGT GAT GGT GGT GT |
| Lv3Vg2380F | AAG CAT CGG GCA CGT CAG AGT TCA G |
| Lv3VgR | GAA TAA CGT TCC TCG ATC CTC CCG T |
| rtLv1VgF1 | AAG TCC AAG GTC CAG CTC AAG GG |
| rtLv1VgR1 | GGG TGC CAT CCA CCT TGG TAT TCT T |
